# Treatment time and circadian genotype interact to influence radiotherapy side-effects. A prospective European validation study using the REQUITE cohort

**DOI:** 10.1016/j.ebiom.2022.104269

**Published:** 2022-09-18

**Authors:** Adam J. Webb, Emily Harper, Tim Rattay, Miguel E. Aguado-Barrera, David Azria, Celine Bourgier, Muriel Brengues, Erik Briers, Renée Bultijnck, Jenny Chang-Claude, Ananya Choudhury, Alessandro Cicchetti, Dirk De Ruysscher, Maria Carmen De Santis, Alison M. Dunning, Rebecca M. Elliott, Laura Fachal, Antonio Gómez-Caamaño, Sara Gutiérrez-Enríquez, Kerstie Johnson, Ramón Lobato-Busto, Sarah L. Kerns, Giselle Post, Tiziana Rancati, Victoria Reyes, Barry S. Rosenstein, Petra Seibold, Alejandro Seoane, Paloma Sosa-Fajardo, Elena Sperk, Begoña Taboada-Valladares, Riccardo Valdagni, Ana Vega, Liv Veldeman, Tim Ward, Catharine M. West, R. Paul Symonds, Christopher J. Talbot, the REQUITE Consortium

**Affiliations:** a Department of Genetics and Genome Biology, University of Leicester, Leicester, UK; b Leicester Cancer Research Centre, University of Leicester, Leicester, UK; c Fundación Pública Galega Medicina Xenómica, Santiago de Compostela, Spain; d Instituto de Investigación Sanitaria de Santiago de Compostela, Spain; e Department of Radiation Oncology, Montpellier Cancer Institute, Université Montpellier, Inserm U1194, Montpellier, France; f Institut de Recherche en Cancérologie de Montpellier, Université Montpellier, Inserm U1194, Montpellier, France; g Patient advocate, Hasselt, Belgium; h Department of Human Structure and Repair, Ghent University, Ghent, Belgium; i Division of Cancer Epidemiology, German Cancer Research Center (DKFZ), Heidelberg, Germany; j Translational Radiobiology Group, Division of Cancer Sciences, University of Manchester, Manchester Academic Health Science Centre, Christie Hospital, Manchester, UK; k Prostate Cancer Program, Fondazione IRCCS Istituto Nazionale dei Tumori, Milan, Italy; l Maastricht University Medical Center, Department of Radiation Oncology (Maastro clinic), GROW School for Oncology and Developmental Biology, Maastricht, the Netherlands; m Department of Radiation Oncology, Fondazione IRCCS Istituto Nazionale dei Tumori, Milan, Italy; n Centre for Cancer Genetic Epidemiology, Department of Oncology, University of Cambridge, Cambridge, UK; o Wellcome Sanger Institute, Wellcome Genome Campus, Hinxton, UK; p Department of Radiation Oncology, Complexo Hospitalario Universitario de Santiago, SERGAS, Santiago de Compostela, Spain; q Hereditary Cancer Genetics Group, Vall d'Hebron Institute of Oncology (VHIO), Vall d'Hebron Barcelona Hospital Campus, Barcelona, Spain; r Department of Medical Physics, Complexo Hospitalario Universitario de Santiago, SERGAS, Santiago de Compostela, Spain; s Departments of Radiation Oncology and Surgery, University of Rochester Medical Center, Rochester, New York, NY, United States; t Radiation Oncology Department, Vall d'Hebron Hospital Universitari, Vall d'Hebron Barcelona Hospital Campus, Barcelona, Spain; u Department of Radiation Oncology, Department of Genetics and Genomic Sciences, Icahn School of Medicine at Mount Sinai, New York, USA.; v Medical Physics Department, Vall d'Hebron Hospital Universitari, Vall d'Hebron Barcelona Hospital Campus, Barcelona, Spain; w Department of Radiation Oncology, Medical Faculty Mannheim, University of Heidelberg, Mannheim, Germany; x Department of Oncology and Haematology-Oncology, Universita degli Studi di Milano, Italy; y Biomedical Network on Rare Diseases (CIBERER), Spain; z Department of Radiation Oncology, Ghent University Hospital, Ghent, Belgium; aa Patient advocate, NCRI CTRad consumer, UK

**Keywords:** Circadian rhythm, Radiotherapy, Genetics, Breast cancer

## Abstract

**Background:**

Circadian rhythm impacts broad biological processes, including response to cancer treatment. Evidence conflicts on whether treatment time affects risk of radiotherapy side-effects, likely because of differing time analyses and target tissues. We previously showed interactive effects of time and genotypes of circadian genes on late toxicity after breast radiotherapy and aimed to validate those results in a multi-centre cohort.

**Methods:**

Clinical and genotype data from 1690 REQUITE breast cancer patients were used with erythema (acute; *n=*340) and breast atrophy (two years post-radiotherapy; *n=*514) as primary endpoints. Local datetimes per fraction were converted into solar times as predictors. Genetic chronotype markers were included in logistic regressions to identify primary endpoint predictors.

**Findings:**

Significant predictors for erythema included BMI, radiation dose and *PER3* genotype (OR 1.27(95%CI 1.03-1.56); *P <* 0.03). Effect of treatment time effect on acute toxicity was inconclusive, with no interaction between time and genotype. For late toxicity (breast atrophy), predictors included BMI, radiation dose, surgery type, treatment time and SNPs in *CLOCK* (OR 0.62 (95%CI 0.4-0.9); *P <* 0.01), *PER3* (OR 0.65 (95%CI 0.44-0.97); *P <* 0.04) and *RASD1* (OR 0.56 (95%CI 0.35-0.89); *P <* 0.02). There was a statistically significant interaction between time and genotypes of circadian rhythm genes (*CLOCK* OR 1.13 (95%CI 1.03-1.23), *P <* 0.01; *PER3* OR 1.1 (95%CI 1.01-1.2), *P <* 0.04; *RASD1* OR 1.15 (95%CI 1.04-1.28), *P <* 0.008), with peak time for toxicity determined by genotype.

**Interpretation:**

Late atrophy can be mitigated by selecting optimal treatment time according to circadian genotypes (e.g. treat *PER3* rs2087947C/C genotypes in mornings; T/T in afternoons). We predict triple-homozygous patients (14%) reduce chance of atrophy from 70% to 33% by treating in mornings as opposed to mid-afternoon. Future clinical trials could stratify patients treated at optimal times compared to those scheduled normally.

**Funding:**

EU-FP7.

**Research in context**

**Evidence before this study**

We searched PubMed for articles describing human studies of circadian rhythm effects on side effects from radiotherapy for breast cancer up to 18 June 2021. The search terms included were “radiotherapy” and “breast” and “circadian” and “toxicity” or “side effect” or “adverse reaction” or “skin reaction”. The search resulted in seven articles, of which four are reviews and one is a study on tumour response in radiotherapy patients with bone metastases. Two articles describe observational studies on whether time of radiotherapy affects toxicity: Noh et al 2014 which found that in 395 breast cancer patients skin reactions were most common in the afternoon group, but with no genetic analysis. The other is our paper Johnson et al 2019, which described analysis of a cohort of 535 patients.

**Added value of this study**

This study improves on previous studies through use of a large pan-European cohort of patients (*n=*1690) from multiple recruitment locations, and a time-of-day analysis based on solar time (continuous) rather than AM/PM stratification. Treatment time as a predictor of late atrophy is confirmed, while we also find new significant interactions between multiple circadian genes (*PER3, CLOCK* and *RASD1*) and treatment time with respect to late atrophy (*P*=0.005-0.02). Crucially, we determine that peak time of toxicity is determined by circadian genotypes.

**Implications of all the available evidence**

This data show that radiation toxicity, particularly late atrophy, can be sensitive to treatment time dependant on circadian genotypes. Allocating time of treatment according to genotype can therefore reduce occurrence of late radiation toxicities. Remarkably, we predict triple-homozygous patients (14% of patients) reduce their chance of atrophy from 70% to 33% by treating in the morning as opposed to mid-afternoon. This personalised approach to radiation therapy requires testing in a large international trial.

Alt-text: Unlabelled box

## Introduction

Ten year all-stage survival from breast cancer has almost doubled in England and Wales from 1971 to 2017. Age standardised 10-year survival increased from 40% to 76%^1^ and has improved across Europe.^2^ The reasons for the improvement in survival in breast cancer are complex and include earlier diagnosis (including screening) and improvements in surgery, radiotherapy, chemotherapy, and hormone treatment. Radiotherapy is given to most patients treated after breast conservation surgery and selected patients after mastectomy but can lead to life-long side-effects (toxicity). Amongst the 2215 patients in the START-B trial, physician assessed incidence of breast shrinkage at 10 years after treatment was 26.2% (40Gy) and 31.2% (50Gy), with induration in the tumour bed in 14.3% and 17.4% respectively.^3^

Circadian rhythms are daily oscillations of metabolic activity driven by the master clock of the suprachiasmatic nucleus in the brain, with peripheral clocks in every organ system. Tissues at risk of late damage in the breast include fat, skin, and connective tissue. It has been known for over 50 years that skin cell division is under circadian control.^4^ S-phase of the cell cycle has been shown to be maximal close to noon by flow cytometry and in tritiated thymidine studies.^5^ The cell cycle in skin is under genetic control particularly by the PER (period) family of genes. Janich and colleagues identified five peaks of circadian gene activity over 24 hours and were related to keratinocyte differentiation.^6^ The dynamics of diurnal cycles are less well understood in subcutaneous tissue but there is considerable evidence of a circadian clock present in connective tissues and in cells in the extra-cellular matrix.^7^ Recently, circadian rhythms in genes expressed in the epidermis have been used to generate a reliable phase biomarker for an individual's circadian cycle.^8^ The cyclic nature of skin and subcutaneous cell division has implications when assessing the normal tissue effects of irradiation of the human breast as radio-sensitivity varies considerably as cells progress through the cell cycle, with relative radio-resistance in the S-phase and increased radiosensitivity in late G2 and mitosis.^9^

There is good evidence for the benefit of delivering various chemotherapeutic agents at specific times-of-day based on the circadian rhythm of the patient, especially in reducing side effects. The literature on circadian rhythm effects on radiotherapy has until now been mixed.^12^ Clinical studies in several cancer types, including head & neck, cervical and prostate cancers, have shown a difference in radiotherapy side effects according to time of treatment. The studies however are inconsistent and hard to compare as they use various time analysis methods.^10^

Evidence already exists linking afternoon treatment to increased acute side-effects from radiotherapy in breast cancer patients,^11^ while we previously investigated genetic circadian rhythm variants for association with acute and late toxicity and receipt of radiotherapy in the morning,^12^ Late radiation toxicity was assessed in the LeND cohort of 535 patients recruited retrospectively and scored using the late effects in normal tissue-subjective objective management analysis (LENT-SOMA). Acute side-effects were assessed in a prospectively recruited cohort of 343 patients from Leicester scored according to CTCAEv4. Genotyping was carried out for candidate circadian rhythm variants. In the LeND cohort, patients who had radiotherapy in the morning had significantly increased late toxicity in univariate (*P*=0.03) and multivariate analysis (*P*=0.01). Acute effects in the prospective Leicester cohort were also statistically significantly increased in univariate (*P*=0.03) but not multivariate analysis. Increased late effects in the LeND cohort receiving morning radiotherapy were associated with the *PER3* variable number tandem repeat (VNTR) 4/4 genotype (*P=*0.006) and the *NOCT* rs131116075 AA genotype (*P=*0.005).^12^

Previously we divided participants into groups in which >66% of fractions were delivered morning or afternoon, and a mixed group where fractions were delivered across the day. This simple cut-off was necessitated by sample size but assumes that radiotherapy circadian effects vary linearly over the time-window of treatment (08.00-18.00 in Leicester). That assumption is not well-founded, and rendered void by the wider time-window of patients treated at multiple centres. A further problem is that there is no consensus in the literature over the time cut-offs, with other studies having used various times to define morning and evening groups.^10^

The aim of this study was to extend our previous investigation to include over 2,000 breast cancer patients recruited into the multi-centre prospective REQUITE cohort study with a minimum follow up of two years. The objectives were to evaluate a time-of-day effect upon the incidence of acute and late radiation toxicity in this larger prospective cohort, and to validate the association of previously investigated genetic circadian variants with late toxicity.

## Methods

### Participants

**Figure 1:**
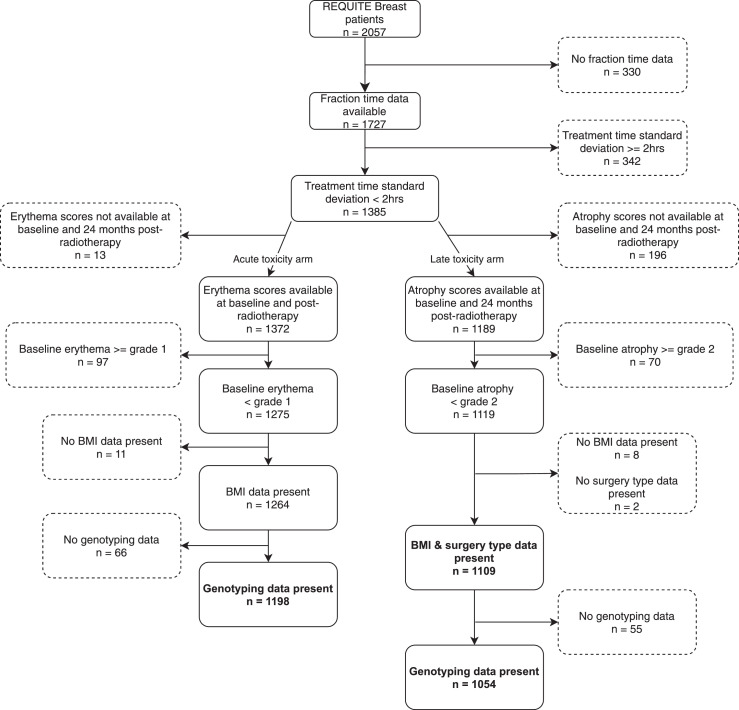
STROBE diagram showing selection of patient cohorts for analysis of erythema and atrophy.

The REQUITE (https://requite.eu) cohort study recruited breast, prostate, and lung cancer patients from eight countries between April 2014 and March 2017 and continue to be followed up.^13^ A centralised database containing pre-treatment, treatment and follow up data is available to validate predictive models and potential biomarkers in over 4400 patients. DNA (*n=*4409) was stored in a centralised biobank. Participants were drawn from the breast cohort of REQUITE, comprising 2057 women. Recruiting centres are listed in Table 1. Inclusion criteria included women over the age of 18 with a confirmed diagnosis of breast cancer and no evidence of distant metastases treated with radiotherapy with curative intent. Exclusion criteria included prior irradiation at the same site, male breast cancer patients, concomitant chemo-radiation, bilateral tumours and mastectomy (see^13^ for full list). Tumour excision was performed by one of three surgical techniques: wide local excision (lumpectomy; removal of all grossly visible tumour mass plus a narrow margin of normal tissue); quadrantectomy (removal of a quarter of the breast tissue, sometimes with excision of overlying skin and underlying fascia); or segmentectomy (removal of an anatomical segment of the breast containing the tumour). Figure 1 (STROBE diagram) provides an outline of patient selection and eligibility for our analyses.

**Table 1:** Summary statistics for cohort.

Characteristic	Overall, *N =* 1,727	Barcelona, *N =* 193	Gent, *N =* 295	Leicester, *N =* 343	Leuven, *N =* 252	Mannheim, *N =* 37	Milan, *N =* 102	Montpellier, *N =* 405	Santiago, *N =* 100
Latitude (degrees)		41.4	51.1	52.6	50.9	49.5	45.5	43.6	42.9
Longitude (degrees)		-2.2	3.7	-1.1	4.7	8.5	9.2	3.9	-8.5
Shortest day (hours)		9.2	7.9	7.6	7.9	8.1	8.7	8.9	9
Longest day (hours)		15.2	16.6	16.8	16.5	16.3	15.7	15.4	15.3
Genotypes	1,640 (95%)	176 (91%)	287 (97%)	312 (91%)	249 (99%)	37 (100%)	98 (96%)	382 (94%)	99 (99%)
Age (years)	58 (11)	56 (12)	58 (11)	61 (11)	58 (11)	52 (9)	53 (10)	59 (11)	57 (11)
BMI (kg/m^2^)	26.4 (5.6)	26.4 (5.3)	25.9 (4.6)	28.3 (6.6)	25.7 (4.6)	25.3 (7.3)	23.7 (4.0)	25.9 (5.6)	29.2 (5.6)
(no data)	17	0	3	6	5	0	1	1	1
Tumour histological grade									
1	330 (20%)	34 (18%)	45 (16%)	81 (24%)	50 (20%)	7 (21%)	16 (16%)	71 (18%)	26 (26%)
2	890 (53%)	105 (55%)	129 (47%)	175 (51%)	130 (53%)	17 (50%)	57 (56%)	231 (58%)	46 (46%)
3	462 (27%)	53 (28%)	99 (36%)	87 (25%)	64 (26%)	10 (29%)	28 (28%)	94 (24%)	27 (27%)
(no data)	45	1	22	0	8	3	1	9	1
Menopausal status									
Pre	404 (24%)	66 (34%)	50 (17%)	41 (12%)	52 (22%)	13 (35%)	53 (52%)	108 (27%)	21 (21%)
Post	1,167 (69%)	116 (60%)	217 (74%)	245 (72%)	168 (72%)	20 (54%)	41 (40%)	289 (73%)	71 (71%)
Peri	127 (7.5%)	10 (5.2%)	28 (9.5%)	55 (16%)	14 (6.0%)	4 (11%)	8 (7.8%)	0 (0%)	8 (8.0%)
(no data)	29	1	0	2	18	0	0	8	0
Diabetes	105 (6.1%)	11 (5.7%)	18 (6.1%)	29 (8.5%)	13 (5.2%)	2 (5.4%)	3 (2.9%)	23 (5.7%)	6 (6.0%)
Smoking status									
Never	946 (55%)	117 (61%)	181 (62%)	182 (53%)	126 (55%)	18 (49%)	73 (72%)	189 (47%)	60 (60%)
Ex-smoker (before cancer diagnosis)	446 (26%)	34 (18%)	70 (24%)	111 (32%)	65 (28%)	5 (14%)	8 (7.8%)	132 (33%)	21 (21%)
Ex-smoker (since cancer diagnosis)	73 (4.3%)	13 (6.7%)	9 (3.1%)	13 (3.8%)	7 (3.0%)	4 (11%)	11 (11%)	10 (2.5%)	6 (6.0%)
Current smoker	240 (14%)	29 (15%)	34 (12%)	37 (11%)	33 (14%)	10 (27%)	10 (9.8%)	74 (18%)	13 (13%)
(no data)	22	0	1	0	21	0	0	0	0
Surgery type									
Segmentectomy / Quadrantectomy	841 (49%)	191 (99%)	35 (12%)	22 (6.4%)	0 (0%)	31 (91%)	102 (100%)	361 (89%)	99 (99%)
Wide local excision	882 (51%)	2 (1.0%)	260 (88%)	321 (94%)	252 (100%)	3 (8.8%)	0 (0%)	43 (11%)	1 (1.0%)
(no data)	4	0	0	0	0	3	0	1	0
Neoadjuvant chemotherapy (anthracycline)	138 (8.0%)	38 (20%)	7 (2.4%)	15 (4.4%)	34 (13%)	7 (19%)	5 (4.9%)	12 (3.0%)	20 (20%)
Neoadjuvant chemotherapy (non-anthracycline)	153 (8.9%)	45 (23%)	7 (2.4%)	15 (4.4%)	39 (15%)	9 (24%)	5 (4.9%)	12 (3.0%)	21 (21%)
Adjuvant chemotherapy (anthracycline)	352 (20%)	50 (26%)	82 (28%)	56 (16%)	24 (9.5%)	4 (11%)	37 (36%)	65 (16%)	34 (34%)
(no data)	1	0	0	0	0	1	0	0	0
Adjuvant chemotherapy (non-anthracycline)	374 (22%)	65 (34%)	88 (30%)	32 (9.3%)	38 (15%)	4 (11%)	39 (38%)	71 (18%)	37 (37%)
BED (acute toxicity) (Gy)	65 (11)	71 (9)	58 (6)	53 (5)	73 (5)	75 (7)	69 (7)	72 (9)	67 (10)
BED (late toxicity) (Gy)	94 (14)	100 (12)	88 (8)	78 (6)	109 (7)	104 (9)	96 (8)	99 (13)	96 (14)
Boost	1,158 (67%)	161 (83%)	221 (75%)	35 (10%)	251 (100%)	30 (81%)	83 (81%)	301 (74%)	76 (76%)
Intensity-modulated radiation therapy (IMRT)	898 (52%)	29 (15%)	264 (89%)	305 (89%)	216 (86%)	29 (78%)	1 (1.0%)	54 (13%)	0 (0%)
Mean treatment time (hours from local midnight)	12.63 (2.74)	13.89 (3.60)	11.75 (1.76)	12.60 (1.89)	11.76 (2.02)	10.49 (1.72)	12.40 (2.38)	12.94 (3.18)	14.83 (3.34)
Mean treatment time (hours from solar midnight)	11.35 (2.74)	12.08 (3.63)	10.38 (1.81)	12.00 (1.94)	10.48 (2.12)	9.51 (1.72)	11.31 (2.41)	11.61 (3.22)	12.53 (3.41)
Treatment time s.d. > 2 hrs	349 (20%)	30 (16%)	126 (43%)	100 (29%)	42 (17%)	16 (43%)	0 (0%)	15 (3.7%)	20 (20%)
Baseline erythema	116 (6.8%)	5 (2.6%)	11 (3.7%)	14 (4.1%)	9 (3.6%)	0 (0%)	1 (1.0%)	73 (18%)	3 (3.0%)
(no data)	15	0	0	3	4	1	1	6	0
Erythema deterioration	340 (20%)	83 (43%)	33 (11%)	58 (17%)	43 (17%)	5 (14%)	41 (41%)	66 (17%)	11 (11%)
(no data)	15	0	0	3	4	1	1	6	0
Baseline atrophy	76 (5.2%)	20 (11%)	0 (0%)	6 (2.6%)	6 (2.5%)	0 (0%)	0 (0%)	39 (11%)	5 (6.0%)
(no data)	254	5	58	108	11	5	13	38	16
Atrophy deterioration	514 (35%)	39 (21%)	94 (40%)	102 (43%)	119 (49%)	9 (28%)	30 (34%)	78 (21%)	43 (51%)
(no data)	254	5	58	108	11	5	13	38	16

n (%); Mean (SD).

### Sample size determination and data management

Sample size for this study was determined by the availability of data from suitable patients recruited to the REQUITE Project. Patients were treated according to local radiotherapy protocols but information was collected on standardised project forms. REQUITE recruitment centres were invited to provide the time and date of each radiotherapy fraction giving a total of 1727 potential participants, of which 1640 had genotyping data. Data from patients were collected and input into electronic case report forms (CRFs) – further details are available elsewhere.^13^

### Toxicity endpoints

We used the most common acute and late (two-years after radiotherapy) side-effects in the cohort: erythema and atrophy (Table 2). Baseline and 24-month grading, including atrophy, were scored by the physicians according to CTCAE v4.00. Breast photos were taken at each time-point. Patients with baseline (post-surgery, pre-radiotherapy) scores > 0 for erythema and > 1 atrophy were removed from our analyses. Baseline erythema in these patients was likely due to surgically induced inflammation rather than radiotherapy. Similarly, the 76 patients with gross atrophy before radiotherapy probably already had marked tissue loss from surgery. Erythema was dichotomised with a cut off >= grade 2. Atrophy deterioration was dichotomised for worsening of >=1 grade at 24 months versus baseline. No blinding procedures on outcomes were carried out.

**Table 2:** Selected SNPs in circadian genes. For SNPs selected from GWAS studies we also show associated odds ratio for morningness, plus *p*-value.

Gene	Reason tested	Chromosome	rs number	Position (NCBI37)	Alleles(alelle2 frequency)	Allele 2 morningness odds radio(95%CI)*p*-value(GWAS-derived SNPs only)
*PER3*	Validation gene. Strong LD with VNTR used in Johnson et al (2019)^12^	1	rs2087947	7851093	C/T (0.31)	N/A
*CLOCK*	Validation Johnson et al (2019)^12^	4	rs1801260	56301369	A/G (0.27)	N/A
*NOCT*	Validation from Johnson et al (2019)^12^	4	rs13116075	139930032	A/G (0.15)	N/A
*VAMP3*	Close to *PER3*. Statistically significant in GWAS study ^14^	1	rs11121022	7836659	A/C (0.42)	1.07 (1.04-1.09) *P* = 2 × 10^-8^
*PER2* (120kb upstream)	Statistically significant in GWAS study^14^	2	rs55694368	239317692	G/T (0.11)	0.86 (0.81-0.9) *P* = 2.6 × 10^-9^
*RASD1*	Statistically significant in GWAS study^14^	17	rs11545787	17398278	G/A (0.25)	1.08 (1.05-1.11) *P=* 1.4 × 10^-8^
*HCRTR2*	Statistically significant in GWAS study^14^	6	rs35833281	55021561	G/C (0.23)	0.92 (0.9-0.95) P = 3.7 × 10^-9^

### Genetic analysis

Genotyping was carried out centrally using Illumina Infinium OncoArrays on 600k single nucleotide polymorphisms (SNPs) and subjected to standard quality control procedures. A total of 7,409,901 SNP variants were imputed with minor-allele frequency (MAF) > 0.05 using the 1000 Genomes Project data. A power calculation assuming *n=*1054, atrophy frequency 35%, power = 0.80 and OR=1.3 (assumes Johnson et al.^12^ OR is over-inflated) shows for different minor allele frequencies (MAFs) we have: MAF 0.05 α=0.25, MAF 0.1 α=0.05, MAF 0.2 α=0.005, suggesting adequate power for up to 10 SNPs. Candidate SNPs were selected from Johnson et al 2019^12^ (SNPs in *PER3, NOCT* and *CLOCK*) where a genotype-modulated treatment time effect has previously been reported. SNPs were also selected from further circadian rhythm genes (*VAMP3, RASD1, PER2, HCRTR2*) (Table 2) on the basis of significant association with morningness phenotype, a well-known circadian role and a minimum minor allele frequency of 0.05.^14^ We used a polygenic risk score (PRS) for chronotype calculated in ldpred^15^ using SNPs from a combined UK Biobank / 23andMe GWAS of 697,828 individuals.^16^

### Circadian analysis

**Figure 2:**
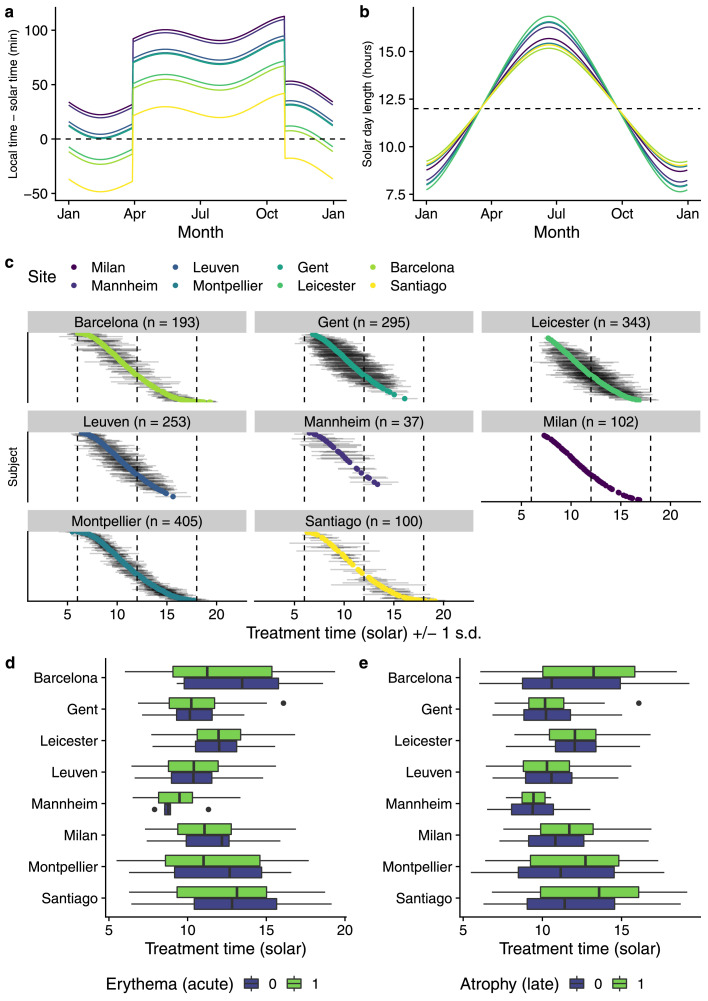
a) Comparison of local time and solar time. Local time/solar time difference is affected by longitude, time zone and daylight savings adjustments. Note that sites sharing the same or similar longitudes may have different time zones (for example, between the UK and Europe). In this figure, the local time in Milan in late July is approximately 100 minutes ahead of solar time. Small seasonal variations in the difference between local and solar time are seen due to the Earth's elliptical orbit around the sun. b) Seasonal variation in day length for each site. Dashed line indicates the spring and autumn equinoxes, where day and night are of equal length. As all centres span a small range of temperate latitudes, only a small inter-site variation in daylength is seen, although sites closer to the equator will see smaller differences in the maximum and minimum day lengths. c) Mean treatment time (x-axis, continuous) for each patient (y-axis, nominal), grouped by site and ordered (top to bottom) by mean treatment time. Grey lines indicate +/- 1 s.d. (in hours), after removing all patients with >= 2 s.d. (*n =* 1385). d) Univariate comparison of solar-adjusted treatment time for each site and development of erythema as a dichotomous variable (*n =* 1198). e) Univariate comparison of solar-adjusted treatment time for each site and deterioration in atrophy at 24 months compared to baseline as a dichotomous variable (*n =* 1054). Box and whisker plots show box = interquartile range, line = median, whiskers = range (without outliers), dots = outliers.

Local time and date of each radiotherapy fraction were converted into solar time (whereby solar noon is when the sun reaches its zenith for a given location) using the photobiology R package^17^ v.0.10.5, thus adjusting for longitude, time zone, daylight savings and seasonal solar orbit variations (Figure 2a). Mean treatment time as a continuous predictor was used as a more powerful alternative to grouping by morning/afternoon used in other studies. As the signals from the suprachiasmatic nucleus are influenced by the amount of light reaching the retina, additional metrics day length (Figure 2b), maximum solar elevation and standard deviation of the fraction times (Figure 2c) were calculated. Milan patients were given radiotherapy at the same time each day, but times in other centres varied (Figure 2c). Patients with a wide range of treatment times (SD > 2 h) were excluded as it is not possible to analyse the contribution of time-of-day in such patients. As cell cycle phase varies throughout the day, we identified 00:00 and 15:30 as optimal solar time offset for erythema and atrophy by constructing models at varying times and selecting for minimal Akaike information criteria (AIC).

### Statistical methodology

To adjust for different radiotherapy regimens, dose was calculated as the biologically effective dose (BED), a widely used method of comparing different radiation treatment fractionation schedules which assigns a numerical score based on the linear quadratic model. The alpha value is the number of logs of cell kill per Gy (Gray) from the linear portion of the cell survival curve and the beta is the number of logs of cell per Gy squared from the quadratic component. BED is the product of the number of fractions (*n*), dose per fraction (*d*), including any boost doses, and a factor determined by the dose and α/β ratio (10 Gy for acute effects and 3 Gy for late effects^9^):

$$BED=n\;d\left(1+\frac{d}{\alpha /\beta }\right)$$

Statistical analysis was carried out in R version 4.0.3 ^18^ with the tidyverse packages used for data handling and visualisations.^19^ Graphs were produced with ggplot2 3.3.2.^20^ Pearson's Chi-squared test or Wilcoxon rank sum tests were performed for univariate analyses. All multivariate analyses were conducted using logistic regression (generalised linear model (GLM) with binomial link function). Optimal logistic regression base models for erythema deterioration and atrophy deterioration were derived using stepwise predictor removal and replacement (stepAIC function from the R MASS package^21^ version 7.3, using both forward and backward replacement). Age, diabetes, smoking status, BMI, surgery type, BED, use of IMRT, neoadjuvant and adjuvant chemotherapy (both anthracycline and non-anthracycline), day length (season), latitude, solar zenith angle (accounts for both season and latitude), mean treatment time (solar) and standard deviation of treatment time were used in the initial model. An optimised model was selected based on minimising AIC and removing any further statistically non-significant predictors. SNPs (gene dosages, 0-2) were systematically and individually added to this base model (including an interaction between SNP and treatment time) to identify genetic predictors with statistically significant effects. No corrections were made for multiple testing. To address potential effects of unmodelled confounding factors between treatment centres (e.g. biases in grading and other unadjusted differences) a secondary, confirmatory analysis using mixed effects logistic regression models was constructed using R's lme4 package (version1.1)^22^ with site (recruitment centre) as the sole random intercept. Model performance was assessed using ROC curve analysis carried out using tidymodels^23^ on 100 repeats of 4-fold cross-validation sets stratified by atrophy. AUC was taken as the mean of all folds and repeats. No blinding procedures for predictors were carried out.

Missing data were not imputed and incomplete records were omitted in their entirety when carrying out logistic regression. Figure 1 demonstrates inclusion/exclusion criteria and results *n* for both erythema and atrophy models. All reported effect sizes, confidence intervals and p-values were obtained through logistic regression (LR), unless otherwise stated. We have applied the TRIPOD, STROBE and STREGA reporting guidelines.

### Ethics

The REQUITE study was reviewed and approved by North West - Great Manchester East Ethics Committee (UK, reference 14 NW 0035) and by the local Ethics Committees of all participating centres. The patients provided their written informed consent to participate in this study and for the publication of the data included in this article.

### Role of funders

Funders had no role in study design, data collection, data analyses, interpretation, or writing of the report.

## Results

### Cohort description

The characteristics of the cohort are shown in Table 2 and results for univariate analyses (chi-squared and Wilcoxon rank-sum tests for erythema and atrophy) are shown in Supplementary table 1. Variables known to affect radiotherapy toxicity in breast vary between clinical centres and therefore need to be controlled for include radiotherapy treatment parameters (dose, boost doses, use of intensity-modulated radiation therapy (IMRT)), diabetes, smoking status, BMI and type of pre-radiotherapy surgery.

### Acute toxicity analysis

Erythema was analysed as the most common acute toxicity (recorded post-radiotherapy) in the REQUITE breast cohort and considered as a dichotomous measure (CTCAE score 0-1 vs >=2, after excluding patients with baseline >0 due to the possible effect of surgical inflammation) (Figure 2d). Patients with a treatment time SD > 2 hours were also removed (*n=*1198 remaining).

Logistic regression analysis with fixed effects was performed. As expected, the final models showed statistically significant effects of BMI and radiation dose to the breast (including boost doses) (Table 3). We also observed a strong and statistically significant effect of fraction time variation (OR 0.64 (95%CI 0.49-0.83), *P <* 0.001 (LR)), although this was skewed by eliminating patients with treatment time SD > 2 hours and higher rates of erythema at Milan where patients were treated at the same time each visit. To account for clustering (ICC ρ = 0.1), mixed-effect models were fitted with treatment centre as a random intercept. As expected, treatment time variation in these models became statistically non-significant.

**Table 3:** Logistic regression models for acute erythema (0/1).

	Non-SNP model	+ rs2087947 C/T (*PER3*) (fixed effects)	+ rs2087947 C/T (*PER3*) (random effects)
BMI	**1.04 (1.02 – 1.07)** ***P =* 0.0005**	**1.04 (1.02 – 1.07)** ***P =* 0.0006**	**1.05 (1.03 – 1.08)** ***P <* 0.0001**
Biological equivalent dose to breast (BED) (Gy)	**1.02 (1.01 – 1.03)** ***P =* 0.0004**	**1.02 (1.01 – 1.03)** ***P =* 0.0005**	**1.02 (1.01 – 1.04)** ***P =* 0.003**
Mean treatment time (hours from solar midnight)	**1.05 (0.999 – 1.1)** ***P =* 0.05**	**1.05 (1 – 1.1)** ***P =* 0.043**	1.02 (0.966 – 1.07) *P =* 0.49
Standard deviation of treatment times (solar time)	**0.64 (0.491 – 0.832)** ***P =* 0.0009**	**0.645 (0.495 – 0.839)** ***P =* 0.001**	0.812 (0.545 – 1.08) *P =* 0.2
rs2087947 (dosage, T allele)		**1.27 (1.03 – 1.56)** ***P =* 0.024**	**1.27 (1 – 1.54)** ***P =* 0.027**
(Random Effect) Treatment centre stdev:__(Intercept)			0.59
N	1198	1198	1198
ROC AUC (95% CI) a	0.61 (0.55–0.67)	0.62 (0.56–0.68)	
AIC	1,244	1,241	1,203

Logistic regression. Statistics shown: odds ratio (95% CI).

a see Supplementary Figure 1.

Models containing mean solar zenith angle across fractions were constructed to address the possibility that increased erythema may be influenced by increased UV exposure from sunlight (even through clothing). Such an approach accounts for both latitude and time of year (season), but, as well as day length also considered independently, ultimately did not prove statistically significant.

Model optimisation revealed an optimum origin for time was at solar midnight. Time of day (solar) was statistically significant (*P=*0.05 (LR)) in the fixed effects model, but not the mixed effects model (*P=*0.49 (LR)). Expanding our model to incorporate individual SNPs from previously investigated circadian genes^12^ (*CLOCK, NOCT* and *PER3*) revealed a statistically significant effect (OR 1.27 (95%CI 1.03-1.56), *P=*0.02 (LR)) from a SNP intronic to the *PER3* gene (Table 3). This SNP (rs2087947) shows strong linkage disequilibrium (LD) (|D′|=0.95, r^2^=0.74) with the previously examined *PER3* VNTR and thus serves as a suitable proxy.

In light of the more recent publication of a major GWAS study on self-reported chronotype,^14^ four further SNPs were included in our analysis (Table 2). None of these additional SNPs were statistically significant, even when the model was adapted to include genotype x time interactions (not shown).

### Late toxicity analysis

We repeated the analysis method used for acute erythema with atrophy of the breast at 24 months post-radiotherapy as the most common late radiation toxicity amongst our participants (35%). Atrophy deterioration (dichotomous) was defined as worsening atrophy by one grade or more compared to baseline. Patients with post-operative baseline atrophy >= grade 2 were excluded from the study leaving *n=*1109. We investigated whether mean solar time of radiotherapy from the circadian peak of 15:30 affected atrophy using multivariate logistic regression. The optimal model contained BMI, surgery type and radiation dose to the breast as statistically significant co-variates. Solar-adjusted time of radiotherapy was statistically significant, with atrophy decreasing with time away from 15:30 (OR 0.94 per 1 hour (95% CI 0.89-0.99); *P <* 0.02 (LR)) apparently favouring morning treatment for reduced side effects. We did not see any effect of day length (season), latitude, or a combination metric (solar zenith angle), although we note that all our sites span only a narrow band of temperate latitudes with similar seasonal day length variation (Table 2; Figure 2b).

To test the effects of candidate circadian SNPs the model was modified by adding a single SNP at a time for the subset of patients with genotyping data (*n=*1054). ROC curve analysis (Supplementary Figure 1) shows no significant change in AUC when adding *CLOCK* and *PER3* SNPs independently or in combination (AUC=0.64-0.65). Nonetheless, *CLOCK* SNP rs1801260 and *PER3* SNP rs2087947 were both found to be statistically significant (OR 0.62 (95%CI 0.41-0.92) and 0.65 (95%CI 0.44-0.97); *P <* 0.04) (LR) (Table 4)) although *NOCT* was not found to be statistically significant (not shown).

**Table 4:** Logistic regression models for late atrophy (dichotomous).

	No SNPs	+ *rs1801260 A/G* (dosage) *(CLOCK)*	+ *rs2087947 C/T* (dosage) *(PER3)*	+ *rs11545787 G/A* (dosage) *(RASD1)*	+ 3 SNPs unweighted PRS	+ GWAS PRS
BMI (kg/m^2)	**1.06 (1.03 – 1.08)** ***P <* 0.0001**	**1.06 (1.03 – 1.09)** ***P <* 0.0001**	**1.06 (1.04 – 1.09)** ***P <* 0.0001**	**1.06 (1.03 – 1.09)** ***P <* 0.0001**	**1.06 (1.04 – 1.09)** ***P <* 0.0001**	**1.06 (1.03 – 1.09)** ***P <* 0.0001**
Biological equivalent dose (BED) to breast (Gy)	**1.02 (1.01 – 1.03)** ***P =* 0.0008**	**1.02 (1.01 – 1.03)** ***P =* 0.002**	**1.02 (1.01 – 1.03)** ***P =* 0.001**	**1.02 (1.01 – 1.03)** ***P =* 0.0017**	**1.02 (1.01 – 1.03)** ***P =* 0.0014**	**1.02 (1.01 – 1.03)** ***P =* 0.0017**
Surgery: wide local excision vs. segmentectomy / quadrantectomy	**2.13 (1.64 – 2.77)** ***P <* 0.0001**	**2.17 (1.66 – 2.84)** ***P <* 0.0001**	**2.19 (1.68 – 2.87)** ***P <* 0.0001**	**2.22 (1.7 – 2.91)** ***P <* 0.0001**	**2.17 (1.66 – 2.85)** ***P <* 0.0001**	**2.21 (1.69 – 2.89)** ***P <* 0.0001**
Mean treatment time from solar 1530 (hours)	**0.939 (0.888 – 0.991)** ***P =* 0.02**	**0.874 (0.81 – 0.942)** ***P =* 0.0005**	**0.88 (0.812 – 0.953)** ***P =* 0.0016**	**0.867 (0.8 – 0.937)** ***P =* 0.0004**	**0.772 (0.691 – 0.859)** ***P <* 0.0001**	**0.93 (0.879 – 0.984)** ***P =* 0.012**
SNP (dosage; allele 2)		**0.616 (0.408 – 0.923)** ***P =* 0.02**	**0.653 (0.437 – 0.971)** ***P =* 0.037**	**0.557 (0.345 – 0.889)** ***P =* 0.015**		
SNP x time from solar 1530 interaction		**1.13 (1.03 – 1.23)** ***P =* 0.009**	**1.1 (1.01 – 1.2)** ***P =* 0.035**	**1.15 (1.04 – 1.28)** ***P =* 0.0076**		
3 SNP unweighted polygenic risk score (PRS) for reduced atrophy^a^					**0.619 (0.483 – 0.79)** ***P =* 0.0001**	
3 SNP unweighted PRS for reduced atrophy^a^ x time from solar 1530 interaction					**1.12 (1.06 – 1.18)** ***P =* 0.0004**	
Morningness PRS (from morningness GWAS)						1.32 (0.864 – 2.01) *P =* 0.2
N	1109	1054	1054	1054	1054	1052
ROC AUC (95% CI)	0.64 (0.6–0.7)	0.65 (0.6–0.7)	0.64 (0.6–0.7)	0.65 (0.6–0.7)	0.66 (0.61–0.71)	
AIC	1,383	1,310	1,312	1,310	1,299	1,310

Logistic regression. Statistics shown: odds ratio (95% CI).

Atrophy (0/1) - change of >= 1 grade atrophy. Sample includes patients with baseline atrophy up to and including Grade 1.

a Unweighted PRS score is generated by summing the dosage of allele 2 (0-2) for each of the circadian SNPs rs1801260 (*CLOCK*), rs2087947 (*PER3*) and rs11545787 (*RASD1*) separately analysed. As each SNP shows all overall decrease in atrophy for allele 2 when considered independently of treatment time, they can be simply combined into a single risk score (0-6), where the ‘risk’ is overall reduced atrophy.

**Figure 3:**
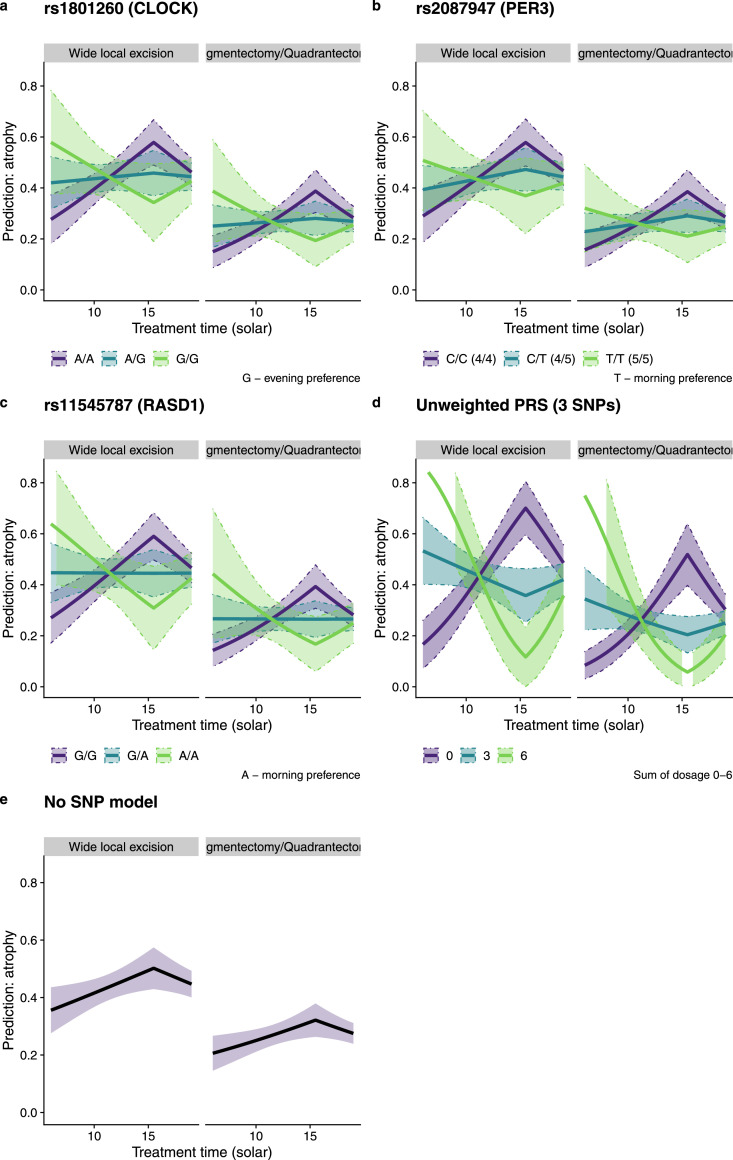
Probability of deterioration in atrophy for a patient of mean BMI (26.5) and biological equivalent dose (BED) (94 Gy) for both wide local excision and segmentectomy / quadrantectomy surgery, derived from fixed-effect logistic regression models incorporating a) rs1801260 in *CLOCK*, b) rs2087947 in *PER3*, c) rs11545787 in *RASD1*, d) Combined unweighted PRS score generated by combining minor allele dosages of all three SNPs where probabilities are shown for triple homozygous (0 - estimated 1427 per 10,000 patients; 6 - estimated 4 per 10,000 patients) and triple heterozygous genotypes (3 – estimated 632 per 10,000 patients), e) no additional SNPs. Shaded areas bounded by dashed lines show 95% confidence intervals. A clear relationship is seen between treatment time and the probability of developing atrophy (e), which is modified or even reversed by genotype (a,b,c,d). For example, in panel b), the lowest probability of atrophy when treated in the morning is seen for patients with a C/C genotype; patients with the T/T genotype have the lowest probability of atrophy for afternoon treatment.

Most intriguing was a statistically significant interaction between treatment time and each of the *CLOCK* and *PER3* SNPs, with treatment time effect direction dependant on genotypes (Figure 3). We expanded our investigations to include SNPs from genes with well-known circadian roles previously highlighted in a large-scale circadian GWAS and exhibiting a minor allele frequency (MAF) > 0.05 (*PER2, RASD1, VAMP3* and *HCRTR2*). Of these, only rs11545787 in *RASD1* was significant with a large effect (OR 0.56 (95%CI 0.35-0.89); *P=*0.015 (LR); ROC AUC=0.65). Again, the interaction with treatment time was present, with the less common A allele (MAF 0.25, associated with morning chronotype) predicting higher rates of atrophy for patients treated in the morning (OR 1.15 (95%CI 1.04-1.28); *P=*0.008 (LR)).

Finally, to assess the impact of genetically determined chronotype on a genome-wide basis, we generated PRS scores for morningness (based on a chronotype GWAS from ∼86k individuals^14^) but did not see any statistically significant effect on atrophy rates either independently of treatment time, nor with interactions (Table 4).

Treatment time and genotype results remained statistically significant in a mixed-effect model with treatment site as a random intercept (ICC for treatment centre ρ = 0.11, see Supplementary Table 2 for mixed-effect logistic regression results), and in a sensitivity analysis where patients with baseline atrophy scores > 0 were removed (remaining *n=*732). Surgery type was not statistically significant in our mixed-effect models due to the strong association with treatment centre. We therefore cannot rule out the possibility that the surgery type effect is actually just an artifact of differences in grading of atrophy between treatment centres.

## Discussion

This study provides further evidence for an effect of treatment time-of-day on the occurrence of radiotherapy side effects in breast cancer patients. We re-analysed circadian variants, SNPs within the *PER3* and *CLOCK* genes, and found associations with the late radiotherapy side effect of breast atrophy two years after treatment and identified a third associated circadian variant in the *RASD1* gene.

Acute side effects show an association with time-based parameters in fixed-effect models; however, those effects are no longer seen when treatment centre is considered as a random effect, agreeing with the findings from our previous study.^12^ Late side-effect analysis (fixed and mixed) shows that average treatment time is a statistically significant predictor, with a maximum at 15:30 solar time and an odds ratio of 0.94 for each hour before or after that. In this analysis it would be expected that atrophy would be reduced by the same amount at 13:30 and 17:30, and most substantially reduced in those patients treated in the morning. As few patients were treated late in the evening we cannot judge what effect treatment at night would have. Furthermore, there is a statistically significant positive interaction between genotype and time effects in the regression analysis. This interaction can be better described by graphical presentation of atrophy prediction based on our logistic regression models at different times of day for each genotype (Figure 3).

We report that circadian genes can have a notable effect on radiosensitivity. For *PER3* the T/T genotype of rs2087947 (in strong LD with the 5/5 allele of the previously investigated nearby VNTR associated with extreme morning preference^24^) shows the most reduced atrophy when treated at 15:30. The A/A genotype of rs11545787 in *RASD1* also associated with morningness^14^ shows the same effect. For both genes, the opposite homozygotes are predicted to show highest rates of atrophy when treated at 15:30. Conversely, the G/G allele of rs1801260 in *CLOCK*, where previous studies conflict on an association with evening preference chronotype,^25^, ^26^, ^27^ shows lowest rates of atrophy for afternoon treatment. Heterozygotes for each of the three genes show no statistically significant variation in rates of atrophy across time, and risk of atrophy is expected to be equal at around 10:30 for all genotypes (Figure 3).

The consequence of the differing interactions between genotypes for these three genes is that to design a strategy to reduce radiotherapy side-effects we need to directly consider patient genotypes rather than self-reported chronotypes. For example, considering just rs2087947 in *PER3*, we predict the optimum strategy would be to irradiate patients of C/C genotypes in the morning and the T/T patients in the afternoon. As each SNP has similar odds ratios, allele dosages can be combined into an unweighted PRS score. Considering these SNPs in a composite measure (Table 4) shows triple-homozygotes for either minor or major alleles can have radically different chances of developing atrophy depending on treatment time (OR 1.12 (95% CI 1.06-1.18); *P <* 0.0001 (LR)), illustrated in Figure 3d. An extreme example would be patients treated after wide area excision at 15:30 having probabilities of developing atrophy between 12% (0-23 95% CI) and 70% (60-80 95% CI) depending on genotypes. Based on allele frequencies in our European cohort (Table 2), we estimate ∼ 1,400 in 10,000 patients would be at the highest risk. Our models predict treating these patients six hours earlier reduces the risk from 70% to 33%.

Of the three SNPs shown to have alleles associated with late toxicity, the *CLOCK* gene SNP rs1801260 has been experimentally demonstrated to change the mRNA level, with a knock-on effect of higher expression of *PER2*, one of its transcriptional targets.^28^ The *PER3* SNP is in high linkage disequilibrium with the VNTR which alters by 20% the level of phosphorylation by CK1, the kinase which is responsible for translocating PER3 to the nucleus where it inhibits the central molecular clock by binding to CLOCK/BMAL1.^29^ There is no published functional evidence for the *RASD1* SNP rs11545787, but it is in the 3’ UTR of the gene and alters a CpG methylation site (Encode methylation track on UCSC genome browser).

These results agree in key ways with our previous analysis of the retrospective LeND cohort,^12^ but advance them through use of a prospective cohort, data from multiple clinical centres in different European countries, higher patient numbers and more sophisticated time analyses. The analyses both find an effect of time of treatment and the *PER3* gene on late radiotherapy side effects in the breast, and both find an interaction between time and genetic effects such that the optimal time of treatment is different according to genotype.

There is however a difference between the results in that the earlier study found that the late toxicity endpoint used, that of STAT score, was highest in patients treated in the morning, whereas here we report the peak for atrophy to be in the afternoon. STAT scores are composite endpoints that use the average of the Z-scores of individual measures.^30^ In Johnson et al.^12^ the STAT score included fibrosis, telangiectasia, atrophy and oedema, but was dominated by telangiectasia as the most common late effect. Breast atrophy as used in the present study is mainly due to tissue loss, with some retraction caused by fibrosis. Therefore, the two late toxicity end-points used were due to different tissues: vascular endothelium for the STAT score^12^; adipocytes, glandular tissue and fibroblasts in this study. There is abundant evidence that the circadian control of cell cycle varies between cell types,^31^ with peaks of mitosis at different times of day, providing one possible explanation for the apparent discrepancy between the two studies.

Our previous hypothesis was a direct effect of chronotype on radiation response, with variation in radiosensitivity over the cell cycle being the biological mechanism. Here, we find no effect from PRS for self-reported morningness, while a previous analysis on 268 Leicester REQUITE patients comparing responses to the Munich Chronotype Questionnaire^32^ found no association with either *PER3* or *CLOCK*.^33^ This is suggestive of a more direct role of circadian genes on cell cycle rather than overall chronotype, supported further by the finding that patients with alleles associated with ‘morningness’ actually have improved outcomes when treated later in the day. Long-term tissue loss is highly suggestive of increased stem cell loss following radiotherapy and a failure to maintain a tissue profile during the normal tissue turnover with time. We hypothesise increased stem cell loss is due to differential timing of the G2/M transition in patients with different genotypes.

We acknowledge this study has some limitations. Patients were treated on different radiotherapy protocols in each centre. Despite controlling for different factors and validation through use of random effect models, cryptic differences between the centres may cause false positive results. Time of radiotherapy fractions was not randomised but determined either by choice of the patients or clinic availability, with some patients having treatment fractions spread across a wide gamut of times. A more refined approach would be to conduct a trial in which patients are genotyped in advance and advised of the optimal time window for their treatment. We have only included candidate genes in this analysis, which is generally considered weaker than a non-hypothesis driven approach using genome-wide SNPs and inevitably misses some of the causative genetic variation. In future analyses we will include more SNPs, but given the experimental design there is insufficient power for a genome-wide analysis without a greatly expanded cohort size. Finally, our study is limited by its lack of generalizability to multi-ethnic populations. Individuals in this study are self-selected and all of European descent, with REQUITE patients from other ethnicities not included in the genetic analysis through use of principal components analysis for ancestry. It is possible that other ethnicities might have different allele frequencies for the polymorphisms used and therefore produce different results. Future studies could increase generalizability by assessing the effect of time and genotype in populations of different ethnicities and living at different latitudes.

The results reported in this paper support our earlier finding of an interactive effect of treatment time and genotype on late radiotherapy toxicity in breast cancer patients. An analysis of the REQUITE prostate cancer cohorts is ongoing, while in breast cancer, having carried out retrospective and prospective observational studies, the next stage should be a clinical trial in which the optimal time of treatment for different toxicity end-points is calculated in advance and used to guide treatment. Given the low cost and trivial nature of the intervention (guiding time of treatment) it should be possible to carry out a large multi-centre clinical trial to show that ‘chronoradiotherapy’ can reduce side effects. In breast cancer, having carried out retrospective and prospective observational studies, the next stage prior to changing European practice would be a clinical trial in which the optimal time of treatment for different toxicity end-points is calculated in advance and used to guide treatment. Patients would need to be randomized to non-chronomodulated or chronomodulated treatment groups. For the chronomodulated group SNP typing would identify the optimum time of treatment. Radiotherapy fractions would then be delivered at fixed times and the patients followed up for at least two years. Late toxicity would be the primary end-point, secondary end-points being acute toxicity, local tumour recurrence and patient quality-of-life.

## Contributors

CJT and RPS contributed to conceptualisation and design of the study. AJW was responsible for the conduct of the study. MAB, DA, CB, MB, RB, ACh, ACi, DdR, MCdS, AGC, SGE, KJ, RLB, GP, BSR, VR, AS, PSF, ES, BTV, RV, AV, LV recruited patients or collected data. EB, HSto and TW were patient representatives. AMD, LF, SK: SNP assay. AJW was responsible for the clinical database. AJW, CJT, TRat and EH contributed to the formal analysis. PS and JCC were responsible for data curation. RME and HSum were responsible for project management. AJW contributed to the methodology and visualisation of data. AJW, CJT and RPS wrote the original draft of the manuscript. TRat, JCC, CMW, PS, ES, DA, AVG, AMD, TRan, SGE contributed to review and editing of the manuscript. CMW led the REQUITE study. RPS and CJT supervised the study reported. AJW and PS verified the underlying data. All authors read and approved the final version of the manuscript

## Data sharing statement

Funding for the five year REQUITE project ended on 30th September 2018. REQUITE does not benefit financially from supplying data and/or samples to researchers but does make a charge to cover its costs and support continued maintenance of the database and biobank beyond the ending of the funding period. To facilitate this continued access to researchers, the REQUITE Steering Committee approved a tiered cost recovery model for access to data and/ or samples. Contact REQUITE (requite@manchester.ac.uk) for more information on pricing. All authors had access to all the data reported in the study. The senior authors had full access to all the data in the study and had the final responsibility for the decision to submit for publication.

## Declaration of interests

AC reports grants or contracts from Cancer Research UK, Prostate Cancer UK, UK Research Institute, National Institute of Health Research and Elekta AB; payments or honoraria from Bayer PLC, Janssen, AZ, ASTRO, ASCO, Roche and Merck. All other authors declare no potential conflicts of interest.

## Appendix Supplementary materials

Image, application 1

Image, application 2

Image, application 3

Image, application 4

Image, application 5

Image, application 6
