# Supplementary figures and images for: Treatment time and circadian genotype interact to influence radiotherapy side-effects. A prospective European validation study using the REQUITE cohort
Source: eBioMedicine. 2022 Sep 18;84:104269. doi: 10.1016/j.ebiom.2022.104269 (PMC9486558; doi:10.1016/j.ebiom.2022.104269)

**Supplementary figure 1:** ROC curves for a) erythema and b) atrophy deterioration models.

A


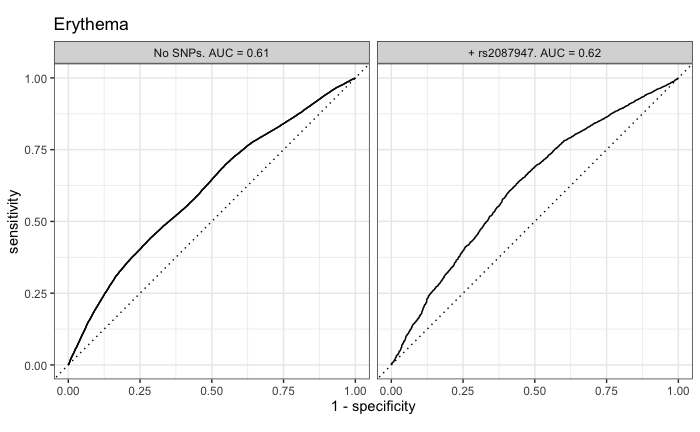


B


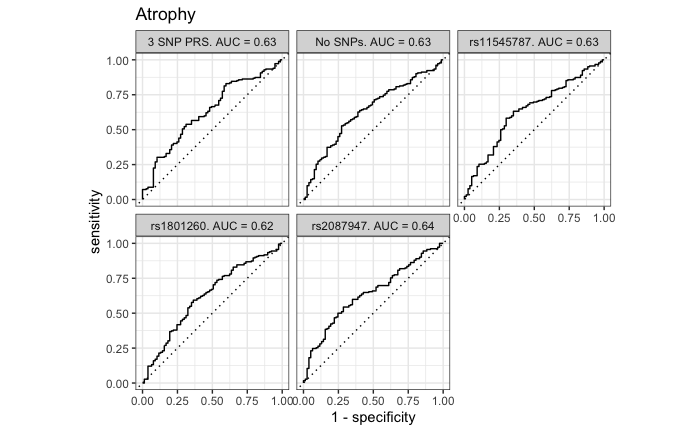

Supplement: Supplementary file 1 [file mmc1.docx]
