# Supplementary material for: Treatment time and circadian genotype interact to influence radiotherapy side-effects. A prospective European validation study using the REQUITE cohort
Source: eBioMedicine. 2022 Sep 18;84:104269. doi: 10.1016/j.ebiom.2022.104269 (PMC9486558; doi:10.1016/j.ebiom.2022.104269)
Supplement: Supplementary file 2 [file mmc2.docx]

**Supplementary table 1:** Univariate analysis of erythema (dichotomous) and atrophy (dichotomous) deterioration following radiotherapy.

|  | **Erythema (acute)** | | | | **Atrophy (late)** | | | |
| --- | --- | --- | --- | --- | --- | --- | --- | --- |
| **Characteristic** | **Overall, N = 1,712** | **0, N = 1,372** | **1, N = 340** | **p-value** | **Overall, N = 1,473** | **0, N = 959** | **1, N = 514** | **p-value** |
| **Site** |  |  |  | <0.001 |  |  |  | <0.001 |
| **Barcelona** | 193 (11%) | 110 (8.0%) | 83 (24%) |  | 188 (13%) | 149 (16%) | 39 (7.6%) |  |
| **Gent** | 295 (17%) | 262 (19%) | 33 (9.7%) |  | 237 (16%) | 143 (15%) | 94 (18%) |  |
| **Leicester** | 340 (20%) | 282 (21%) | 58 (17%) |  | 235 (16%) | 133 (14%) | 102 (20%) |  |
| **Leuven** | 248 (14%) | 205 (15%) | 43 (13%) |  | 241 (16%) | 122 (13%) | 119 (23%) |  |
| **Mannheim** | 36 (2.1%) | 31 (2.3%) | 5 (1.5%) |  | 32 (2.2%) | 23 (2.4%) | 9 (1.8%) |  |
| **Milan** | 101 (5.9%) | 60 (4.4%) | 41 (12%) |  | 89 (6.0%) | 59 (6.2%) | 30 (5.8%) |  |
| **Montpellier** | 399 (23%) | 333 (24%) | 66 (19%) |  | 367 (25%) | 289 (30%) | 78 (15%) |  |
| **Santiago** | 100 (5.8%) | 89 (6.5%) | 11 (3.2%) |  | 84 (5.7%) | 41 (4.3%) | 43 (8.4%) |  |
| **Age (years)** | 58 (11) | 59 (11) | 57 (11) | 0.015 | 58 (11) | 58 (11) | 59 (11) | 0.071 |
| **BMI (kg/m^2)** | 26.5 (5.6) | 26.3 (5.4) | 27.3 (6.1) | 0.016 | 26.2 (5.3) | 25.8 (5.2) | 27.1 (5.4) | <0.001 |
| **(no data)** | 17 | 12 | 5 |  | 14 | 6 | 8 |  |
| **Menopausal status** |  |  |  | 0.016 |  |  |  | 0.5 |
| **Pre** | 400 (24%) | 300 (22%) | 100 (30%) |  | 353 (24%) | 237 (25%) | 116 (23%) |  |
| **Peri** | 127 (7.5%) | 105 (7.8%) | 22 (6.5%) |  | 102 (7.1%) | 62 (6.6%) | 40 (7.9%) |  |
| **Post** | 1,156 (69%) | 941 (70%) | 215 (64%) |  | 989 (68%) | 640 (68%) | 349 (69%) |  |
| **(no data)** | 29 | 26 | 3 |  | 29 | 20 | 9 |  |
| **Diabetes** | 104 (6.1%) | 78 (5.7%) | 26 (7.6%) | 0.2 | 85 (5.8%) | 45 (4.7%) | 40 (7.8%) | 0.015 |
| **Smoking status** |  |  |  | 0.2 |  |  |  | 0.6 |
| **Never** | 936 (55%) | 746 (55%) | 190 (56%) |  | 825 (57%) | 528 (56%) | 297 (59%) |  |
| **Ex-smoker (before cancer diagnosis)** | 444 (26%) | 359 (27%) | 85 (25%) |  | 374 (26%) | 253 (27%) | 121 (24%) |  |
| **Ex-smoker (since cancer diagnosis)** | 73 (4.3%) | 52 (3.8%) | 21 (6.2%) |  | 57 (3.9%) | 38 (4.0%) | 19 (3.8%) |  |
| **Current smoker** | 238 (14%) | 197 (15%) | 41 (12%) |  | 198 (14%) | 132 (14%) | 66 (13%) |  |
| **(no data)** | 21 | 18 | 3 |  | 19 | 8 | 11 |  |
| **Surgery type** |  |  |  | <0.001 |  |  |  | <0.001 |
| **Segmentectomy / Quadrantectomy** | 835 (49%) | 628 (46%) | 207 (61%) |  | 762 (52%) | 553 (58%) | 209 (41%) |  |
| **Wide local excision** | 873 (51%) | 741 (54%) | 132 (39%) |  | 708 (48%) | 404 (42%) | 304 (59%) |  |
| **(no data)** | 4 | 3 | 1 |  | 3 | 2 | 1 |  |
| **Neoadjuvant chemotherapy (anthracycline)** | 136 (7.9%) | 99 (7.2%) | 37 (11%) | 0.025 | 127 (8.6%) | 79 (8.2%) | 48 (9.3%) | 0.5 |
| **Neoadjuvant chemotherapy (non-anthracycline)** | 150 (8.8%) | 106 (7.7%) | 44 (13%) | 0.002 | 141 (9.6%) | 90 (9.4%) | 51 (9.9%) | 0.7 |
| **Adjuvant chemotherapy (anthracycline)** | 351 (21%) | 273 (20%) | 78 (23%) | 0.2 | 306 (21%) | 192 (20%) | 114 (22%) | 0.3 |
| **(no data)** | 1 | 1 | 0 |  | 1 | 0 | 1 |  |
| **Adjuvant chemotherapy (non-anthracycline)** | 373 (22%) | 283 (21%) | 90 (26%) | 0.019 | 332 (23%) | 208 (22%) | 124 (24%) | 0.3 |
| **BED (acute toxicity) (Gy)** | 65 (11) | 65 (11) | 69 (10) | <0.001 | 66 (11) | 67 (11) | 66 (11) | 0.077 |
| **BED (late toxicity) (Gy)** | 94 (14) | 94 (14) | 97 (13) | <0.001 | 96 (14) | 96 (14) | 96 (14) | 0.5 |
| **Boost** | 1,150 (67%) | 898 (65%) | 252 (74%) | 0.002 | 1,050 (71%) | 671 (70%) | 379 (74%) | 0.13 |
| **Intensity-modulated radiation therapy (IMRT)** | 892 (52%) | 752 (55%) | 140 (41%) | <0.001 | 728 (49%) | 422 (44%) | 306 (60%) | <0.001 |
| **Mean treatment time (hours from local midnight)** | 12.62 (2.74) | 12.57 (2.71) | 12.82 (2.85) | 0.2 | 12.62 (2.77) | 12.57 (2.81) | 12.73 (2.70) | 0.2 |
| **Mean treatment time (hours from solar midnight)** | 11.35 (2.74) | 11.30 (2.72) | 11.53 (2.81) | 0.3 | 11.31 (2.78) | 11.23 (2.84) | 11.45 (2.66) | 0.11 |
| **Treatment time s.d. > 2 hrs** | 347 (20%) | 297 (22%) | 50 (15%) | 0.004 | 289 (20%) | 166 (17%) | 123 (24%) | 0.002 |
| **Baseline erythema** | 116 (6.8%) | 114 (8.3%) | 2 (0.6%) | <0.001 | 99 (6.8%) | 70 (7.4%) | 29 (5.7%) | 0.2 |
| **(no data)** |  |  |  |  | 9 | 8 | 1 |  |
| **Baseline atrophy** | 76 (5.2%) | 59 (5.0%) | 17 (5.9%) | 0.5 | 76 (5.2%) | 72 (7.5%) | 4 (0.8%) | <0.001 |
| **(no data)** | 248 | 196 | 52 |  |  |  |  |  |
| **Atrophy deterioration** | 513 (35%) | 410 (35%) | 103 (36%) | 0.8 |  |  |  |  |
| **(no data)** | 248 | 196 | 52 |  |  |  |  |  |
| **Erythema deterioration** |  |  |  |  | 288 (20%) | 185 (19%) | 103 (20%) | 0.8 |
| **(no data)** |  |  |  |  | 9 | 8 | 1 |  |
| **n (%); Mean (SD)** | | | | | | | | |
| **P-values: Pearson's Chi-squared test; Wilcoxon rank sum test** | | | | | | | | |
