# Supplementary material for: Treatment time and circadian genotype interact to influence radiotherapy side-effects. A prospective European validation study using the REQUITE cohort
Source: eBioMedicine. 2022 Sep 18;84:104269. doi: 10.1016/j.ebiom.2022.104269 (PMC9486558; doi:10.1016/j.ebiom.2022.104269)
Supplement: Supplementary file 3 [file mmc3.docx]

**Supplementary table 2** – Logistic regression models for late atrophy (dichotomous), mixed effects with treatment centre (site) as a random intercept.

| **Characteristic** | **No SNPs** | | | **rs2087947 (PER3)** | | | **rs1801260 (CLOCK)** | | | **rs11545787 (RASD1)** | | | **3 SNP PRS** | | |
| --- | --- | --- | --- | --- | --- | --- | --- | --- | --- | --- | --- | --- | --- | --- | --- |
|  | OR*^1^* | 95% CI*^1^* | p-value | OR*^1^* | 95% CI*^1^* | p-value | OR*^1^* | 95% CI*^1^* | p-value | OR*^1^* | 95% CI*^1^* | p-value | OR*^1^* | 95% CI*^1^* | p-value |
| BMI (kg/m^2) | 1.05 | 1.03, 1.08 | **<0.001** | 1.05 | 1.03, 1.08 | **<0.001** | 1.05 | 1.03, 1.08 | **<0.001** | 1.05 | 1.03, 1.08 | **<0.001** | 1.06 | 1.03, 1.08 | **<0.001** |
| BED (late) / Gy ^2^ | 1.02 | 1.01, 1.03 | **0.005** | 1.02 | 1.01, 1.03 | **0.004** | 1.02 | 1.01, 1.03 | **0.006** | 1.02 | 1.01, 1.03 | **0.004** | 1.02 | 1.01, 1.03 | **0.004** |
| Surgery: wide local excision  vs. segmentectomy /  quadrantectomy | 1.63 | 0.99, 2.67 | 0.054 | 1.57 | 0.95, 2.60 | 0.080 | 1.58 | 0.96, 2.60 | 0.073 | 1.60 | 0.96, 2.65 | 0.069 | 1.51 | 0.90, 2.53 | 0.12 |
| Mean treatment time  from solar 1530 (hrs) | 0.93 | 0.88, 0.98 | **0.010** | 0.88 | 0.82, 0.95 | **<0.001** | 0.88 | 0.82, 0.95 | **<0.001** | 0.86 | 0.80, 0.93 | **<0.001** | 0.78 | 0.71, 0.87 | **<0.001** |
| SNP (dosage, allele 2 or unweighted PRS^3^) |  |  |  | 0.63 | 0.41, 0.95 | **0.027** | 0.66 | 0.43, 1.01 | 0.057 | 0.52 | 0.31, 0.85 | **0.009** | 0.62 | 0.48, 0.80 | **<0.001** |
| SNP * treatment time |  |  |  | 1.10 | 1.01, 1.19 | **0.028** | 1.10 | 1.01, 1.20 | **0.029** | 1.17 | 1.05, 1.29 | **0.003** | 1.11 | 1.05, 1.16 | **<0.001** |
|  |  |  |  |  |  |  |  |  |  |  |  |  |  |  |  |
| Site.sd__(Intercept) | 0.40 |  |  | 0.41 |  |  | 0.39 |  |  | 0.42 |  |  | 0.42 |  |  |
| N | 1109 |  |  | 1054 |  |  | 1054 |  |  | 1054 |  |  | 1052 |  |  |
| AIC | 1,282 |  |  | 1,281 |  |  | 1,281 |  |  | 1,277 |  |  | 1,269 |  |  |
| BIC | 1,312 |  |  | 1,320 |  |  | 1,321 |  |  | 1,317 |  |  | 1,309 |  |  |
| Atrophy (0/1) - change of >= 1 grade atrophy. Sample includes patients with baseline atrophy up to and including Grade 1 *^1^*OR = Odds Ratio, CI = Confidence Interval  ^2^ Biological effective dose  ^3^ Unweighted PRS score is generated by summing the dosage of allele 2 (0-2) for each of the circadian SNPs rs1801260 (*CLOCK*), rs2087947 (*PER3*) and rs11545787 (*RASD1*) separately analysed. As each SNP shows all overall decrease in atrophy for allele 2 when considered independently of treatment time, they can be simply combined into a single risk score (0-6), where the ‘risk’ is overall reduced atrophy. | | | | | | | | | | | | | | | |
