# Supplementary material for: Treatment time and circadian genotype interact to influence radiotherapy side-effects. A prospective European validation study using the REQUITE cohort
Source: eBioMedicine. 2022 Sep 18;84:104269. doi: 10.1016/j.ebiom.2022.104269 (PMC9486558; doi:10.1016/j.ebiom.2022.104269)
Supplement: Supplementary file 4 [file mmc4.docx]

**Supplementary figure 1:** ROC curves for a) erythema and b) atrophy deterioration models

**Supplementary table 1:** Univariate analysis of erythema (dichotomous) and atrophy (dichotomous) deterioration following radiotherapy.

**Supplementary table 2** – Logistic regression models for late atrophy (dichotomous), mixed effects with treatment centre (site) as a random intercept.
