# Supplementary material for: Treatment time and circadian genotype interact to influence radiotherapy side-effects. A prospective European validation study using the REQUITE cohort
Source: eBioMedicine. 2022 Sep 18;84:104269. doi: 10.1016/j.ebiom.2022.104269 (PMC9486558; doi:10.1016/j.ebiom.2022.104269)
Supplement: Supplementary file 5 [file mmc5.docx]

| 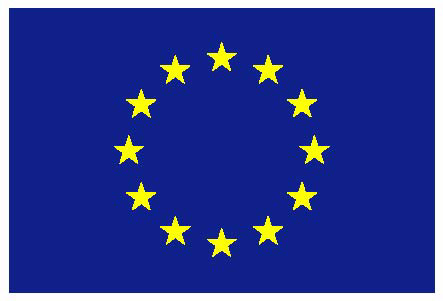 |  | 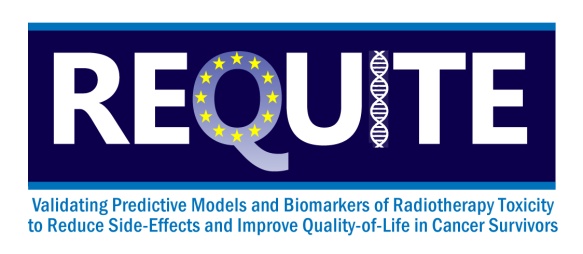 |
| --- | --- | --- |

**REQUITE Study Protocol - UK**

**Validating predictive models and biomarkers of radiotherapy toxicity to reduce side-effects and improve quality-of-life in cancer survivors**

**Protocol Version 6.0, 31/01/2019**

The REQUITE project is financially supported by the 7th Framework Programme

of the European Commission (Grant number 601826)

Principal Investigator (Print name) Signature Date

Prof. Dr. Jenny Chang-Claude

Observational Study Lead Signature Date

Prof. Catharine West

REQUITE Co-ordinator Signature Date

**I confirm that I have read and understood REQUITE protocol v6.0, dated 31/01/2019. I agree to work in accordance with the procedures laid out in this document and to the research principles that have their basis in ICH GCP. I will ensure that all staff working on REQUITE at my centre are aware of their responsibilities and are compliant with the study protocol.**

**LIST OF PARTICIPANTS**

**REQUITE Co-ordinator**

**Prof. Catharine West**

University of Manchester (UNIMAN)

Institute of Cancer Sciences, Christie Hospital, Wilmslow Road, Manchester, M20 4BX, UK

Tel: +44 (0)161 446 8275

Fax: +44 (0)161 446 8111

Email: [Catharine.West@manchester.ac.uk](mailto:Catharine.West@manchester.ac.uk)

**Multi-Centre Observational Study Lead**

**Prof. Dr. Jenny Chang-Claude**German Cancer Research Center (DKFZ)

Division of Cancer Epidemiology, Unit of Genetic Epidemiology, Im Neuenheimer Feld 581,

69120 Heidelberg, GERMANY

Tel: +49 6221 422373
Fax: +49 6221 422203
Email: [j.chang-claude@dkfz-heidelberg.de](mailto:j.chang-claude@dkfz-heidelberg.de)

**Biobank Lead**

**Dr. Antony Payton**

University of Manchester (UNIMAN)

Centre for Integrated Genomic Medical Research, Stopford Building, Oxford Road, Manchester, M13 9PT, UK

Tel: +44 (0)161 275 5714

Email: [tony.payton@manchester.ac.uk](mailto:tony.payton@manchester.ac.uk)

**Clinical Leads**

**Prof. Dr. Frederik Wenz**

Department of Radiation Oncology, Medical Faculty Mannheim, University of Heidelberg

Theodor-Kutzer-Ufer 1-3, 68167 Mannheim, Germany

Tel.: +49 (0) 621 383 3530

Fax: +49 (0) 621 383 3493

Email: [Frederik.Wenz@medma.uni-heidelberg.de](mailto:Frederik.Wenz@medma.uni-heidelberg.de)

**Prof. Ananya Choudhury**

The Christie NHS Foundation Trust (CNFT)

The Christie, Wilmslow Road, Manchester, M20 4BX, UK

Tel: +44 (0)161 918 7475

Fax: +44 (0)161 446 8111

Email: ananya.choudhury@christie.nhs.uk

**Dr. Ana Vega**

Fundación Pública Galega de Medicina Xenómica (FPGMX)

Hosp. Clinico Univ. Edificio Consultas Planta Menos 2, 15706 Santiago, Spain

Tel: +34 981 95 14 91

Fax: +34 981 95 14 73

Email: [ana.vega@usc.es](mailto:ana.vega@usc.es)

**Dr. Riccardo Valdagni**

Fondazione IRCCS Istituto Nazionale dei Tumori (INT)

Via G. Venezian 1 – 20133, Milano, Italy

Tel: +39 02 23903034

Fax: +39 02 23903015

Email: [riccardo.valdagni@istitutotumori.mi.it](mailto:riccardo.valdagni@istitutotumori.mi.it)

**Dr Maarten Lambrecht**

Katholieke Universiteit **Leuven** (KULEUVEN)

Leuven Cancer Institute, Campus Gasthuisberg, Herestraat 49, 3000 Leuven, Belgium

Tel: +32 16 34 69 02

Fax: +32 16 34 76 23

Email: maarten.lambrecht@uzleuven.be

**Dr. Barry Rosenstein**

Icahn School of Medicine at Mount Sinai (MSSM)

1 Gustave Levy Place, Box 1236, New York, NY 10029, USA

Tel: +01 212 241 9408

Fax: +01 212 996 8927

Email: [Barry.Rosenstein@exchange.mssm.edu](mailto:Barry.Rosenstein@exchange.mssm.edu)

**Prof. Liv Veldeman**

Ghent University Hospital

Department of Radiation Oncology, De Pintelaan 185, 9000 Ghent, Belgium

Tel: +32 9 332 58 19

Fax: +32 9 332 30 40

Email: [liv.veldeman@uzgent.be](mailto:liv.veldeman@uzgent.be)

**Prof. Paul Symonds**

University of Leicester (ULEIC)

University of Leicester, University Road, Leicester LE1 7RH, UK

Tel: +44 (0)116 258 6294

Fax: +44 (0)116 258 7597

Email: [paul.symonds@uhl-tr.nhs.uk](mailto:paul.symonds@uhl-tr.nhs.uk)

**Prof. David Azria**

l'Institut régional du Cancer Montpellier (ICM)

Rue Croix verte, Department of Radiation Oncology, ICM, Montpellier 34298, France

Tel: +33 467 61 8579

Fax: +33 467 61 31 35

Email: [David.Azria@icm.unicancer.fr](mailto:David.Azria@icm.unicancer.fr)

**Prof. Dr. Dirk De Ruysscher**

Maastricht Radiation Oncology (MAASTRO)
University Hospital Maastricht, Postbox 5800, 6202 AZ Maastricht, The Netherlands

Tel: +31 (0) 88 44 55754

Fax: +31 (0) 88 44 55776

Email: dirk.deruysscher@maastro.nl

**Genotyping Lead**

**Dr. Alison Dunning**

University of Cambridge (UCAMB)

Centre for Cancer Genetic Epidemiology, University of Cambridge, Strangeways Research Laboratory, Worts Causeway, Cambridge, CB1 8RN, UK

Tel: +44 (0)1233 740683

Fax: +44 (0)1223 740147

Email: [amd24@medschl.cam.ac.uk](mailto:amd24@medschl.cam.ac.uk)

**Biomarker Lead**

**Dr. Chris Talbot**

University of Leicester (ULEIC)

University of Leicester, University Road, Leicester LE1 7RH, UK

Tel: +44 (0)116 252 3433

Fax: +44 (0)116 252 3378

Email: [cjt14@leicester.ac.uk](mailto:cjt14@leicester.ac.uk)

**Database Lead**

**Prof. Anthony Brookes**

University of Leicester (ULEIC)

Department of Genetics, University of Leicester, University Road, Leicester, LE1 7RH, UK

Tel: +44 (0)116 2523401

Fax: +44 (0)116 2523378

Email: [ajb97@leicester.ac.uk](mailto:ajb97@leicester.ac.uk)

**Clinical Models Lead**

**Dr. Tiziana Rancati**

Fondazione IRCCS Istituto Nazionale dei Tumori (INT)

Via G. Venezian 1 – 20133, Milano, Italy

Tel: +39 02 23903786

Fax: +39 02 23903015

Email: Tiziana.Rancati@istitutotumori.mi.it

**Statistics Lead**

**Prof. Søren Bentzen**

University of Wisconsin-Madison

600 Highland Avenue
Madison, WI 53792-4675, USA
Tel: +01 608 265 8572
Fax: +01 608 263 9947

Email: [bentzen@humonc.wisc.edu](mailto:bentzen@humonc.wisc.edu)

**REQUITE Project Manager**

**Ms. Rebecca Elliott**

University of Manchester (UNIMAN)

Institute of Cancer Sciences, Christie Hospital, Wilmslow Road, Manchester, M20 4BX, UK

Tel: +44 (0)161 446 3045

Fax: +44 (0)161 446 8111

Email: [rebecca.m.elliott@manchester.ac.uk](mailto:rebecca.m.elliott@manchester.ac.uk)

**Observational Study Manager**

**Dr. Petra Seibold / Dr Akke Botma**

German Cancer Research Center (DKFZ)

Division of Cancer Epidemiology, Unit of Genetic Epidemiology, Im Neuenheimer Feld 581,

69120 Heidelberg, GERMANY

Tel: +49 6221 422208
Fax: +49 6221 422203

Email: [REQUITE@dkfz.de](mailto:REQUITE@dkfz.de)

**REQUITE Data Manager**

**Ms. Anusha Appanvel**

German Cancer Research Center (DKFZ)

Division of Cancer Epidemiology, Unit of Genetic Epidemiology, Im Neuenheimer Feld 581,

69120 Heidelberg, GERMANY

Tel: +49 6221 423181
Fax: +49 6221 422203

Email: [a.appanvel@dkfz-heidelberg.de](mailto:a.appanvel@dkfz-heidelberg.de)

**ABBREVIATIONS**

| CIGMR | Centre for Integrated Genomic Medical Research |
| --- | --- |
| CNFT | The Christie NHS Foundation Trust, UK |
| CNV | Copy Number Variation |
| CRF | Case Record Form |
| CTCAE | Common Terminology Criteria for Adverse Events |
| DKFZ | German Cancer Research Center, Germany |
| FPGMX | Fundación Pública Galega de Medicina Xenómica, Spain |
| GCP | Good Clinical Practice |
| ICM | l'Institut régional du Cancer Montpellier, France |
| INT | Fondazione IRCCS Istituto Nazionale dei Tumori, Italy |
| KULEUVEN | Katholieke Universiteit Leuven, Belgium |
| LIMS | Laboratory Information Management System |
| MAASTRO | Maastricht Radiation Oncology, The Netherlands |
| MSSM | Icahn School of Medicine at Mount Sinai, USA |
| NTCP | Normal Tissue Complication Probability |
| PI | Principal Investigator |
| QoL | Quality of Life |
| SMG | Study Management Group |
| SNP | Single Nucleotide Polymorphism |
| STAT | Standardised Total Average Toxicity |
| UCAMB | University of Cambridge, UK |
| UGENT | Universiteit Gent, Belgium |
| ULEIC | University of Leicester, UK |
| UMM | Universitaetsmedizin Mannheim, part of University of Heidelberg, Germany |
| UMONT | University of Montpellier, France |
| UNIMAN | University of Manchester, UK |

**TABLE OF CONTENTS**

[1.0 STUDY SUMMARY 10](#_Toc536792493)

[1.1 Study Schema 12](#_Toc536792494)

[2.0 BACKGROUND 13](#_Toc536792496)

[2.1 Cancer survivorship 13](#_Toc536792497)

[2.2 Long-term side-effects of radiotherapy impact on quality-of-life 13](#_Toc536792498)

[2.3 Importance of genetics and biomarkers of radiosensitivity 13](#_Toc536792499)

[2.4 Rationale for the study 15](#_Toc536792500)

[3.0 STUDY OBJECTIVES 16](#_Toc536792501)

[3.1 Overall Design 16](#_Toc536792502)

[3.2 Primary/ Secondary Objectives 16](#_Toc536792503)

[3.3 Study Endpoints 16](#_Toc536792504)

[3.4 Data Collection 17](#_Toc536792505)

[3.5 Sample Collection 18](#_Toc536792506)

[3.6 Study Duration 18](#_Toc536792507)

[4.0 SELECTION OF STUDY PATIENTS 19](#_Toc536792508)

[4.1 Inclusion Criteria 19](#_Toc536792509)

[4.2 Exclusion Criteria 19](#_Toc536792510)

[5.0 RECRUITMENT OF PATIENTS 21](#_Toc536792511)

[5.1 Number of patients required 21](#_Toc536792512)

[5.2 Identifying patients 21](#_Toc536792513)

[5.3 Consenting patients 21](#_Toc536792514)

[6.0 STUDY METHODOLOGY 22](#_Toc536792515)

[6.1 Patient Registration 22](#_Toc536792516)

[6.2 Sample Collection and Tracking 22](#_Toc536792517)

[6.3 Data Collection 24](#_Toc536792518)

[6.4 Data Handling 26](#_Toc536792519)

[6.5 Radiotherapy 26](#_Toc536792520)

[6.6 Biomarker Assays 27](#_Toc536792521)

[6.6.1 Genotyping 27](#_Toc536792522)

[6.6.2 Live cell apoptosis assays 27](#_Toc536792523)

[6.6.3 DNA damage and repair assays 28](#_Toc536792524)

[7.0 STATISTICAL PLANS AND DATA ANALYSIS 29](#_Toc536792525)

[7.1 Power calculations 29](#_Toc536792526)

[7.2 Statistical Data Analysis 29](#_Toc536792527)

[8.0 COMPLIANCE, DATA PROTECTION AND DATA SHARING 31](#_Toc536792528)

[8.1 Compliance 31](#_Toc536792529)

[8.2 Data Protection 31](#_Toc536792530)

[8.3 Data Sharing 31](#_Toc536792531)

[9.0 ETHICAL AND REGULATORY REQUIREMENTS 31](#_Toc536792532)

[9.1 Sponsor and Indemnity 32](#_Toc536792533)

[9.2 Informed Consent 32](#_Toc536792534)

[9.3 Patient Withdrawal 33](#_Toc536792535)

[10.0 STUDY MANAGEMENT AND OVERSIGHT ARRANGEMENTS 34](#_Toc536792536)

[10.1 Study Management Group 34](#_Toc536792537)

[10.2 Study Data 34](#_Toc536792538)

[10.3 Study Monitoring 34](#_Toc536792539)

[11.0 PUBLICATION 35](#_Toc536792540)

[12.0 SCHEDULE OF ASSESSMENTS 36](#_Toc536792541)

[12.1 Breast Patients 36](#_Toc536792542)

[12.2 Prostate Patients 37](#_Toc536792543)

[12.3 Lung Patients 38](#_Toc536792544)

[13.0 SUB-STUDY 1: REQUITE-AB 39](#_Toc536792545)

[13.1 Summary 39](#_Toc536792546)

[13.2 Background 41](#_Toc536792547)

[13.2.1 Radiotherapy in breast cancer 41](#_Toc536792548)

[13.2.2 Acute radiotherapy toxicity – impact on treatment outcomes 41](#_Toc536792549)

[13.2.3 Genetic markers of acute radiotherapy toxicity 42](#_Toc536792550)

[13.2.4 Rationale for the study 43](#_Toc536792551)

[13.3 Sub-study research plan 43](#_Toc536792552)

[13.3.1 Study objectives and design 43](#_Toc536792553)

[13.3.2 Patient selection and recruitment 43](#_Toc536792554)

[13.3.3 Study methodology and data analysis 43](#_Toc536792555)

[13.4 Sub-study research arrangements 44](#_Toc536792556)

[13.4.1 Compliance and data protection 44](#_Toc536792557)

[13.4.2 Ethical and regulatory requirements 44](#_Toc536792558)

[13.4.3 Management and oversight arrangements 44](#_Toc536792559)

[13.4.4 Sub-study publication 44](#_Toc536792560)

[14.0 SUB-STUDY 2: REQUITE-AB-QOL 46](#_Toc536792561)

[14.1 Summary 46](#_Toc536792562)

[14.2 Background 47](#_Toc536792563)

[14.2.1 Radiotherapy impact on quality of life (QoL) 47](#_Toc536792564)

[14.2.2 Rationale for the study 47](#_Toc536792565)

[14.3 Sub-study research plan 47](#_Toc536792566)

[14.3.1 Study objectives and design 47](#_Toc536792567)

[14.3.2 Patient selection and recruitment 48](#_Toc536792568)

[14.3.3 Study methodology and data analysis 48](#_Toc536792569)

[14.4 Sub-study research arrangements 49](#_Toc536792570)

[14.4.1 Compliance and data protection 49](#_Toc536792571)

[14.4.2 Ethical and regulatory requirements 50](#_Toc536792572)

[14.4.3 Management and oversight arrangements 50](#_Toc536792573)

[14.4.4 Sub-study publication 51](#_Toc536792574)

[15.0 SUB-STUDY 3: REQUITE–CHRONO 52](#_Toc536792575)

[15.1 Summary 52](#_Toc536792576)

[15.2.1 Circadian Rhythm 53](#_Toc536792577)

[15.2.2 Rationale for the study 53](#_Toc536792578)

[15.3 Sub-study research plan 53](#_Toc536792579)

[15.3.1 Study objectives and design 53](#_Toc536792580)

[15.3.2 Patient selection and recruitment 54](#_Toc536792581)

[15.3.3 Study methodology and data analysis 54](#_Toc536792582)

[15.4 Sub-study research arrangements 54](#_Toc536792583)

[15.4.1 Compliance and data protection 54](#_Toc536792584)

[15.4.2 Ethical and regulatory requirements 54](#_Toc536792585)

[15.4.3 Management and oversight arrangements 55](#_Toc536792586)

[15.4.4 Sub-study publication 56](#_Toc536792587)

[16.0 SUB-STUDY 4: REQUITE-BP (Breast Pain) Sub-study 57](#_Toc536792588)

[16.1 Summary 57](#_Toc536792589)

[16.2 Background 58](#_Toc536792590)

[16.2.1 Chronic breast-pain following breast cancer treatment 58](#_Toc536792591)

[16.2.2 Rationale for the study 60](#_Toc536792592)

[16.3 Sub-study research plan 61](#_Toc536792593)

[16.3.1 Study objectives and design 61](#_Toc536792594)

[16.3.2 Patient selection and recruitment 61](#_Toc536792595)

[16.3.3 Study methodology and data analysis 61](#_Toc536792596)

[16.4 Sub-study-research arrangements 62](#_Toc536792597)

[16.4.1 Compliance and data protection 62](#_Toc536792598)

[16.4.2 Ethical and regulatory requirements 62](#_Toc536792599)

[16.4.3 Management and oversight of arrangements 63](#_Toc536792600)

[16.4.4 Sub-Study publication 63](#_Toc536792601)

# 1.0 STUDY SUMMARY

| **TITLE** | REQUITE: Validating predictive models and biomarkers of radiotherapy toxicity to reduce side-effects and improve quality-of-life in cancer survivors |
| --- | --- |
| **STUDY DESIGN** | Observational/ cohort study |
| **TARGET DISEASE** | Cancer of the breast, prostate or lung |
| **PRIMARY STUDY OBJECTIVE** | To establish a prospective cohort of patients undergoing radiotherapy for breast, prostate or lung cancer according to local regimens and collecting standardised radiotherapy toxicity data, non-genetic risk factor data and samples for biomarker assays for the study of determinants of radiotherapy side-effects. |
| **SECONDARY STUDY OBJECTIVES** | To establish a comprehensive centralised database and sample collection as a resource for the prospective evaluation and validation of clinical models incorporating biomarker data to identify before treatment those cancer patients who are at risk of developing long term side-effects from radiotherapy. |
| **PRIMARY ENDPOINTS** | - Change in breast appearance at 24 months following start of radiotherapy (breast) - Rectal bleeding at 24 months following start of radiotherapy (prostate) - Dyspnea/ breathlessness at 12 months following start of radiotherapy (lung) |
| **SECONDARY ENDPOINTS** | - Other toxicity endpoints including but not limited to: fibrosis, induration and vascular changes (breast); rectal incontinence, urinary toxicity and erectile dysfunction (prostate); dysphagia and oesophagitis (lung) - Quality of life - Maximum grade of toxicity during follow-up period |
| **STUDY POPULATION** | Male or female patients over 18 years of age with primary cancer of the breast, prostate or lung who are going to receive planned radical radiotherapy or adjuvant radiotherapy after breast conserving surgery or prostatectomy. |
| **RECRUITMENT TARGET** | 5,300 cancer patients over a 24 month accrual period |
| **FOLLOW UP VISITS** | Toxicity will be assessed and documented using REQUITE toxicity questionnaires based on the CTCAE v4.0 and EORTC Quality of Life at the following time points. Some site specific questionnaires will be used.   - Baseline assessed prior to radiotherapy (all) - End of radiotherapy (breast and prostate); or first follow-up visit following implantation for prostate brachytherapy patients - 3 months from start of radiotherapy (lung) and breast (University of Leicester only) - 6 months from start of radiotherapy (lung) - 12 months from start of radiotherapy (all) - Minimum 24 months from start of radiotherapy (all)   The follow-up period can be up to 5 years. Follow-up beyond the minimum of 24 months is permissible and encouraged where it’s possible as part of routine clinical care. |
| **STUDY DURATION** | - Minimum 24 months but actual follow-up period will depend on time of consent and duration of routine follow-up available at the recruiting centre (see follow-up visits) - Minimum 4 years overall (24 months recruitment period, 24 months minimum follow-up period) |
| **SAMPLE COLLECTION** | Pre-treatment blood samples will be collected for downstream analyses:   - Sample A: One 10ml EDTA sample for DNA extraction to investigate genetic variation as a predictor of radiotherapy toxicity (n~5,300). - Depending on the recruiting site further samples can include:   - Sample B: A 2.5ml PAXgene sample for future RNA collection and storage (n~3,500)   - Sample C: A 10ml Lithium Heparin sample for live cell apoptosis assays (n~1,800) and further DNA damage assays. Repeat LiH samples will be collected from a sub-set of patients at University of Leicester only (n=200 max). |
| **POWER CALCULATIONS** | It is estimated that 2-year toxicity data (1-year for lung) will be available for 75% of enrolled patients. Based on effect sizes observed for genetic associations with radiation toxicity, a power calculation for the genetic assays shows that 1,575 patients for breast and prostate cancer has 80% power to detect a RR of >1.56 for at least grade 2 toxicity (α = 5 x 10^-5^ for 1000 SNPs, allele freq = 0.25, toxicity rate = 20%), or with more stringent criteria a 90% power to detect a RR >1.66 (α = 1 x 10^-5^, allele freq = 0.25, toxicity rate = 20%). For 825 patients with lung cancer the detectable RR would be 1.78 and 1.92. |

## 1.1 Study Schema

#

# 2.0 BACKGROUND

## 2.1 Cancer survivorship

In December 2011 the International Agency for Research on Cancer released its global cancer estimates for 2008^[[1]](#footnote-1)^. The worldwide number of cancer survivors within five years of diagnosis was estimated to be ~28.7 million for 2008. Cancers of the breast (over 5 million survivors) and prostate (over 3 million survivors) had the highest prevalence in females (in 145 countries) and males (in 111 countries), respectively. Lung cancer has the highest overall prevalence globally and potentially curative treatment of lung cancer is increasing 2-year survival rates to ~75%^[[2]](#footnote-2)^.

With increasing life expectancies and improvements in diagnosis and treatment, the number of cancer patients and survivors is expected to continue to rise. As the illness increasingly becomes a chronic disease, cancer patients’ quality-of-life needs to be addressed in a systematic manner in order to enhance their participation in society, including the workplace.

## 2.2 Long-term side-effects of radiotherapy impact on quality-of-life

Radiotherapy represents the most effective non-surgical modality in the curative treatment of cancer. Around a half of all cancer patients receive radiotherapy at some point in their treatment and nearly a half of all cancer survivors underwent radiotherapy as part of their care^[[3]](#footnote-3)^.

Yet, many patients receiving potentially curative radiotherapy will experience toxicity due to the unavoidable irradiation of surrounding healthy tissue. The toxicity varies in severity from minor to severe and in duration from weeks to a lifetime. Around 3-5% of people suffer with severe long-term side-effects but more experience moderate toxicity such as poor breast cosmetic outcome, incontinence, erectile dysfunction, chronic breathlessness, and chronic pain. Moderate toxicity, such as a poor cosmetic outcome following breast cancer treatment, can have a marked effect on subsequent psychological outcome^[[4]](#footnote-4)^. The long-term side-effects of radiotherapy have been shown to impair quality-of-life in cancer survivors^[[5]](#footnote-5),^^[[6]](#footnote-6),^^[[7]](#footnote-7)^. The ability to predict those patients likely to develop toxicity could potentially enable individual dose prescription to be made, which should improve survival and decrease morbidity.

## 2.3 Importance of genetics and biomarkers of radiosensitivity

Individual variation in toxicity was established from the early days of radiotherapy but it is only in the past 20 years that the role of genetics is widely recognised as being important. There is interest in measuring a patient’s radiosensitivity to predict their likelihood of developing long-term side-effects following radiotherapy. The first report that an individual with ataxia telangiectasia suffered severe toxicity following radiotherapy showed that fibroblasts cultured from a skin sample were approximately three times more radiosensitive than cells from normal individuals. A number of subsequent reports showed that individuals with severe life-threatening radiotherapy toxicity had radiosensitive fibroblasts. These severe reactors are extremely rare and up until the 1980s it was thought that radiosensitivity varied little between most individuals. However, during the 1980s there was increasing evidence for a spectrum of radiosensitivity within the population. This evidence led to the initiation of studies measuring the radiosensitivity of cells from cancer patients to investigate whether it was possible to predict their risk of developing side-effects.

There is interest in using rapid cell based functional assays for measuring radiosensitivity. Of particular relevance is a flow cytometry method for assessing radiation-induced apoptosis. High toxicity following radiotherapy has been linked with low levels of radiation-induced apoptosis in several independent studies. A number of assays were studied in the past and the apoptosis assay stands out because the findings have been replicated in several independent laboratories^[[8]](#footnote-8),^^[[9]](#footnote-9),^^[[10]](#footnote-10),^^[[11]](#footnote-11),^^[[12]](#footnote-12),^^[[13]](#footnote-13),^^[[14]](#footnote-14)^. The apoptosis assay provides an assessment of the radiosensitivity of a patient at the time of radiotherapy, i.e. incorporating both genetic and epigenetic effects. This work will establish whether the apoptosis assay can be validated within a prospective multi-centre study using a standardised protocol. To do this, the assay will be standardised and quality assurance and control methods set up for cross laboratory analyses. The latter are required for clinical testing. If the assay can be neither standardised nor validated, then definitive evidence will be obtained that further studies using the assay are not warranted.

There is now good evidence for the heritability of radiosensitivity as a trait and growing interest in identifying the genetic variants associated with increased sensitivity to radiation^[[15]](#footnote-15)^. Most studies in this area attempt to identify the SNPs (i.e. common genetic variation) associated with radiosensitivity and there are a large number of candidate genes that have been explored^53^. As for other traits and diseases, radiosensitivity is considered to be an inherited trait that is determined by common variation in a large number of genes each conferring small effects on the phenotype. Rare mutations in several DNA damage response genes (e.g. *ATM*) are known to confer large effects but these are associated with genetic syndromes and their rarity means they have limited use for the general population who undergo radiotherapy. Genetic association studies provided evidence linking SNPs in various candidate genes with radiotherapy side-effects, but the associations have mainly not been replicated^[[16]](#footnote-16),^^[[17]](#footnote-17)^. This situation is being resolved through meta-analyses organised through the Radiogenomics Consortium. These are excluding some genes such as *TGFB1* in breast cancer patients^[[18]](#footnote-18)^, and providing evidence for others e.g. *TNF^[[19]](#footnote-19)^*. Several genome wide association studies (GWAS), underway or about to be published, are starting to provide a growing list of SNPs with evidence for predicting radiotherapy side-effects in different cancers^[[20]](#footnote-20),^^[[21]](#footnote-21)^.

Emerging evidence suggests that the SNPs will need targeting for specific endpoints. Several studies highlighted how information from SNP profiling can be incorporated into clinical models being explored to predict risk of radiotherapy toxicity^[[22]](#footnote-22),^^[[23]](#footnote-23)^. With the increasing international co-operation, recognition for the need to collect harmonised data and acceptance of the importance of validation, the time is right for a project that validates the clinical models, alone and incorporating biomarker data.

REQUITE provides the international cooperation, data and samples to validate models in several cancers, prior to evaluation in interventional clinical trials. It is recognised that work in other fields highlights the challenges in linking genotype with phenotype, but the REQUITE focus on validation rather than discovery will enable definitive findings regarding the importance of individual genetic variants that are starting to emerge from GWAS meta-analyses.

## 2.4 Rationale for the study

In recent years predictive models have been developed that attempt to identify before the start of treatment patients at risk of long-term side-effects. These emerging models require systematic validation in a multi-centre collaborative setting. There are an increasing number of datasets available for validation but they are variable in terms of the data collected.

This multi-centre observational study will be the largest study of its kind collecting blood samples and standardised data longitudinally from 5,300 cancer patients. It will enable validation of models that predict a patient’s risk of developing long-term side-effects following radiotherapy. It will be a unique (eventually widely accessible) resource for studying the relationships between side-effect endpoints and between side-effects and quality-of-life. It is known that genetics influence a patient’s risk of developing side-effects and a number of assays/approaches have been explored to assess a patient’s sensitivity to radiation. This prospective observational study will allow for the validation of the most promising biomarkers/approaches.

# 3.0 STUDY OBJECTIVES

#

## 3.1 Overall Design

This is an international observational cohort study. Eligible patients will have cancer of the breast (invasive or *in situ*), prostate or lung and be due to receive radical radiotherapy or adjuvant radiotherapy after breast conserving surgery or prostatectomy. Patients will be recruited from participating outpatient oncology clinics via cancer centres in multiple countries including Belgium, France, Germany, Italy, Spain, The Netherlands, UK and USA. Data on radiotherapy toxicity, non-genetic risk factors (e.g. dosimetry, chemotherapy use, age, diabetes, smoking history, co-morbidity) and quality of life will be collected at specified time points prospectively. Pre-treatment blood samples will be collected from all patients for downstream analyses. Patients will be required to have understood information about the study and given written informed consent.

##

## 3.2 Primary/ Secondary Objectives

**Primary Objective**

To establish a prospective cohort of patients undergoing radiotherapy for breast, prostate or lung cancers following local regimens and collecting standardised radiotherapy toxicity data, non genetic risk factor data and samples for biomarker assays for the study of determinants of radiotherapy side-effects.

**Secondary Objective**

To establish a comprehensive centralised database and sample collection as a resource for the prospective evaluation and validation of clinical models incorporating biomarker data to identify before treatment those cancer patients who are at risk of developing long term side-effects from radiotherapy.

## 3.3 Study Endpoints

Given the multiple endpoints of toxicity, REQUITE will focus on those that are considered most specific for radiotherapy, i.e. those that show a radiation dose response. In breast cancer, changes in breast appearance show a dose response relationship^[[24]](#footnote-24)^. In prostate cancer survivors, dose response relationships have been shown for bleeding and faecal incontinence^[[25]](#footnote-25)^. In lung cancer, dose and dose volume effects are strongly related to symptomatic late radiation toxicity^[[26]](#footnote-26)^. For example, V20 (the percentage of lung receiving >20 Gy) is considered a good predictor of lung toxicity^[[27]](#footnote-27)^.

Toxicity can be progressive and continue to develop many years following radiotherapy. There is evidence that moderate toxicity at early time points predicts for severe toxicity later. It is agreed that assessing toxicity at two years is ample time to express a significant proportion of late-effects^[[28]](#footnote-28)^. Using a two-year time point minimises loss to follow-up but ensures sufficient time for late-effects to emerge. However, in lung cancer patients, late toxicity develops six months following the start of treatment and, as patient survival is lower than for cancers of the prostate or breast, one-year toxicity is a more appropriate time point.

**Primary Endpoints**

- Breast: Any change in breast appearance at 24 months following start of radiotherapy
- Prostate: Rectal bleeding at 24 months following start of radiotherapy
- Lung: Dyspnea/ breathlessness at 12 months following start of radiotherapy

**Secondary Endpoints**

- Other toxicity endpoints including but not limited to: fibrosis, induration and vascular changes (breast); rectal incontinence, urinary toxicity and erectile dysfunction (prostate); dysphagia, oesophagitis (lung)
- Quality of Life (QoL)
- Maximum grade of toxicity during follow-up period

## 3.4 Data Collection

REQUITE will have a centralised, online database for recording physician and patient-reported toxicity data, as well as treatment, physics, co-morbidity, epidemiologic, and quality of life data (see Section 6.3). Completion of online questionnaires by patients in the clinic (PC or tablet) is the preferred method of data capture. However, paper questionnaires will be available for patients for collecting information on patient-reported toxicity, personal characteristics and QoL as required. All data completed using paper questionnaires must be entered into the electronic database by local study centre personnel. Health care professionals will complete online case record forms (CRFs) entering data directly into the REQUITE database.

**Treatment details and patient characteristics**

To maximise the ability to validate clinical models predicting toxicity, information must be collected on a number of variables including but not limited to: radiation doses received by surrounding normal tissues and the volumes irradiated, age, weight, height, smoking history (past/current smoker, intensity, duration, time since quitting), co-morbidity (e.g. diabetes, collagen vascular disease, diverticulosis, haemorrhoids, lung diseases), menopausal status (women), other treatments received (chemotherapy, anti-hormonal therapy, trastuzumab etc.) and tumour type relevant characteristics (e.g. histological type, TNM, ER/PR, HER2, grade etc.).

**Toxicity data**

Based on the Common Terminology Criteria for Adverse Events (CTCAE v4.0), toxicity questionnaires have been developed for both healthcare professionals and patients to assist with the collection, recording and managing of radiotherapy late effects^^[[29]](#footnote-29)^^. Side-effects are often under reported, so the inclusion of patient reported outcome measures that are both sensitive and reliable will help to improve on data capture^^[[30]](#footnote-30)^^. Information on acute toxicity will be collected according to standard clinical practice.

**Quality of Life data**

The EORTC QLQ-C30 questionnaire assesses the quality-of-life of cancer patients^^[[31]](#footnote-31)^^. It has been translated and validated into 81 languages and is used in more than 3,000 studies worldwide. Other tumour specific questionnaires including but not limited to the breast EORTC BR23 module will be collected.

## 3.5 Sample Collection

REQUITE will develop a centralised, accessible biobank linked to the observational study data. The Centre for Integrated Genomic Medical Research (CIGMR) will manage the biobank and co-ordinate with collection centres to organise shipping of frozen whole blood EDTA samples (Sample A) at regular intervals to Manchester, UK. This will ensure uniformity of storage prior to standardised DNA extraction. Additional blood samples collected at the same time point will either be stored locally for RNA extraction in future gene expression studies (Sample B), be used for immediate analysis in the live cell apoptosis or DNA repair assays (Sample C). See Section 6.2.

The CIGMR facility has multiple safeguards in place to maintain the integrity of frozen samples, including freezers monitored 24 hours a day, back-up power generation and ample spare freezer capacity for transferrals. All laboratory, data and management processes within the ISO9001:2008 certified biobank are fully ISO compliant and all processes carried out outside the biobank will seek to adhere to the ISO Quality Policy. REQUITE DNA samples will be held at two separate sites to mitigate against catastrophic loss.

## 3.6 Study Duration

Patients will remain on standard of care active treatment and will then be followed up for a minimum of 24 months. Follow-up to 5 years is encouraged where possible as part of routine clinical care. The study is expected to complete accrual within two years.

# 4.0 SELECTION OF STUDY PATIENTS

## 4.1 Inclusion Criteria

- Confirmed diagnosis of the specified tumour types, for lung cancer confirmation either by histology or based on radiological findings
- Patients suitable for adjuvant radiotherapy* for cancer of the breast (invasive or *in situ*) including breast patients receiving neo-adjuvant chemotherapy
- Patients suitable for radical radiotherapy or brachytherapy for prostate cancer; including post-prostatectomy patients
- Patients suitable for radical radiotherapy, sequential or concurrent chemoradiotherapy or stereotactic body radiation therapy for lung cancer
- No other malignancy in the last 5 years prior to treatment for the specified tumour types except basal cell or squamous cell carcinoma of the skin
- No evidence of distant metastases
- Patients able to provide a venous blood sample
- Breast patients consent to have photos taken of both breasts
- Willingness and ability to comply with scheduled visits, treatment plans and available for follow up within country of origin
- Greater than 18 years of age; no upper age limit
- The capacity to understand the patient information sheet and the ability to provide written informed consent

*Breast patients receiving chemotherapy should have completed their course of chemotherapy (anthracyclines) at least two weeks prior to radiotherapy commencing.

## 4.2 Exclusion Criteria

- Patients with metastatic disease
- Prior irradiation at the same site
- Planned use of protons
- High Intensity Focal Ultrasound (HIFU)
- Breast patients receiving concomitant chemo-radiation
- Male breast cancer patients
- Mastectomy patients
- Bilateral breast cancer
- Mental disability or patient otherwise unable to give informed consent and/or complete patient questionnaires
- Limited life expectancy due to co-morbidity
- Pregnant patients
- Partial breast irradiation
- Patients with breast implants if not removed during surgery
- Patients with known HIV infection/infectious hepatitis

# 5.0 RECRUITMENT OF PATIENTS

## 5.1 Number of patients required

A total of 5,300 patients are to be recruited. Approximate breakdown: 2,100 breast patients; 2,100 prostate patients and 1,100 lung patients.

## 5.2 Identifying patients

Eligible patients will be identified in the respective multi-disciplinary meetings and out-patient clinics (women’s clinics, urology and radio-oncology departments) at participating sites. A screening and recruitment log will be kept at each site recording all patients who were considered for the study. This will be a secure log holding patient names and details of those who consented to take part. For patients who did not fulfill the eligibility criteria or declined to take part, only a minimal dataset will be recorded, i.e. hospital number, gender and tumour site.

As REQUITE does not interfere with any standard or experimental, diagnostic or therapeutic procedures, patients may be included in REQUITE while participating in any other study/ trial.

The clinical leads representing the recruiting study centres have previously been involved in clinical trials and/or radiogenomics studies. Therefore, the logistics of patient recruitment already established at the participating clinics will be used for this study. Multiple clinics per recruitment centre will be involved, where necessary, to ensure adequate participant enrollment to reach the target sample size.

## 5.3 Consenting patients

Patients deemed eligible for entry into REQUITE will be provided with a verbal and written explanation of the study in accordance with local governance and regulatory departments. After adequate time has been given (a minimum of 24 hours), all queries have been addressed and the clinical team is confident that the patient understands the study and the necessity for long-term follow-up, patients will be consented onto the study.

Consent will be taken by a member of the study team who is GCP trained, suitably qualified and experienced and who has been delegated by the Principal Investigator to undertake this activity (and this delegation is clearly documented on the delegation log).

Consent will be specifically sought for the following:

- use of blood sample and/or DNA in molecular-genetic research
- use of blood sample and/or DNA in future genomic studies
- potential commercialisation

#

# 6.0 STUDY METHODOLOGY

## 6.1 Patient Registration

Issue of each patient’s unique study identifier will be done by allocation of the next sequential REQUITE kit (provided by CIGMR). Each kit will contain a number of bar-coded labels displaying the unique study identifier for that patient, as well as a bar-coded BD Vacutainer EDTA blood tube. The labels can be affixed to other blood tubes, consent form or patient notes as appropriate (see below).

Following signing of the consent form, the ‘Consent’ bar-code label from the next sequential REQUITE kit should be affixed to the consent form, and scanned into the appropriate table on the REQUITE centralised database. This will formally register the patient on the REQUITE study and represent their primary entry in the REQUITE database. The enrolment form confirming patient eligibility for the study should also be completed at this time.

##

## 6.2 Sample Collection and Tracking

Patients who are deemed eligible for the study following consent and all screening evaluations will be asked to gift pre-treatment blood samples that comprise ‘Sample A’ (10ml) and at least one of ‘Sample B’ (2.5ml) or ‘Sample C’ (10ml). A limited number of patients may be asked to give an additional blood sample to enable repeat analyses of the live cell assays for quality control purposes.

CIGMR will print and distribute all the bar-codes to be used in this study in individual REQUITE kits. Each bar-code label will display the following machine readable and human readable information:

- Human readable unique study identifier
- 1D barcode representing the unique study identifier
- Recruiting centre
- Sample type (EDTA blood, PAXgene or Lithium Heparin)

CIGMR will send out batches of individual REQUITE kits to each study centre. Each kit contains a bar-coded EDTA BD Vacutainer blood tube representing ‘Sample A’ in the REQUITE study. Depending on the recruiting site (see below) CIGMR will also provide additional bar-coded labels to be used on a PAXgene tube (Sample B) or a Lithium Heparin tube (Sample C). These labels should be affixed at site to the appropriate tube and blood collected at the appropriate time point (see below).

**Sample A**

- All patients in REQUITE (n ~5,300):

One 10ml EDTA blood sample will be collected either:

1) Prior to the start of radiotherapy for lung cancer patients;

2) Prior to the start of radiotherapy for prostate cancer patients;

3) Prior to the start of chemotherapy or radiotherapy for breast cancer patients*.

* The timing for collection of the blood sample from breast cancer patients is dependent upon the recruiting site (see below).

All EDTA bloods will be stored locally at -80°C as whole blood, no further processing is required. Following blood collection, study centres will scan the bar-code to complete a sample tracking form on the REQUITE centralised database, which can be accessed by CIGMR. This will provide accurate and consistent tracking of samples at study centres and will be used to update the CIGMR Laboratory Information Management System (LIMS).

CIGMR will liaise with each of the study centres regarding the return of frozen EDTA blood samples by courier at regular intervals e.g. every six months or as required. CIGMR will log and receipt the frozen blood samples immediately raising any discrepancies with the originating centre. At an appropriate time point they will process the blood samples to extract DNA; perform both a quality assessment and DNA quantification using Nanodrop technology and dilute to a standard concentration of at least one aliquot and store the undiluted and diluted DNA at two different geographical sites to guard against catastrophic loss prior to transfer to Source Bioscience for genotyping (see Section 6.6.1)

CIGMR will ensure continuous maintenance and management of their storage facility to ensure all equipment is fit for purpose and safe to use. As a certified biobank, they will ensure all laboratory, data and management processes are fully compliant with ISO9001:2008.

**Sample B**

- Patients recruited at CNFT, FPGMX, UGENT, ULEIC, MSSM, INT, KULEUVEN, MAASTRO or third parties associated with these centres (n ~3,500).

One 10ml PAXgene tube will be labelled with the corresponding PAXgene bar-code clearly displaying the same study identifier as used previously for the EDTA sample (Sample A). This PAXgene tube is pre-loaded with 7.5ml of RNA stabiliser and 2.5ml of blood will be drawn from the patient. Bloods will be collected at the same time as ‘Sample A’ either:

1) Prior to the start of radiotherapy for breast cancer or lung cancer patients;

2) Prior to the start of radiotherapy for prostate cancer patients.

This PAXgene blood sample will be stored locally at -80°C as whole blood for future RNA collection and extraction. Note, the samples for RNA analysis will not be used further in REQUITE, but will be a resource for future exploitation in biomarker discovery projects or similar.

**Sample C**

- Patients recruited at UMM, ULEIC, ICM or third parties associated with these centres (n ~1,800)

One 10ml Lithium Heparin (LiH) blood tube will be labelled with the corresponding LiH bar-code clearly displaying the same study identifier as used previously for the EDTA sample (Sample A). The LiH blood sample should be collected at the same time as ‘Sample A’ either:

1) Prior to the start of chemotherapy and radiotherapy for lung patients;

2) Prior to the start of radiotherapy for prostate cancer patients;

3) Prior to the start of chemotherapy and radiotherapy for breast cancer patients.

This sample will be retained in the recruitment centre for immediate analysis in the live cell apoptosis assay work. Each centre will make local arrangements for the transport of samples from the clinical facilities to the analysis laboratory, and temporary storage at 4°C (see Section 6.6.2). For comparative research and quality assurance, this sample may also be used for analysis using other DNA damage and repair assays (see Section 6.6.3).

**Repeat Sample C**

- Patients recruited at ULEIC or third parties associated with this centre (n = 200 maximum)

An additional 10ml Lithium Heparin (LiH) blood tube will be labelled with a duplicate LiH bar-code displaying the same study identifier as used previously. This LiH blood sample will be collected:

1) Prior to the start of radiotherapy from breast cancer patients who previously received chemotherapy.

Or

1. Following completion of radiotherapy at follow up visits

This sample will be retained in the recruitment city for immediate analysis in the live cell assays work. It will represent a repeat of ‘Sample C’ collected at an earlier time point and will be used for the live cell assay work to allow researchers to compare differences between samples collected pre-chemotherapy and post-chemotherapy or pre- and post- radiotherapy. Local arrangements will be made for the transport of samples from the clinical facility to the analysis laboratory, and temporary storage at 4°C (see Section 6.6.2).

**Unused material**

Unused material derived from samples A-C will be processed according to a common protocol followed by all participating sites. Subject to approval from the REQUITE management team this unused material specifically DNA, RNA, lymphocytes and plasma will be stored and made available for collaborators to use in externally funded and ethically approved projects (e.g. gene expression or epigenetic marker studies).

## 6.3 Data Collection

The primary method of data capture in REQUITE is the centralised online database. An online web browser will be available for patients to complete their questionnaires whilst in clinic, or alternatively at home if required. Where patients choose to use a paper based questionnaire then manual data entry must be performed by study centre personnel. Healthcare professionals will also have password protected access for completion of CRFs and file uploads (breast photos and physics data) as detailed below.

**Case Record Forms (CRFs)**

- Enrolment form (eligibility criteria)
- Blood collection form
- Patient factors form (epidemiology)
- Clinical and treatment data collection form
- Health professional toxicity data form (tumour specific; based on CTCAE v4.0)
- Patient reported outcomes questionnaire (tumour specific; based on CTCAE v4.0)
- EORTC Core 30 Quality of Life questionnaire (C30)
- Tumour specific questionnaires including but not limited to BR23
- Withdrawal form
- Outcome data form

**Time points for CRF collection**

CRFs should be completed at routine clinic appointments, or in conjunction with a telephone call e.g. if a patient is attending follow-up near home. Time points specifically include:

- Baseline (prior to start of radiotherapy or within first five days of radiotherapy) Lung cancer patients receiving SBRT should complete CRFs prior to, or on the first day of radiotherapy
- End of radiotherapy (1-2 days prior to or the last day of treatment) for breast and prostate patients. For brachytherapy patients: at the first routine follow-up visit following implantation.
- 3 months from start of radiotherapy (+/- 4 weeks) for lung cancer patients
- 3 months from start of radiotherapy (+/- 4 weeks) for breast cancer patients (ULEIC only)
- 6 months from start of radiotherapy (+/- 4 weeks) for lung cancer patients
- 12 months from start of radiotherapy (+/- 4 weeks)
- 24 months from start of radiotherapy (+/- 4 weeks)
- 36 months from start of radiotherapy (+/- 4 weeks)
- 48 months from start of radiotherapy (+/- 4 weeks)
- 60 months from start of radiotherapy (+/- 4 weeks)

See schedule of assessments for timings of completion of each CRF for each tumour site (Section 12.0).

**Physics File Uploads**

Uploading of physics data and CT images to the REQUITE database can be completed at any point in the follow-up pathway prior to the observational study end. All personal data should be removed before files are uploaded. Only the unique study identifier should be used.

- DICOM-RT files
- CT images and contouring
- DVH data

**Digital Photographs (breast cancer patients only)**

Photographs should be of sufficient quality to show any skin changes (including telangiectasia), breast shrinkage or retraction. Photographs should include both breasts to compare any change between the irradiated and unirradiated breast. Photographs must exclude the head.

Two anterior views of the chest are required, one with hands on the hips and the other with hands raised as far as possible above the head. One lateral view of the chest with hands above the head is also required.

Study centres are advised to follow the guidelines detailed in the REQUITE ‘Photographic Assessment Form’ (Form B4)’ for guidance on taking breast photographs for inclusion in this study. This will minimise inter-centre and intra-centre variation in photographic conditions.

**Time points for digital photographs (breast only)**

- Baseline (prior to start of radiotherapy or within first five days of radiotherapy)
- 24 months from start of radiotherapy (+/- 4 weeks)
- 36 months from the start of radiotherapy (+/- 4 weeks)
- 60 months from the start of radiotherapy (+/- 4 weeks)

## 6.4 Data Handling

All data entered into the REQUITE database by patients and healthcare professionals will be subject to an automatic comprehensive validation check program to identify missing, illogical and/ or inconsistent data before submission can be completed. In addition, the REQUITE data manager will assess data regularly and review any questionable data and correct any data entry errors with the help of the appropriate healthcare professionals.

The exact procedures for data entry and data clarification, including contact details for any related queries will be described in the study specific SOPs and instructions that will be sent to all REQUITE study centres as soon as they have completed the site initiation process. Reminders for any overdue data will also be sent out as necessary.

## 6.5 Radiotherapy

REQUITE is not a trial of radiotherapy, therefore the radiation dose and regimen is not prescribed. However, for breast patients receiving neo-adjuvant chemotherapy, it is important that radiotherapy should not commence until at least one month has elapsed following the end of chemotherapy.

All radiotherapy regimens will follow local standard of care decided by the treating clinician. Treatment details will be documented on the appropriate REQUITE CRF.

For lung cancer patients where possible, the heart should be outlined along with the pericardial sac. The pericardial sac surrounds the heart and extends superiorly to encompass the main pulmonary artery, the ascending aorta and the superior vena cava. Outlining should extend superiorly to the inferior limit of the aortic arch (the aortopulmonary window) and the superior limit of the trunk of the pulmonary artery if it can be identified on the radiotherapy planning CT scan.

For breast cancer patients, where possible, the following guidelines should be followed for heart delineation. Superiorly the heart starts just inferior to the left pulmonary artery. It includes the atria, ventricles, auricles, vessels and fat tissue within the pericardium. Since the cardiac vessels run in the fatty tissue within the pericardium, they should be included in the contours, even if there is no heart muscle visible in that area. Inferiorly, the heart blends with the diaphragm^^[[32]](#footnote-32)^^. For breast delineation, a wire should be used on the CT scan around the palpable breast tissue to define the peripheral edges of the breast. The deep edge is the superficial side of the pectoral muscle/thoracic wall. The superficial edge is the skin. Any visible glandular breast tissue outside these margins should also be included.

For prostate cancer patients, details of the rectum and bladder delineation should be specified on form P3, stating whether the entire rectum is delineated from anus to sigmoid; or alternatively, 2 cm cranial and caudal of the PTV; either as solid or hollow organ. For bladder delineation, it should be specified whether the entire bladder is delineated or the posterior wall only. Additionally, the filling level of the bladder should be stated.

## 6.6 Biomarker Assays

### 6.6.1 Genotyping

Genotyping will be carried out by commercial partner Source Bioscience in their facilities in Eire, UK or Germany.

The selection of genetic markers to be typed will be made by University of Cambridge in association with the University of Leicester and other members of REQUITE. The published literature will be evaluated for high quality genetic studies showing strong evidence of association with one of the clinical radiation toxicity endpoints. These markers will be collated into a single list such that patients for each cancer are typed for all markers. This maximises the chance of validating the associations whether they affect a single or multiple clinical endpoints.

Genotyping will be performed with the best available technology at the time, currently Fluidigm or GoldenGate custom arrays. CNVs would be typed either with a SNP genotyping platform or a qPCR based approach. A genome-wide approach may also be feasible, either by arrays or sequencing. If whole genome data are collected the information from the chosen markers will be extracted for the predictive markers, with the whole data being included in a separate association analysis. Control samples will be repeat typed in each laboratory to ensure reproducibility between sites.

**Genotyping Quality Assurance**

Quality assurance measures are already in place to produce negative controls on each experimental plate, and 5% of the samples from each plate will be duplicated on another plate as a reproducibility check. The genotype assay will be optimised on a panel of 80 unique DNA samples plus 12 duplicates and 4 negative controls. Duplicated samples must give greater than 99% concordance before the assay is accepted. These procedures are currently part of laboratory procedure.

### 6.6.2 Live cell apoptosis assays

The samples will be analysed at three centres: University of Leicester, UK; University of Heidelberg, Germany and University of Montpellier, France. Each centre will follow the current REQUITE standard protocol for ‘Radiation Induced Apoptosis Assays’ to reduce variability between centres. As a quality assurance measure for reproducibility both inter-lab and intra-lab variation will be assessed. Completion of the apoptosis assays will occur in Year 3 when patient `recruitment closes.

### 6.6.3 DNA damage and repair assays

These will be performed at the University of Leicester with lymphocytes from the sample used for the live cell apoptosis assay. Internal standard protocols for the relevant assays will be followed, including but not limited to the Comet and gamma-H2Ax assays.

# 7.0 STATISTICAL PLANS AND DATA ANALYSIS

## 7.1 Power calculations

**Live Cell Assays**

For the apoptosis assays with 800 breast, 600 prostate and 400 lung patient samples there is 80% power to detect relative risks (RR) of >1.31, 1.36 and 1.44 respectively, (α = 0.05, low apoptosis freq = 0.39, toxicity rate = 20%), or a 95% power to detect RR of >1.40, 1.47 and 1.57.

**Genotyping**

It is estimated that 2-year toxicity data (1-year for lung) will be available for 75% of enrolled patients. Based on effect sizes observed for genetic associations with radiation toxicity, a power calculation for the genetic assays shows that 1,575 patients for breast and prostate cancer each has 80% power to detect a RR of >1.56 for at least grade 2 toxicity (α = 5 x 10^-5^ for 1000 SNPs, allele freq = 0.25, toxicity rate = 20%), or with more stringent criteria a 90% power to detect a RR >1.66 (α = 1 x 10^-5^, allele freq = 0.25, toxicity rate = 20%). For 825 patients with lung cancer the detectable RR would be 1.78 and 1.92.

## 7.2 Statistical Data Analysis

In a first stage the data from the REQUITE multi-centre observational study will be used to validate published statistical models that use clinical and biomarker data to predict a patient’s risk of long-term side-effects following radiotherapy in patients with prostate, breast or lung cancer. Variables considered will be dose-volume data of organs and tissues at risk, dose fractions and overall treatment time, patient-related cofactors (e.g. age, weight, diabetes, and smoking), use of concurrent treatment (e.g. chemotherapy) and genotyping data. If necessary, individual clinical toxicity endpoints will be combined to derive tissue, organ or overall radiosensitivity measures (e.g. by STAT score or principal component analysis). Validation of risk factors will involve univariate analysis with Bonferroni correction and multivariate analysis. The strength of association of predictors will be assessed by calculation of the odds ratios.

Nomograms, logistic regression-, ordinal logistic regression-, and NTCP (normal tissue complication probability) models published in the literature will be considered for the prediction model validation. Validation of the prediction models in the REQUITE cohort will be evaluated by: (a) Calibration: assessing the agreement between observed and predicted probabilities using the Hosmer-Lemeshow “goodness-of-fit” test. (b) Discrimination: assessing the ability of the models to discriminate between those with and those without toxicity. For binary endpoints the area under the receiver operating curve (ROC) will be used with derivation of sensitivity and specificity for a chosen probability threshold. For polychotomous endpoints c-statistic will be used as rank order statistic. (c) Clinical usefulness: the “net benefit” (NB) will be calculated (NB=(TP−wFP)/N, where TP is the number of true-positive classifications, FP the number of false-positive classifications). w is a weight equal to the “ratio of harm to benefit” (w=cutoff/(1−cutoff), where cutoff is the probability threshold which is chosen to define the treatment rule (e.g.: the level of non-acceptable toxicity)). A clinically useful model should have an NB>cut-off.

In a second stage existing models will be improved and extended using the biomarker data (SNPs, apoptosis data) in large cohorts recruited via the REQUITE study. Derivation of these models will involve separating cohorts into discovery and validation cohorts. The following performance measures will be considered for improvement and extension of existing models: (a) Likelihood-ratio test: comparing the fits of two models, the null model versus the alternative model. (b) Reclassification table (i.e. percent of individuals who change risk category when applying an advanced model). (c) Net Reclassification Improvement (NRI; i.e. for patients with toxicity any upward shift in risk classes implies prediction improvement and any downward shift indicates reduced reclassification. The reverse holds for patients without toxicity.

In both stages appropriate statistical methods are used as described in the statistical operating procedure (SOP) for statistical analysis.

# 8.0 COMPLIANCE, DATA PROTECTION AND DATA SHARING

## 8.1 Compliance

REQUITE will be conducted according to the protocol, relevant Standard Operating Procedures (SOPs), ICH-GCP and relevant national regulatory requirements.

By participating in the REQUITE study, the Principal Investigators at each study centre are confirming agreement to ensure that:

- Sufficient data are recorded for all participating patients to enable accurate linkage between hospital records and CRFs;
- Source data and all study related documentation are accurate, complete, maintained and accessible for monitoring and audit visits;
- Study-related monitoring, audits, and regulatory inspection(s) are permitted and direct access to source data/documents is provided as required.

## 8.2 Data Protection

Patients will be assigned a unique study identifier by allocation of the next sequential bar-coded ‘Sample A’ blood tube (provided by CIGMR), which will be used throughout their participation in the study. Any personal data recorded will be regarded as confidential, and any information which would allow individual patients to be identified will not be released into the public domain.

Each investigator should keep the screening and recruitment log and all other study documents (including participant’s written consent forms) which are to be held at the recruiting study centre, in strictest confidence. The investigator must ensure the patients’ confidentiality is maintained.

All investigators and researchers involved with the study must comply with the national requirements for data protection with regard to the collection, storage, processing and disclosure of personal information and agree to uphold the appropriate core principles.

Patient notes and study files at site must be kept in a secure storage area with limited access. Access to the REQUITE database will be strictly limited via usernames and passwords. Published results will not contain any personal data that could allow identification of individual patients.

## 8.3 Data Sharing

The REQUITE steering committee will eventually seek to combine samples and data collected in this observational study with those collected in other radiogenomics studies. This collaboration via the Radiogenomics Consortium with other researchers worldwide should enable a significant sample size to be reached via meta-analyses for detailed study into the genetic differences which lead to variation in radiosensitivity between individuals. Consent will be sought from participants to allow their donated samples and data to be shared with other research groups. Before any data are sent to an external research group, a written proposal detailing the proposed work, including the study hypothesis, inclusion and exclusion criteria, type of statistical analysis, and the variables to be considered, must be submitted to and approved by the REQUITE steering committee. Any samples and data transferred to third parties would not contain any personal information about the patients and therefore confidentiality would be maintained.

# 9.0 ETHICAL AND REGULATORY REQUIREMENTS

Research Ethics approval for this study has been sought from NRES Committee North West – Greater Manchester East and a favourable opinion granted (ref. 14/NW/0035). Individual participating centres must apply to their local research governance departments for management permission prior to opening.

The study will be conducted in accordance with the current approved protocol, the Declaration of Helsinki, ICH Guidelines for Good Clinical Practice (ICH GCP), relevant regulations and standard operating procedures.

The local Principal Investigator (PI) must ensure that the study protocol, patient information sheet, consent form, family doctor letter and submitted supporting documents have been approved by the appropriate regulatory body(ies) and research ethics committee(s) prior to any patient recruitment.

Any agreed substantial amendments must also be submitted and receive ethical and regulatory approval prior to implementation. It is the responsibility of the PI at each site to ensure that the study has all the necessary approvals in place. A site initiation meeting must be completed prior to each study centre opening to recruitment.

## 9.1 Sponsor and Indemnity

The University of Manchester will act as sponsor for this study. The contact point is:

Professor Nalin Thakker

Associate Vice-President (Research Integrity)

The University of Manchester

Oxford Road, Manchester M13 9PL

Email: [research-governance@manchester.ac.uk](mailto:research-governance@manchester.ac.uk)

Telephone: 0161 275 8795

The University of Manchester has insurance for research involving human subjects that provides cover for legal liabilities arising from its actions or those of its staff, subject to policy terms and conditions. For participating sites which are part of the NHS, the NHS indemnity scheme will also apply.

## 9.2 Informed Consent

A patient information sheet will be presented to potential participants detailing the nature of the study, the implications of participating and any potential risks or inconveniences involved in taking part. It will be clearly stated that the participant is free to withdraw from the study at any time, for any reason, without prejudice to future care and with no obligation to give the reason for withdrawal. The participant will be allowed sufficient time (a minimum of 24 hours) to consider the information and decide whether to take part before consent is sought. Written informed consent will then be obtained by means of patient dated signature and dated signature of the person who presented and obtained the informed consent.

Molecular and genetic information collected in this research will be in a linked anonymised format such that individual patient information will not be available to anyone in the research team, holding or analysing the data. However, unique study identifiers will be assigned that allow the supplier of the data, such as the person who obtained informed consent, to be able to identify people from it.

## 9.3 Patient Withdrawal

Lung cancer patients will be withdrawn from the study if they experience a recurrence or second malignancy within the thorax (including breast cancer).

Breast cancer patients will be withdrawn from the study if they have a secondary mastectomy due to relapse.

Patients wishing to withdraw from the study will not be replaced. If a participant decides to withdraw, their coded blood samples and information will be retained for use within REQUITE and for future medical research unless the patient makes a specific request otherwise. If specifically requested (either verbally or in writing to a member of the research team), the blood samples will be destroyed and the medical information removed from the REQUITE database.

# 10.0 STUDY MANAGEMENT AND OVERSIGHT ARRANGEMENTS

## 10.1 Study Management Group

A Study Management Group (SMG) will be established and will include those individuals responsible for the day-to-day management of the study including the Chief Investigator, co-investigators and identified collaborators, Principal Investigators, the study statistician and the study manager(s). The SMG will monitor overall progress of the study to ensure the protocol is adhered to and will take appropriate action to safeguard the patients and the quality of the study where appropriate.

The SMG will hold teleconferences at least quarterly once the study is actively recruiting. Minutes will be taken at SMG meetings and copies of the minutes will be filed in the Study Master File. The study manager and Chief Investigator will ensure that all relevant issues and actions discussed during the meeting are followed up and resolved. Details of significant issues will be made available to participating sites and other relevant parties as appropriate.

## 10.2 Study Data

Submitted data will be checked for errors, inconsistencies and omissions. If missing or questionable data are identified, the REQUITE data manager will request that the data be clarified.

##

## 10.3 Study Monitoring

There will be a set-up/ site initiation meeting at each main study centre (personnel from third parties who are recruiting patients should also attend). This is very important to ensure that the correct procedures and guidelines are fully understood and that all members of staff working at each study centre have an opportunity for dedicated training on the protocol, data collection, electronic data input, sample collection and storage procedures.

There will also be a subsequent monitoring visit per study centre. The purpose of these visits is:

- To verify that the rights and well-being of patients/participants are protected.
- To verify accuracy, completion and validity of reported study data from the source documents.
- To evaluate the conduct of the study within the institution with regard to compliance with the currently approved protocol, GCP and with the applicable regulatory requirements

# 11.0 PUBLICATION

For the main publication(s) of this study, it is anticipated that all contributors will be authors with the proviso that clinicians contributing patients must have contributed 20 patients or more. A formal publications policy will be generated, which all participants will be asked to sign up to.

The main results of the REQUITE study will be published in a peer-reviewed journal, on behalf of all collaborators. The manuscript will be prepared by a writing group, appointed from amongst the REQUITE Steering Committee and high accruing clinicians. All recruiting study centres and clinicians will be acknowledged in this publication. All presentations and publications relating to the study must be authorised by the Steering Committee. No investigator may present or attempt to publish data relating to REQUITE without prior permission from the REQUITE Steering Committee.

# 12.0 SCHEDULE OF ASSESSMENTS

Additional annual follow-up beyond 24 months up to 5 years is encouraged for all patients recruited to REQUITE.

## 12.1 Breast Patients

| **ACTIVITY** | **FORM LABEL** | **TIME POINT** | | | | |
| --- | --- | --- | --- | --- | --- | --- |
|  |  | **Baseline (prior to radiotherapy unless otherwise indicated)^** | **End of radiotherapy*** | **3 months from start of radiotherapy^+^** | **12 months from start of radiotherapy^+^** | **24 months from start of radiotherapy^+^**  ***(1° endpoint)***  ***Also yearly to 60 months*** |
| **Enrolment Form** | B1 | X |  |  |  |  |
| **Consent** | RQ1 | X |  |  |  |  |
| **Blood Collection^** | RQ2 | X^ |  |  |  |  |
| **Patient Factors Form** | B2 | B |  |  | F | F |
| **Clinical and Treatment Data Collection Form** | B3 |  | X |  |  |  |
| **Photographic Assessment Form** | B4 | X |  |  |  | X |
| **Health Professional Toxicity Data Form** | B5 | X | X | X | X | X |
| **EORTC C30** | B6a | X | X | X | X | X |
| **EORTC BR23** | B6b | X | X | X | X | X |
| **MFI** | B6c | X | X | X | X | X |
| **GPAQ** | B6d | X | X | X | X | X |
| **HBIS** | B6e | X | X | X | X | X |
| **Withdrawal Form** | B7 |  |  |  |  | X |
| **Outcome Data Form** | B8 |  |  |  |  | X |

^ Consent and blood collection can either be prior to chemotherapy or radiotherapy (see Section 6.2)

* 1-2 days prior to end of radiotherapy or last day

^+^ Plus/ minus four weeks

X: Same version for every time point

B: Baseline version

F: Follow-up version

## 12.2 Prostate Patients

| **ACTIVITY** | **FORM LABEL** | **TIME POINT** | | | |
| --- | --- | --- | --- | --- | --- |
|  |  | **Baseline (prior to radiotherapy)^** | **End of radiotherapy*^&^** | **12 months from start of radiotherapy^+^** | **24 months from start of radiotherapy^+^**  ***(1° endpoint)***  ***Also yearly to 60 months*** |
| **Enrolment Form** | P1 | X |  |  |  |
| **Consent** | RQ1 | X |  |  |  |
| **Blood Collection** | RQ2 | X |  |  |  |
| **Patient Factors Form** | P2 | B |  | F | F |
| **Clinical and Treatment Data Collection Form** | P3 |  | X |  |  |
| **Health Professional Toxicity Data Form** | P4 | X | X | X | X |
| **Patient Reported Outcomes Questionnaire** | P5a | X | X | X | X |
| **EORTC C30** | P5b | X | X | X | X |
| **MFI** | P5c | X | X | X | X |
| **GPAQ** | P5d | X | X | X | X |
| **Withdrawal Form** | P6 |  |  |  | X |
| **Outcome Data Form** | P7 |  |  |  | X |

^ Prior to radiotherapy

* 1-2 days prior to end of radiotherapy or last day

^&^ For brachytherapy patients, the end of treatment time point should take place at the first routine follow-up visit following implantation

^+^ Plus/ minus four weeks

X: Same version for every time point

B: Baseline version

F: Follow-up version

##

## 12.3 Lung Patients

| **ACTIVITY** | **FORM LABEL** | **TIME POINT** | | | | |
| --- | --- | --- | --- | --- | --- | --- |
|  |  | **Baseline (prior to radiotherapy)** | **3 months from start of radiotherapy^+^** | **6 months from the start of radiotherapy^+^** | **12 months from start of radiotherapy^+^**  ***(1° endpoint)*** | **24 months from start of radiotherapy^+^**  ***Also yearly to 60 months*** |
| **Enrolment Form** | L1 | X |  |  |  |  |
| **Consent** | RQ1 | X |  |  |  |  |
| **Blood Collection** | RQ2 | X |  |  |  |  |
| **Patient Factors Form** | L2 | B | F | F | F | F |
| **Clinical and Treatment Data Collection Form** | L3 |  | X |  |  |  |
| **Health Professional Toxicity Data Form** | L4 | X | X | X | X | X |
| **Patient Reported Outcomes Questionnaire** | L5a | X | X | X | X | X |
| **EORTC C30** | L5b | X | X | X | X | X |
| **MFI** | L5c | X | X | X | X | X |
| **GPAQ** | L5d | X | X | X | X | X |
| **Withdrawal Form** | L6 |  |  |  |  | X |
| **Outcome Data Form** | L7 |  |  |  |  | X |

^+^ Plus/ minus four weeks

X: Same version for every time point

B: Baseline version

F: Follow-up version.

# 13.0 SUB-STUDY 1: REQUITE-AB

## 13.1 Summary

| **TITLE** | REQUITE Acute Breast (REQUITE-AB) |
| --- | --- |
| **STUDY DESIGN** | Observational/ cohort study using full REQUITE methodology and breast patient sample |
| **TARGET DISEASE** | Cancer of the breast |
| **PRIMARY STUDY OBJECTIVE** | To establish a prospective cohort of breast cancer patients undergoing radiotherapy for breast cancer according to local regimens, and to collect standardised radiotherapy toxicity data, non-genetic risk factor data and samples for biomarker assays for the study of determinants of acute radiotherapy side-effects. |
| **SECONDARY STUDY OBJECTIVES** | To validate clinical models incorporating biomarker data integrating clinical and patient-reported outcomes, to identify before treatment those cancer patients who are at risk of developing acute side-effects from radiotherapy. |
| **PRIMARY ENDPOINTS** | - Acute skin toxicity (breast) - Quality of life (change patient-reported outcomes scores) |
| **SECONDARY ENDPOINTS** | - Change in breast appearance at 24 months following start of radiotherapy (breast) - Other toxicity endpoints including but not limited to: fibrosis, induration and vascular changes (breast) - Surgical complications including but not limited to: wound breakdown and need for revision surgery - Maximum grade of toxicity during follow-up period |
| **STUDY POPULATION** | Any patient over 18 years of age with primary cancer of the breast who are going to receive adjuvant radiotherapy after breast surgery. |
| **RECRUITMENT TARGET** | As per REQUITE main study breast sample (n = 2,100). |
| **FOLLOW UP VISITS** | As per REQUITE main study. |
| **STUDY DURATION** | As per REQUITE main study. |
| **SAMPLE COLLECTION** | As per REQUITE main study. |
| **POWER CALCULATIONS** | The same calculations as for the main REQUITE study apply. Based on effect sizes observed for genetic associations with radiation toxicity, a power calculation for the genetic assays on 1,575 breast patients (75 % of enrolled patients) has 80% power to detect a RR of >1.56 for at least grade 2 toxicity (α = 5 x 10^-5^ for 1000 SNPs, allele freq = 0.25, toxicity rate = 20%), or with more stringent criteria a 90% power to detect a RR >1.66 (α = 1 x 10^-5^, allele freq = 0.25, toxicity rate = 20%). |

## 13.2 Background

### 13.2.1 Radiotherapy in breast cancer

After surgery, radiotherapy is the second most commonly used treatment for breast cancer. It reduces the risk of local recurrence with a modest reduction in overall mortality.^[[33]](#footnote-33)^ Over 70 % of breast cancer patients undergo post-operative radiotherapy as part of their treatment. It is indicated after breast-conserving surgery (lumpectomy) and for high-risk patients after complete removal of the breast (mastectomy).^[[34]](#footnote-34)^

Radiotherapy is associated with a spectrum of side effects (toxicity) in the surrounding normal tissues. Acute toxicity occurs within 90 days of treatment and affects high turnover tissues such as the skin, whereas late toxicity occurs more than 90 days after treatment and can persist for life. Late effects in the breast include fibrosis, atrophy, and telangiectasia (dilated small blood vessels under the skin).^[[35]](#footnote-35)^

### 13.2.2 Acute radiotherapy toxicity – impact on treatment outcomes

While late side-effects of radiotherapy are concerning due to their potential irreversibility, there is an increasing interest amongst breast surgeons in acute radiotherapy toxicity. The predominant clinical acute side-effect in the breast is an adverse skin reaction. The vast majority of patients undergoing radiotherapy report skin changes.^[[36]](#footnote-36)^ Although the START trial provided a detailed description of radiotherapy side-effects, it did not examine the acute phase during and immediately after radiotherapy.^[[37]](#footnote-37)^

Amongst breast surgeons there is an increasing awareness of the impact of radiotherapy on breast cosmesis and patient’s QoL. Against growing patient expectations and increasing technical expertise, rates of oncoplastic procedures after lumpectomy^[[38]](#footnote-38)^ and breast reconstruction after mastectomy continue to rise.^[[39]](#footnote-39)^ If sufficiently severe, an acute radiotherapy skin reaction can have detrimental effects on any form of breast reconstruction. This also predisposes the patient to chronic complications such as implant capsular contracture or scarring, which may require further reconstructive procedures.^[[40]](#footnote-40)^

Surgeons are invariably influenced in their treatment recommendations by the potential complications of other breast cancer treatments such as radiotherapy.^[[41]](#footnote-41)^ Post-mastectomy radiotherapy to the chest wall confers a survival benefit when combined with systemic adjuvant therapies.^[[42]](#footnote-42)^ In the absence of any randomized evidence, there is an on-going debate how to best manage patients opting for immediate reconstruction, especially when one cannot predict with certainty whether radiotherapy is needed until the full post-operative histology is available.^[[43]](#footnote-43)^ Being able to stratify individuals according to risk of radiation toxicity would enable breast surgeons and their patients to decide on the most appropriate operation.

### 13.2.3 Genetic markers of acute radiotherapy toxicity

It is now understood that individual sensitivity to radiation is also determined by genetic variation.^[[44]](#footnote-44)^ In order to identify genetic markers of normal tissue radiosensitivity, the main approach taken by investigators has been to type SNPs (single nucleotide polymorphisms) in the genome of patients undergoing radiotherapy. SNPs represent relatively common genetic alterations that typically have low effect sizes. Through a number of case-control studies, in which SNPs at candidate loci were genotyped across patients with or without radiotherapy side-effects, several predictive genetic markers have been identified.^[[45]](#footnote-45)^,^[[46]](#footnote-46)^

To foster collaboration in the field, the International Radiogenomics Consortium was formed to facilitate the pooling of patient cohorts and datasets.^[[47]](#footnote-47)^ Within the Consortium, the first genetic associations with acute and late toxicity have been replicated.^[[48]](#footnote-48)^,^[[49]](#footnote-49)^ Several genome wide association studies (GWAS), in which patients are genotyped for a large number of common SNPs, are under way or about to be published.^[[50]](#footnote-50)^ These markers now require prospective validation.

### 13.2.4 Rationale for the study

The time is right to validate known genetic markers and clinical predictors to improve prediction of acute radiotherapy side-effects in breast cancer patients. PROMs and clinical toxicity outcomes should be incorporated into any predictive model of acute radiotoxicity. Any previously developed predictive model will require systematic validation across a multi-centre patient cohort, and this can be provided by the REQUITE breast cancer sample.

## 13.3 Sub-study research plan

### 13.3.1 Study objectives and design

The aim of this sub-study is to integrate clinical and genetic variables to improve prediction of acute radiotherapy toxicity in breast cancer patients, with the following objectives:

- To establish a prospective cohort of breast cancer patients undergoing radiotherapy for breast cancer according to local regimens, and to collect standardised radiotherapy toxicity data, non-genetic risk factor data and samples for biomarker assays for the study of determinants of acute radiotherapy side-effects.
- To validate clinical models incorporating biomarker data integrating clinical and patient-reported outcomes, to identify before treatment those cancer patients who are at risk of developing acute side-effects from radiotherapy
- To integrate clinical and patient-reported end-points within a prospective cohort study of acute radiotherapy side-effects

This sub-study will use the breast cancer sample from the main REQUITE study recruited at participating centres in multiple countries including Belgium, France, Germany, Italy, Spain, The Netherlands, UK and USA. Data on radiotherapy toxicity, non-genetic risk factors (e.g. dosimetry, chemotherapy use, age, diabetes, smoking history, co-morbidity) and quality of life will be collected at specified time points prospectively. Pre-treatment blood samples will be collected from all patients for downstream analyses. Patients will be required to have understood information about the study and given written informed consent.

### 13.3.2 Patient selection and recruitment

The inclusion and exclusion criteria for the main REQUITE study shall apply.

### 13.3.3 Study methodology and data analysis

The sample in this sub-study will consist of the breast cancer patients recruited under the main REQUITE study. Patients will be recruited, sampled and tracked using the method described in the main study. Biomarker assays, data collection and analysis will be performed according to the method of the main study.

The primary endpoints in this study will be acute radiotherapy reaction scored using the REQUITE standard B5 outcomes form and change in quality of life scores during the course of radiotherapy before, immediately after, and at 3 months from the beginning of radiotherapy (not all sites), scored using the REQUITE standard B6 forms. Secondary endpoints will be the late toxicity endpoints from the main study, maximum toxicity during follow-up and any surgical complications including but not limited to wound breakdown and need for revision surgery.

## 13.4 Sub-study research arrangements

### 13.4.1 Compliance and data protection

This sub-study will be conducted according to the same standards as the main REQUITE study, including the protocol, standard operating procedures (SOPs), ICH-GCP, and other regulatory requirements. The Principal Investigator at the site involved confirms compliance with the main study’s and local research governance procedures.

The same data protection procedures apply as in the main REQUITE study.

### 13.4.2 Ethical and regulatory requirements

Research Ethics approval for this sub-study will be sought from the NRES Committee North West – Greater Manchester East by way of major amendment to the previously approved protocol (ref. 14/NW/0035). Permission to conduct the sub-study will be sought from the R&D department at participating centres.

Patients will be recruited onto the study using the standard REQUITE patient information sheet and consent forms and as per consent procedure outlined in the main study. The withdrawal criteria and procedure from the main REQUITE study shall apply.

### 13.4.3 Management and oversight arrangements

This sub-study will be overseen by the main REQUITE Study Management Group (SMG), as the majority of patients in this sub-study will come from the main study. Study data will be overseen by the REQUITE data manager. The conduct of this sub-study will be monitored as part of the arrangements for the main study.

The University of Manchester will remain as sponsor for this sub-study. The contact point is:

Professor Nalin Thakker

Associate Vice-President (Research Integrity)

The University of Manchester

Oxford Road, Manchester M13 9PL

Email: [research-governance@manchester.ac.uk](mailto:research-governance@manchester.ac.uk)

Telephone: 0161 275 8795

The University of Manchester has insurance for research involving human subjects that provides cover for legal liabilities arising from its actions or those of its staff, subject to policy terms and conditions. For participating sites which are part of the NHS, the NHS indemnity scheme will also apply.

### 13.4.4 Sub-study publication

For the main publication(s) arising from this sub-study, it is anticipated that all contributors will be authors with the proviso that clinicians contributing patients must have contributed 20 patients or more. This includes all centres where breast cancer patients have been recruited.

A formal publications policy is due to be generated by the REQUITE SMG, which all participants will be asked to sign up to. All presentations and publications relating to the sub-study will require authorisation by the Steering Committee.

The main results of the REQUITE-AB sub-study will be published in a peer-reviewed journal, on behalf of all collaborators. The manuscript will be prepared by a writing group, appointed from participating centres and the REQUITE Steering Committee.

# 14.0 SUB-STUDY 2: REQUITE-AB-QOL

## 14.1 Summary

| **TITLE** | REQUITE Acute Breast Qualitative Interview Study (REQUITE-AB-QOL) |
| --- | --- |
| **STUDY DESIGN** | Qualitative study embedded within the REQUITE main study conducting semi-structured interviews with a purposive sample of breast cancer patients recruited into the main study |
| **TARGET DISEASE** | Cancer of the breast |
| **PRIMARY STUDY OBJECTIVE** | To explore participants’ attitudes to and beliefs about a predictive tool for acute radiotherapy side-effects and any impact on participants’ decision-making |
| **SECONDARY STUDY OBJECTIVE** | To explore participants’ experience of breast radiotherapy. |
| **PARTICIPATING SITES** | University of Leicester |
| **STUDY POPULATION** | Breast cancer patients recruited into the main REQUITE or REQUITE-AB sub-study |
| **RECRUITMENT TARGET** | Sample size to be determined by qualitative data generated from participants, but it is anticipated that no more than 25 to 30 patients will be interviewed. |
| **FOLLOW UP VISITS** | Single interview to take place on or shortly after completion of radiotherapy. |
| **STUDY DURATION** | Patients to be recruited over the course of the 24-month recruitment period of the main REQUITE study |
| **SAMPLE COLLECTION** | Single semi-structured interview, digitally recorded and transcribed |
| **POWER CALCULATIONS** | Given the qualitative methodology, there is no power calculation. |

## 14.2 Background

### 14.2.1 Radiotherapy impact on quality of life (QoL)

A relative lack of evidence about the effects of breast cancer treatments on QoL can impair doctor-patient communication and limit the patient’s understanding of their treatment. Breast cancer patients face difficult treatment decisions and should be guided by meaningful information about expected QoL and radiation toxicity outcomes.

The main REQUITE study and the REQUITE-AB sub-study seek to collect QoL endpoints using validated patient-reported outcome measure (PROM) instruments in addition to genotyping data and clinical end-points. If clinicians are to inform patients more accurately about the risks and impact of side-effects, this will require accurate prediction tools incorporating PROM endpoints. However, the acceptability of any such tool to breast cancer patients should also be evaluated.

More than half of all women due to undergo breast cancer radiotherapy are anxious about side-effects and changes to the appearance of their breast.^[[51]](#footnote-51)^ With a focus on acute side-effects, recent qualitative research has illustrated a predominantly negative perception of radiotherapy.^[[52]](#footnote-52)^ The same authors also published a thematic analysis of semi-structured interviews with 20 women immediately after breast radiotherapy.^[[53]](#footnote-53)^

### 14.2.2 Rationale for the study

Based on their experience of breast radiotherapy, patients’ views on a predictive tool for acute radiotherapy side-effects should be explored, to determine how any such test should be implemented in clinical practice and how it would impact on patients’ decision-making. Research is needed to ensure that any such decision-making tool is acceptable to for breast cancer patients.

## 14.3 Sub-study research plan

### 14.3.1 Study objectives and design

The main objective of the REQUITE-AB-QOL study is to explore patients’ attitudes to and beliefs about a predictive tool for acute radiotherapy side-effects and the impact on patients’ decision-making around different breast cancer treatments.

Embedded within the main cohort study, qualitative data will be collected through semi-structured interviews with a sample of patients on completion of radiotherapy at participating centres. This will serve to explore patients’ attitudes and beliefs about a future predictive test for radiotherapy side-effects or treatment decision-making tool and to assess the acceptability of such a tool.

### 14.3.2 Patient selection and recruitment

A purposive sample of breast cancer patients recruited into the main REQUITE study or the REQUITE-AB sub-study at the participating centre will be interviewed. Eligible patients will be approached if they would be willing to participate in an audio-recorded in-depth interview. There is no set number of participants to be recruited, given the iterative nature of this part of the research. Sample size will be determined by data generated from participants until no new topics emerge and thematic saturation is reached.^[[54]](#footnote-54)^ However, it is anticipated that no more than 25 to 30 patients will be interviewed. It is expected that the sample will include adequate representation of age, ethnicity, different breast sizes, types of breast surgery, co-morbidities (e.g. diabetes or anxiety/depression) and degrees of skin toxicity.

Patients will be consented for the interviews using an additional consent form and additional patient information sheet.

### 14.3.3 Study methodology and data analysis

Each patient interview will take place on or shortly after completion of radiotherapy, anticipating that most patients will have experienced acute skin toxicity by then. Patients will be offered a choice of location – either a private room at the hospital away from the clinical areas or to be interviewed in their own home. If patients are interviewed at home, then a check-in/check-out procedure will be followed by the interviewer with the hosting University department and the interviewer must remain contactable by mobile phone. Interviews will last approximately an hour, but no more than one and a half hours. Each interview will be recorded on a digital Dictaphone and transcribed verbatim and fully anonymised, using professional transcription services.

As some of the issues explored in this part of the project will be personally sensitive, individual interviews will be conducted rather than focus groups. While following an interview guide, this approach also allows participants to be probed further to get a rich picture of their experience. The researcher conducting interviews will not be involved in the patient’s usual medical care, but may be medically trained. At the beginning of the interview, it will be explained to patients that the interviewer will not be able to answer questions concerning their own treatment, but will have knowledge and training to direct any patient queries to the correct healthcare professional. Patients’ responses will be completely anonymised for the data analysis.

At the start of the interview, patients will be asked to talk about their experience of breast radiotherapy without interruption by the interviewer. This will serve to ease the participant into the interview and to build up a rich background picture of their treatment experience. The idea of a predictive test for acute radiotherapy side-effects and reasoning behind this will then be introduced by the interviewer. Participants will also be shown different fictional case vignettes with reports; one suggesting risk of severe toxicity, one suggesting mild or no toxicity only, and one inconclusive test result. Patients will be reassured that severe toxicity is relatively uncommon. After engaging with this information, the patient will be invited to describe their thoughts and feelings.

Following the initial response, the interview guide will include probes in areas of further inquiry relating to the treatment experience and the feasibility and implementation of any predictive test – in particular, acceptability, demand, and practicality, as well as integration into the treatment decision-making process.^[[55]](#footnote-55)^ For example, participants will be asked, if they thought if a predictive test would have been appropriate for them, how interested they would be in getting a report, and at what level of risk the result would influence their treatment decision and options for alternative treatment. To ensure that important themes have not been missed, the interview will be concluded with the question: ‘Is there anything else you would like to tell me?’

Emerging themes will be identified through systematic coding of the transcripts, using Nvivo software or similar. Transcripts will be analysed independently by the researcher and checked with a co-researcher every three to five interviews to ensure reliability of the data coding. Thematic analysis will take place in parallel to data collection through familiarization with the data and constant comparison of transcripts to inform further data collection until thematic saturation is achieved.

Thematic codes will subsequently be sorted into broader themes. On completion of the interviews, coding and themes will be finalised by the research team, following published RATS guidelines for qualitative research.^[[56]](#footnote-56)^

Thematic analysis is a particular type of qualitative analysis focused on recognising, analysing, and reporting repeating themes across a data set.^[[57]](#footnote-57)^ It is not tied to a specific theory or epistemology, therefore one has to be explicit why this method is used. First, thematic analysis in this study will be driven by an interest in understanding whether a test for acute radiotoxicity would be acceptable to breast cancer patients. Secondly, although this approach may lose some of the depth of a more abstract analysis, the aim is to generate a thematic description of the patients’ feelings, attitudes and concerns about any such test. Themes identified in relation to the treatment experience may also be used to qualify individual scores from the PROM questionnaires obtained as part of the REQUITE study.

## 14.4 Sub-study research arrangements

### 14.4.1 Compliance and data protection

All investigators and researchers involved with the study must comply with the national requirements for data protection with regard to the collection, storage, processing and disclosure of personal information and agree to uphold the appropriate core principles.

Patient notes and study files at site must be kept in a secure storage area with limited access. Interviews will be audio-recorded and then transcribed in anonymized form and stored on password-protected university computers. Anonymized data will be emailed in encrypted format or uploaded via secure servers

Any access required to the REQUITE database will be strictly limited via usernames and passwords. Published results will not contain any personal data that could allow identification of individual patients.

### 14.4.2 Ethical and regulatory requirements

The study will be conducted in accordance with the approved protocol, the Declaration of Helsinki, ICH Guidelines for Good Clinical Practice (ICH GCP), relevant regulations and standard operating procedures.

Research Ethics approval for this sub-study will be sought from the NRES Committee North West – Greater Manchester East by way of major amendment to the previously approved protocol (ref. 14/NW/0035). Permission to conduct the sub-study will be sought from the R&D department at participating centres.

The local Principal Investigator (PI) must ensure that the study protocol, patient information sheet, consent form, family doctor letter and submitted supporting documents have been approved by the appropriate regulatory bodies and research ethics committee(s) prior to any patient recruitment.

Any agreed substantial amendments must also be submitted and receive ethical and regulatory approval prior to implementation. It is the responsibility of the PI at each site to ensure that the study has all the necessary approvals in place. A site initiation meeting must be completed prior to each study centre opening to recruitment.

Patients will be recruited onto the sub-study using a separate REQUITE-AB-QOL patient information sheet and separate consent form. A patient information sheet will be presented to potential participants detailing the nature of the study, the implications of participating and any potential risks or inconveniences involved in taking part. It will be clearly stated that the participant is free to withdraw from the study at any time, for any reason, without prejudice to future care and with no obligation to give the reason for withdrawal. The participant will be allowed sufficient time (a minimum of 24 hours) to consider the information and decide whether to take part before consent is sought. Written informed consent will then be obtained by means of patient dated signature and dated signature of the person who presented and obtained the informed consent.

Patients wishing to withdraw from the study will not be replaced. If a participant decides to withdraw, their coded blood samples and information will be retained for use within REQUITE and for future medical research unless the patient makes a specific request otherwise. If specifically requested (either verbally or in writing to a member of the research team), the blood samples will be destroyed and the medical information removed from the REQUITE database.

### 14.4.3 Management and oversight arrangements

This sub-study will be overseen by the main REQUITE Study Management Group (SMG), as the patients in this sub-study will come from the main study or REQUITE-AB sub-study. The conduct of this sub-study will be monitored as part of the arrangements for the main study.

The University of Manchester will remain as sponsor for this sub-study. The contact point is:

Professor Nalin Thakker

Associate Vice-President (Research Integrity)

The University of Manchester

Oxford Road, Manchester M13 9PL

Email: [research-governance@manchester.ac.uk](mailto:research-governance@manchester.ac.uk)

Telephone: 0161 275 8795

The University of Manchester has insurance for research involving human subjects that provides cover for legal liabilities arising from its actions or those of its staff, subject to policy terms and conditions. For participating sites which are part of the NHS, the NHS indemnity scheme will also apply.

### 14.4.4 Sub-study publication

For the main publication(s) arising from this sub-study, it is anticipated that all contributors will be authors. The main results of the REQUITE-AB-QOL sub-study will be published in a peer-reviewed journal. The manuscript will be prepared by a writing group, consisting of researchers and investigators, and, where appropriate, REQUITE SMG members of participating centres as per publications policy.

A formal publications policy due to be generated by the REQUITE SMG, which all participants will be asked to sign up to. All presentations and publications relating to the sub-study will require authorisation by the SMG.

# 15.0 SUB-STUDY 3: REQUITE–CHRONO

## 15.1 Summary

| **TITLE** | REQUITE-CHRONO: Circadian rhythm REQUITE sub-study |
| --- | --- |
| **STUDY DESIGN** | Questionnaire study gaining further information from REQUITE breast and prostate cancer patients recruited into the main study |
| **TARGET DISEASE** | Cancer of the breast and prostate |
| **PRIMARY STUDY OBJECTIVE** | To establish if there is a link between chronotype (morning-evening preference) and radiotherapy toxicity. |
| **SECONDARY STUDY OBJECTIVE** | To establish if there is a link with chronotype and circadian rhythm genes. |
| **PARTICIPATING SITE** | University of Leicester |
| **STUDY POPULATION** | Breast and prostate cancer patients recruited into the main REQUITE study |
| **RECRUITMENT TARGET** | 350 breast and 250 prostate patients will be sent the questionnaires. 50% return rate is expected. |
| **FOLLOW UP VISITS** | No |
| **STUDY DURATION** | Patients to be recruited over the duration of the main REQUITE study |
| **SAMPLE COLLECTION** | Munich Chronotype Questionnaire |
| **POWER CALCULATIONS** | In our small pilot study we observed a significant protective effect if evening chronotype patients were treated in the afternoon. In total, in this pilot study, 28% of patients received their radiotherapy in the afternoon. None of the patients with evening chronotype experienced late toxicity however 47% of patients in the normal or early chronotype group did. Assuming 50% response rate (n=300) then the study would be powered to 98.0% using the same expected values as the pilot study. |

**15.2 Background**

### 15.2.1 Circadian Rhythm

Circadian rhythm is controlled by clock genes which cause diurnal variations in proteins CRY, PER, CLOCK and BMAL^[[58]](#footnote-58)^ . Barnett et al, 2014 ^[[59]](#footnote-59)^ showed an association between late radiotherapy toxicity and a SNP (rs13116075) close to the CCRN4L gene (for the protein nocturnin). CCRN4L is a gene controlled by circadian rhythm and is linked to metabolism of lipids ^[[60]](#footnote-60)^. Levels of this protein undergo diurnal variation with peaks at night. It has also been linked to inflammation^[[61]](#footnote-61)^ which may be the possible mechanism for increasing radiotherapy toxicity. Clock genes/proteins then in turn exert effects on other proteins which have a wide physiological effect. This includes DNA damage checkpoints and apoptosis mechanisms which govern repair in normal tissues following radiotherapy. Variations in these clock genes can influence a person’s sleep wake cycle^[[62]](#footnote-62)^ and ability to repair DNA damage^[[63]](#footnote-63)^ .

### 15.2.2 Rationale for the study

We hypothesise that radiotherapy toxicity is affected by variation in clock genes. In turn, timing of radiotherapy would also affect whether patients develop side-effects depending on their clock genotype. Using data already collected from a pilot cohort we have observed a significant increase in late radiotherapy reaction in patients with a particular clock genotype (PER 3 and CCRN4L) when these patients were treated in the mornings. We have also observed that patients in the pilot cohort with a particular chronotype (assessed using the Munich Chronotype questionnaire) correlate with risk of radiotherapy toxicity. This questionnaire has been completed by the pilot cohort and has been used extensively in many other studies. This questionnaire was developed as part of an EU-funded chronobiology grant and has been validated in more than 50,000 volunteers so far.

## 15.3 Sub-study research plan

### 15.3.1 Study objectives and design

The main objective of the Circadian Rhythm REQUITE sub-study is to validate our findings from the pilot study that circadian rhythm can be used to predict a patient’s risk of developing radiotherapy toxicity.

Secondary objectives include establishing if variations in chronotype relate to polymorphisms in circadian rhythm genes. The main part of this sub-study will be questionnaire based.

### 15.3.2 Patient selection and recruitment

All breast and prostate cancer patients recruited to the main REQUITE study at the University of Leicester will be approached to take part in this sub-study. This will give a maximum sample size of 600. However, it is anticipated that there will be a 50% return rate giving 300 participants. It is expected that the sample will include adequate representation of age, ethnicity, disease factors, co-morbidities (e.g. diabetes or anxiety/depression) and degrees of toxicity.

Patients will be consented for the questionnaire sub-study using an additional consent form and additional patient information sheet.

### 15.3.3 Study methodology and data analysis

Selected participants will be sent the Munich chronotype questionnaire to collect information on their sleep wake cycle. A patient invite letter, patient information sheet and consent form will be sent alongside the questionnaire, and patients will be asked to sign and return the consent form along with the completed questionnaire in a pre-paid envelope that is provided. The consent form will be counter-signed on receipt and a copy returned to the patient. Data from the completed questionnaire will be analysed using an algorithm created by the University of Munich to generate chronotype for each participant.

Genotyping for circadian rhythm genes will be undertaken at the University of Leicester using DNA isolated from EDTA blood samples previously collected as part of the REQUITE study (sample A). This will then be correlated with data from the chronotype and radiotherapy toxicity to perform further analysis.

## 15.4 Sub-study research arrangements

### 15.4.1 Compliance and data protection

All investigators and researchers involved with the study must comply with the national requirements for data protection with regard to the collection, storage, processing and disclosure of personal information and agree to uphold the appropriate core principles.

Patient notes and study files at site must be kept in a secure storage area with limited access.

Any access required to the REQUITE database will be strictly limited via usernames and passwords. Published results will not contain any personal data that could allow identification of individual patients.

### 15.4.2 Ethical and regulatory requirements

The study will be conducted in accordance with the approved protocol, the Declaration of Helsinki, ICH Guidelines for Good Clinical Practice (ICH GCP), relevant regulations and standard operating procedures.

Research Ethics approval for this sub-study will be sought from the NRES Committee North West – Greater Manchester East by way of major amendment to the previously approved protocol (ref. 14/NW/0035). Permission to conduct the sub-study will be sought from the R&D department at participating centres.

The local Principal Investigator (PI) must ensure that the study protocol, patient information sheet, consent form, family doctor letter and submitted supporting documents have been approved by the appropriate regulatory bodies and research ethics committee(s) prior to any patient recruitment.

Any agreed substantial amendments must also be submitted and receive ethical and regulatory approval prior to implementation. It is the responsibility of the PI at each site to ensure that the study has all the necessary approvals in place. A site initiation meeting must be completed prior to each study centre opening to recruitment.

Patients will be recruited onto the sub-study using a separate Circadian Rhythm REQUITE sub-study patient information sheet and separate consent form. A patient information sheet will be presented to potential participants detailing the nature of the study, the implications of participating and any potential risks or inconveniences involved in taking part. It will be clearly stated that the participant is free to withdraw from the study at any time, for any reason, without prejudice to future care and with no obligation to give the reason for withdrawal. The participant will be allowed sufficient time (a minimum of 24 hours) to consider the information and decide whether to take part before consent is sought. Written informed consent will then be obtained by means of patient dated signature and dated signature of the person who presented and obtained the informed consent.

Patients wishing to withdraw from the study will not be replaced. If a participant decides to withdraw, their coded blood samples and information will be retained for use within REQUITE and for future medical research unless the patient makes a specific request otherwise. If specifically requested (either verbally or in writing to a member of the research team), the blood samples will be destroyed and the medical information removed from the REQUITE database.

### 15.4.3 Management and oversight arrangements

This sub-study will be overseen by the main REQUITE Study Management Group (SMG), as the patients in this sub-study will come from the main study. The conduct of this sub-study will be monitored as part of the arrangements for the main study.

The University of Manchester will remain as sponsor for this sub-study. The contact point is:

Professor Nalin Thakker

Associate Vice-President (Research Integrity)

The University of Manchester

Oxford Road, Manchester M13 9PL

Email: [research-governance@manchester.ac.uk](mailto:research-governance@manchester.ac.uk)

Telephone: 0161 275 8795

The University of Manchester has insurance for research involving human subjects that provides cover for legal liabilities arising from its actions or those of its staff, subject to policy terms and conditions. For participating sites which are part of the NHS, the NHS indemnity scheme will also apply.

### 15.4.4 Sub-study publication

For the main publication(s) arising from this sub-study, it is anticipated that all contributors will be authors. The main results of the Circadian Rhythm REQUITE sub-study will be published in a peer-reviewed journal. The manuscript will be prepared by a writing group, consisting of researchers and investigators, and, where appropriate, REQUITE SMG members of participating centres as per the publications policy.

A formal publications policy due to be generated by the REQUITE SMG, which all participants will be asked to sign up to. All presentations and publications relating to the sub-study will require authorisation by the SMG.

Barnett, G. C., D. Thompson, L. Fachal, S. Kerns, C. Talbot, R. M. Elliott, L. Dorling, C. E. Coles, D. P. Dearnaley, B. S. Rosenstein, A. Vega, P. Symonds, J. Yarnold, C. Baynes, K. Michailidou, J. Dennis, J. P. Tyrer, J. S. Wilkinson, A. Gomez-Caamano, G. A. Tanteles, R. Platte, R. Mayes, D. Conroy, M. Maranian, C. Luccarini, S. L. Gulliford, M. R. Sydes, E. Hall, J. Haviland, V. Misra, J. Titley, S. M. Bentzen, P. D. Pharoah, N. G. Burnet, A. M. Dunning, and C. M. West. "A Genome Wide Association Study (Gwas) Providing Evidence of an Association between Common Genetic Variants and Late Radiotherapy Toxicity." *Radiother Oncol* 111, no. 2 (2014): 178-85.

Douris, N., S. Kojima, X. Pan, A. F. Lerch-Gaggl, S. Q. Duong, M. M. Hussain, and C. B. Green. "Nocturnin Regulates Circadian Trafficking of Dietary Lipid in Intestinal Enterocytes." *Curr Biol* 21, no. 16 (2011): 1347-55.

Gomes, A. M., R. C. Barber, and Y. E. Dubrova. "Paternal Irradiation Perturbs the Expression of Circadian Genes in Offspring." *Mutat Res* 775, (2015): 33-7.

Sancar, A., L. A. Lindsey-Boltz, S. Gaddameedhi, C. P. Selby, R. Ye, Y. Y. Chiou, M. G. Kemp, J. Hu, J. H. Lee, and N. Ozturk. "Circadian Clock, Cancer, and Chemotherapy." *Biochemistry* 54, no. 2 (2015): 110-23.

Stubblefield, J. J., J. Terrien, and C. B. Green. "Nocturnin: At the Crossroads of Clocks and Metabolism." *Trends Endocrinol Metab* 23, no. 7 (2012): 326-33.

von Schantz, M. "Phenotypic Effects of Genetic Variability in Human Clock Genes on Circadian and Sleep Parameters." *J Genet* 87, no. 5 (2008): 513-9.

# 16.0 SUB-STUDY 4: REQUITE-BP (Breast Pain) Sub-study

## 16.1 Summary

| **TITLE** | REQUITE - Predictors of long-term breast pain after breast cancer treatment |
| --- | --- |
| **STUDY DESIGN** | A study embedded within the REQUITE main study administering pain questionnaires to a sample of breast cancer patients recruited into the main study to evaluate and better understand chronic, long-term breast pain by incorporating pain sensitivity |
| **TARGET DISEASE** | Cancer of the breast |
| **PRIMARY STUDY OBJECTIVE** | To identify clinical and biological factors that are predictive of long-term breast pain |
| **SECONDARY STUDY OBJECTIVE** | To develop a prediction model for long-term breast pain (> 2 years) after breast cancer treatment |
| **PARTICIPATING SITES** | University of Leicester |
| **STUDY POPULATION** | Breast cancer patients recruited into the main REQUITE study |
| **RECRUITMENT TARGET** | Sample size expected to be 50% of REQUITE patient cohort (n=350), approximately 175 patients. |
| **FOLLOW UP VISITS** | Single administration of questionnaires at follow-up visit or by post |
| **STUDY DURATION** | Patients to be recruited during long-term follow-up of the main REQUITE study |
| **SAMPLE COLLECTION** | Pain assessment questionnaires: Pain Sensitivity questionnaire^[[64]](#footnote-64)^, McGill pain questionnaire^[[65]](#footnote-65)^, West Haven-Yale Multi-dimensional Pain Inventory (REF) ^66^, Hospital Anxiety and Depression scale (HADs)^67^ and Pain Catastrophizing scale^68^. |
| **POWER CALCULATIONS** | We expect around 50% of the REQUITE patients to consent to enter this study, giving questionnaire data on 175 patients. In terms of genotyping, this cohort size will allow 80% power at α=0.05 to detect an Odds Ratio >1.42 given realistic parameters (prevalence 0.44, allele frequency 0.2, D’ 0.8) |

## 16.2 Background

### 16.2.1 Chronic breast-pain following breast cancer treatment

Breast cancer is the most frequently diagnosed cancer in women^69^. The prevalence of chronic pain in breast cancer patients ranges from 25-60% within different populations, but it tends to be underreported by survivors^69^. Research has shown that chronic pain has an association with reduction in quality of life, affecting work re-entry, productivity and an individual’s identity^71,72^

Patients regard the risk of long-term breast pain as an important factor in treatment decision-making^73^. Owing to improvements in diagnosis and treatment, there are an increasing number of long-term breast cancer survivors living with late side-effects of treatment such as chronic breast-pain^71,74^. The causes associated with certain types of pain after breast cancer treatment have been clearly identified, for example, peripheral neuropathy associated with chemotherapy, or bone pain with aromatase inhibitors^75,76^. However, the underlying causes and mechanism of long-term breast pain in breast cancer survivors remain unclear.

Studies of breast cancer survivors have shown that chronic breast pain generally resolves with time^72,77,78^. Nevertheless, these studies have not considered other factors, such as ethnicity and age. In terms of ethnicity, studies in different clinical chronic-pain conditions have shown that pain perception can vary owing to differences in sociocultural and neurobiological processes^79-84^. In older patients, the increased incidence in chronic breast pain has been attributed to the more aggressive nature of cancer, use of chemotherapy and links to anxiety and depression^85,86^. Our understanding of the interaction between the protective effect of time and other patient clinical or biological factors is limited.

Pain perception is subjective, with many neurocognitive aspects modulating the end pain experience87. Anxiety has been linked with the emotional response to pain, however, the exact effect of anxiety on pain perception remains ambiguous^88-90^. Within breast cancer survivors, patients with anxiety and depression are more likely to report long-term pain post-breast cancer treatment with a higher percentage being younger women^70,91,92^.

^68^ Sullivan MJL, Bishop SR, Pivik J. The pain Catatrophising Scale: Development and validation. Psychological assessment, 1995; 7: 524-532

69 Bray F, Ferlay J, Soerjomataram I, Siegel RL, Torre LA, Jemal A. Global cancer statistics 2018: GLOBOCAN estimates of incidence and mortality worldwide for 36 cancers in 185 countries. 2018 Sep 12;

70 Schou Bredal I, Smeby NA, Ottesen S, Warncke T, Schlichting E. Chronic Pain in Breast Cancer Survivors: Comparison of Psychosocial, Surgical, and Medical Characteristics Between Survivors With and Without Pain. 2014;48(5):852–62.

^71^ Hamood R, Hamood H, Merhasin I, Keinan-Boker L. Chronic pain and other symptoms among breast cancer survivors: prevalence, predictors, and effects on quality of life. 2018;167(1):157–69

^72^ Tasmuth T, Von Smitten K, Hietanen P, Kataja M, Kalso E. Pain and other symptoms after different treatment modalities of breast cancer. 1995;6(5):453.

73 Rattay T, Symonds RP, Shokuhi S, Talbot CJ, Schnur JB. The Patient Perspective on Radiogenomics Testing for Breast Radiation Toxicity. 2018;

74 Miller KD, Siegel RL, Lin CC, Mariotto AB, Kramer JL, Rowland JH, et al. Cancer treatment and survivorship statistics, 2016. 2016 Jul;66(4):271–89.

^75^ Hausheer, F.H., Schilsky, R.L., Bain, S., Berghorn, E.J. and Lieberman, F. (2006) 'Diagnosis, management, and evaluation of chemotherapy-induced peripheral neuropathy', *Seminars in oncology,*33(1), pp. 15-49.

^76^ Henry, N.L., Giles, J.T., Ang, D., Mohan, M., Dadabhoy, D., Robarge, J., Hayden, J., Lemler, S., Shahverdi, K., Powers, P., Li, L., Flockhart, D., Stearns, V., Hayes, D.F., Storniolo, A.M. and Clauw, D.J. (2008) 'Prospective characterization of musculoskeletal symptoms in early stage breast cancer patients treated with aromatase inhibitors', *Breast cancer research and treatment,*111(2), pp. 365-372.

77 Poleshuck EL, Katz J, Andrus CH, Hogan LA, Jung BF, Kulick DI, et al. Risk Factors for Chronic Pain Following Breast Cancer Surgery: A Prospective Study. 2006;7(9):626–34.

^78^ Andersen K, Gartner R, Kroman N, Flyger H, Kehlet H. Persistent Pain After Targeted Intraoperative Radiotherapy (TARGIT) or External Breast Radiotherapy for Breast Cancer - a Randomized Trial. 2011;47:S388.

^79^ Breitbart, W., Rosenfeld, B.D., Passik, S.D., McDonald, M.V., Thaler, H. and Portenoy, R.K. (1996) 'The undertreatment of pain in ambulatory AIDS patients', *Pain,*65(2-3), pp. 243-249.

^80^ Creamer, P., Lethbridge-Cejku, M. and Hochberg, M.C. (1999) 'Determinants of pain severity in knee osteoarthritis: effect of demographic and psychosocial variables using 3 pain measures', *The Journal of rheumatology,*26(8), pp. 1785-1792

^81^ Edwards, R.R., Doleys, D.M., Fillingim, R.B. and Lowery, D. (2001) 'Ethnic differences in pain tolerance: clinical implications in a chronic pain population', *Psychosomatic medicine,*63(2), pp. 316-323.

^82^ Sheffield, D., Biles, P.L., Orom, H., Maixner, W. and Sheps, D.S. (2000) 'Race and sex differences in cutaneous pain perception', *Psychosomatic medicine,*62(4), pp. 517-523

83 Sherwood, M.B., Garcia-Siekavizza, A., Meltzer, M.I., Hebert, A., Burns, A.F. and McGorray, S. (1998) 'Glaucoma's impact on quality of life and its relation to clinical indicators. A pilot study', *Ophthalmology,*105(3), pp. 561-566

84 Wandner, L.D., Scipio, C.D., Hirsh, A.T., Torres, C.A. and Robinson, M.E. (2012) 'The perception of pain in others: how gender, race, and age influence pain expectations', *The journal of pain : official journal of the American Pain Society,*13(3), pp. 220-227.

^85^Avis, N., Levine, B., Naughton, M., Case, D., Naftalis, E. and Zee, K. (2012) 'Explaining age-related differences in depression following breast cancer diagnosis and treatment', *Breast cancer research and treatment,*136(2), pp. 581-591.

^86^ Kroman, N., Jensen, M., Wohlfahrt, J., Mouridsen, H.T., Andersen, P.K., Melbye, M., Tutt, A. and Ross, G. (2000) 'Factors influencing the effect of age on prognosis in breast cancer: population based study', *British medical journal,*320(7233), pp. 474.

^87^ Wiech, K., Ploner, M. and Tracey, I. (2008) 'Neurocognitive aspects of pain perception', *Trends in cognitive sciences,*12(8), pp. 306-313.

88 Bobey, M.J. and Davidson, P.O. (1970) 'Psychological factors affecting pain tolerance', *Journal of psychosomatic research,*14(4), pp. 371-376.

^89^ Cornwall, A. and Donderi, D.C. (1988) 'The effect of experimentally induced anxiety on the experience of pressure pain', *Pain,*35(1), pp. 105-113.

90 Weisenberg, M. (ed.) (1987) *Psychological intervention for the control of pain.*

^91^ Sipila R, Estlander A-M, Tasmuth T, Kataja M, Kalso E. Development of a screening instrument for risk factors of persistent pain after breast cancer surgery. 2012 Oct 23;107(9):1459–66.

^92^ Parks J, Yang X, Zhang C, Chen Z, Miller AH, Godette KD, et al. Predictors of Breast Pain in Breast Cancer Patents One Year After Whole Breast Radiation Therapy [Internet]. 2017. p. E41. (International Journal of Radiation Oncology*Biology*Physics; vol. 99).

Pain catastrophising describes an adverse mental state in an individual experiencing actual or impending pain^93.^. Pain frequency is thought to play a pivotal role in catastrophizing pain. A higher frequency of pain has been shown to activate supra-spinal pathways, leading to further attention and vigilance of pain^94-96^. Some studies in breast cancer pain show a relationship between pre-surgical pain and post-surgical reported pain. It is postulated that this could stem from body hypervigilance and attention leading to somatisation and transition from acute to chronic pain^97-98^.

The precise impact of treatment side-effects (toxicity) on chronic breast pain in breast cancer survivors remains unclear, but factors are thought to include intercosto-brachial nerve damage, radiotherapy and some types of chemotherapy agents^72^. The nature of chronic breast pain may be related to the type of treatment side-effect. Surgical procedures (e.g. axillary lymph node dissection) which have a higher risk of intercosto-brachial and sensory nerve damage may lead to the development of long-term neuropathic pain, but this association remains inconclusive^98-100^. Other factors such as the presence of lymphoedema post-axillary lymph node dissection or axillary radiotherapy may also be associated^69^.

Radiation treatment in itself may be an independent risk factor in the development of long-term breast pain^78,100^, although the association may not be significant on multivariate analysis^101^. So far, no association has been shown between fractionation schedule, radiation dose, and reported pain92^,100^. Nevertheless, certain treatment combinations, such as chemotherapy followed by radiotherapy and axillary dissection may increase the risk of radiation plexopathy and thus pain^78,100^.

### 16.2.2 Rationale for the study

There is limited understanding on what causes long-term breast pain in breast cancer survivors. Chronic breast pain remains a major concern, with many patients not being aware of this being a consequence of treatment and its potential effect on quality of life (QoL). Research is needed to understand the predictive factors associated with long-term breast pain, which in the future may help to tailor treatment to reduce the impact of chronic breast pain on QoL and inform the development of pain management strategies to improve overall QoL.

93 Quartana, P.J., Campbell, C.M. and Edwards, R.R. (2009) 'Pain catastrophizing: a critical review', *Expert review of neurotherapeutics,*9(5), pp. 745-758.

^94^ Bezov, D., Ashina, S., Jensen, R. and Bendtsen, L. (2011) 'Pain perception studies in tension-type headache', *Headache,*51(2), pp. 262-271.

^95^ Coghill, R.C., McHaffie, J.G. and Yen, Y.F. (2003) 'Neural correlates of interindividual differences in the subjective experience of pain', *Proceedings of the National Academy of Sciences of the United States of America,*100(14), pp. 8538-8542.

96Kudel I, Edwards RR, Kozachik S, Block BM, Agarwal S, Heinberg LJ, et al. Predictors and Consequences of Multiple Persistent Postmastectomy Pains. 2007;34(6):619–27.

97Barsky AJ, Peekna HM, Borus JF. Somatic Symptom Reporting in Women and Men. Boston, MA, USA; 2001. p. 266–75. (Journal of General Internal Medicine; vol. 16).

98C. V J., Brand VD, O. W JM. Post-axillary dissection pain in breast cancer due to a lesion of the intercostobrachial nerve. 1989;38(2):171–6

99Ivens D, Hoe A, Podd T, Hamilton C, Taylor I, Royle G. Assessment of morbidity from complete axillary dissection. 1992;66(1):136.

100Lundstedt D, Gustafsson M, Steineck G, Malmstrom P, Alsadius D, Sundberg A, et al. Risk Factors of Developing Long-Lasting Breast Pain After Breast Cancer Radiotherapy. 2012;83(1):71–8.

^101^ Juhl, A.A., Christiansen, P. and Damsgaard, T.E. (2016) 'Persistent Pain after Breast Cancer Treatment: A Questionnaire-Based Study on the Prevalence, Associated Treatment Variables, and Pain Type', *Journal of breast cancer,*19(4), pp. 447-454.

## 16.3 Sub-study research plan

### 16.3.1 Study objectives and design

The main objective of the REQUITE-BP sub-study is to explore the association of chronic breast pain with clinical and biological factors of breast cancer treatment, including treatment toxicity, with the aim of identifying predictive factors for chronic breast pain following surgery and radiotherapy for breast cancer.

The study has been designed as an additional sub-study to the main REQUITE study protocol.

### 16.3.2 Patient selection and recruitment

Patients taking part in the main REQUITE study at the University of Leicester will be contacted with additional pain questionnaires^64-67^ and the Pain Catastrophizing scale^68^ through post or face-to-face contact at follow-up clinics. Patients will be given/ sent a cover letter along with the additional questionnaires. They will be consented for the sub-study using one additional consent form,. If participating by post, consent forms will be countersigned on receipt.

From an original sample size of 350 treated by breast-conserving surgery and adjuvant radiotherapy, we expect a 50% return rate, yielding 175 participants. It is expected that the sample population will be representative of age, ethnicity, presence vs. absence of chemotherapy, presence vs. absence of hormonal therapies, use of painkillers, co-morbidities, and stage of disease.

### 16.3.3 Study methodology and data analysis

In order to assess patients’ perspective of chronic breast pain we will administer and collect questionnaire data on pain and pain sensitivity either face-to-face in the clinic or by post. We will include five questionnaires (Pain Sensitivity questionnaire^64^, McGill pain questionnaire^65^, West Haven-Yale Multi-dimensional Pain Inventory (REF)^66^, Hospital Anxiety and Depression Scale (HADS)^67^, and the Pain Catastrophizing scale^68^. The inclusion of the Pain Sensitivity^64^ questionnaires is to collect data to account for confounders of pain perception that are not already present in the REQUITE data.

Pain questionnaires will be sent out with a cover letter, consent form and patient information sheet attached. Postal questionnaires will have a pre-paid envelope enclosed to return questionnaires and the consent form. Each questionnaire will be identified using the patient’s unique REQUITE ID so data can be matched with the REQUITE database, but all other aspects are anonymised.

We will investigate the association of long-term breast pain at 2 years recorded in the main study using the EORCTQLQ-C30 and –BR23^102^ tools by integrating the additional questionnaire data with predictive variables from the REQUITE database, including but not limited to treatment details, co-morbidities, toxicity and other PROMs data using multivariate statistical analysis. Whole genome SNP data will be also be incorporated.

^102^ Sprangers MAG, Groenvold M, Arraras JI, et al.. The European Organisation for Research and Treatment of Cancer: Breast Cancer Specific Quality of Life Questionnaire Module: First results from a three-country field study. J. Clin. Oncol

14:2756-2768, 1996.

## 16.4 Sub-study-research arrangements

### 16.4.1 Compliance and data protection

All investigators and researchers involved with the study must comply with the national requirements for data protection with regard to the collection, storage, processing and disclosure of personal information and agree to uphold the appropriate core principles.

Patient notes and study files at site must be kept in a secure storage area with limited access. Any access required to the REQUITE database will be strictly limited via usernames and passwords. Published results will not contain any personal data that could allow identification of individual patients.

### 16.4.2 Ethical and regulatory requirements

The study will be conducted in accordance with the approved protocol, the Declaration of Helsinki, ICH Guidelines for Good Clinical Practice (ICH GCP), relevant regulations and standard operating procedures.

Research Ethics approval for this sub-study will be sought from the NRES Committee North West – Greater Manchester East by way of major amendment to the previously approved protocol (ref. 14/NW/0035). Permission to conduct the sub-study will be sought from the R&D department at participating centres.

The local Principal Investigator (PI) must ensure that the study protocol, patient information sheet, consent form, family doctor letter and submitted supporting documents have been approved by the appropriate regulatory bodies and research ethics committee(s) prior to any patient recruitment.

Any agreed substantial amendments must also be submitted and receive ethical and regulatory approval prior to implementation. It is the responsibility of the PI at each site to ensure that the study has all the necessary approvals in place. A site initiation meeting must be completed prior to each study centre opening to recruitment.

Patients will be recruited onto the sub-study using a separate REQUITE-BP patient information sheet and consent form. Potential participants will be approached in clinic face-to-face or by post with a cover letter, detailing the nature of the study, the implications of participating and any potential risks or inconveniences involved in taking part. It will be clearly stated that the participant is free to withdraw from the study at any time, for any reason, without prejudice to future care and with no obligation to give the reason for withdrawal. Written informed consent will then be obtained by means of patient dated signature and dated signature of the person who presented and obtained the informed consent. For patients submitting their questionnaires by post, consent forms will be countersigned on receipt

Patients wishing to withdraw from the study will not be replaced. If a participant decides to withdraw, their information will be retained for use within REQUITE and for future medical research unless the patient makes a specific request otherwise. If specifically requested (either verbally or in writing to a member of the research team), the medical information will be removed from the REQUITE database.

### 16.4.3 Management and oversight of arrangements

This sub-study will be overseen by the main REQUITE Study Management Group (SMG), as the patients in this sub-study will come from the main study. The conduct of this sub-study will be monitored as part of the arrangements for the main study.

The University of Manchester will remain as sponsor for this sub-study. The contact point is:

Professor Nalin Thakker

Associate Vice-President (Research Integrity)

The University of Manchester

Oxford Road, Manchester M13 9PL

Email: [research-governance@manchester.ac.uk](mailto:research-governance@manchester.ac.uk)

Telephone: 0161 275 8795

The University of Manchester has insurance for research involving human subjects that provides cover for legal liabilities arising from its actions or those of its staff, subject to policy terms and conditions. For participating sites which are part of the NHS, the NHS indemnity scheme will also apply.

### 16.4.4 Sub-Study publication

For the main publication(s) arising from this sub-study, it is anticipated that all contributors will be authors. The results of the REQUITE-BP sub-study will be published in a peer-reviewed journal. The manuscript will be prepared by a writing group, consisting of researchers and investigators, and, where appropriate, REQUITE SMG members of participating centres as per publications policy.

A formal publications policy due to be generated by the REQUITE SMG, which all participants will be asked to sign up to. All presentations and publications relating to the sub-study will require authorisation by the SMG.

1. http://www.iarc.fr/en/media-centre/iarcnews/2011/globocan2008-prev.php [↑](#footnote-ref-1)
2. Jeremić B, Miličić B, Milisavljević S. Radiotherapy Alone vs. Radiochemotherapy in Patients With Favorable Prognosis of Clinical Stage IIIA Non-Small-Cell Lung Cancer. Clin Lung Cancer 2013 epub Jan 4. [↑](#footnote-ref-2)
3. Bentzen SM, Heeren G, Cottier B, et al. Towards evidence-based guidelines for radiotherapy infrastructure and staffing needs in Europe: the ESTRO QUARTS project. Radiother Oncol 2005;75:355-65 [↑](#footnote-ref-3)
4. Al-Ghazal SK, Fallowfield L, Blamey RW. Does cosmetic outcome from treatment of primary breast cancer influence psychosocial morbidity? Eur J Surg Oncol 1999;25:571-3. [↑](#footnote-ref-4)
5. Barker CL, Routledge JA, Farnell DJ, Swindell R, Davidson SE. The impact of radiotherapy late effects on quality of life in gynaecological cancer patients.Br J Cancer 2009;100:1558-65. [↑](#footnote-ref-5)
6. Sanda MG, Dunn RL, Michalski J et al. Quality of life and satisfaction with outcome among prostate-cancer survivors. N Engl J Med. 2008 Mar 20;358(12). [↑](#footnote-ref-6)
7. Harden JK, Sanda MG, Wei JT et al Partners' long-term appraisal of their caregiving experience, marital satisfaction, sexual satisfaction, and quality of life 2 years after prostate cancer treatment. Cancer Nurs. 2013 Mar-Apr;36(2) [↑](#footnote-ref-7)
8. Crompton NE, Miralbell R, et al, Ozsahin M. Altered apoptotic profiles in irradiated patients with increased toxicity. Int J Radiat Oncol Biol Phys 1999;45:707-14. [↑](#footnote-ref-8)
9. Barber JB, West CM, Kiltie AE, et al. Detection of individual differences in radiation-induced apoptosis of peripheral blood lymphocytes in normal individuals, ataxia telangiectasia homozygotes and heterozygotes, and breast cancer patients after radiotherapy. Radiat Res 2000;153:570-8. [↑](#footnote-ref-9)
10. Crompton NE, Shi YQ, et al, Ozsahin M. Sources of variation in patient response to radiation treatment. Int J Radiat Oncol Biol Phys 2001;49:547-54. [↑](#footnote-ref-10)
11. Ozsahin M, Crompton NE, et al, Azria D. CD4 and CD8 T-lymphocyte apoptosis can predict radiation-induced late toxicity: a prospective study in 399 patients. Clin Cancer Res 2005;11:7426-33. [↑](#footnote-ref-11)
12. Schnarr K, Boreham D, Sathya J, Julian J, Dayes IS. Radiation-induced lymphocyte apoptosis to predict radiation therapy late toxicity in prostate cancer patients. Int J Radiat Oncol Biol Phys 2009;74:1424-30. [↑](#footnote-ref-12)
13. Bordón E, Henríquez-Hernández LA, Lara PC, et al. Prediction of clinical toxicity in locally advanced head and neck cancer patients by radio-induced apoptosis in peripheral blood lymphocytes (PBLs). Radiat Oncol 2010;5:4 [↑](#footnote-ref-13)
14. Bordón E, Henríquez-Hernández LA, Lara PC, et al. Role of CD4 and CD8 T-lymphocytes, B-lymphocytes and Natural Killer cells in the prediction of radiation-induced late toxicity in cervical cancer patients. Int J Radiat Biol 2011;87:424-31. [↑](#footnote-ref-14)
15. West CM, Barnett GC. Genetics and genomics of radiotherapy toxicity: towards prediction. Genome Med 2011;3:52. [↑](#footnote-ref-15)
16. Barnett GC, Coles CE, Elliott RM, et al, Bentzen SM, Dearnaley DP, Burnet NG, Pharoah PD, Dunning AM, West CM. Independent validation of genes and polymorphisms reported to be associated with radiation toxicity: a prospective analysis study. Lancet Oncol 2012;13:65-77. [↑](#footnote-ref-16)
17. Fachal L, Gómez-Caamaño A, Sánchez-García M, Carballo A, Peleteiro P, Lobato-Busto R, Carracedo A, Vega A. TGFβ1 SNPs and radio-induced toxicity in prostate cancer patients. Radiother Oncol 2012;103:206-9. [↑](#footnote-ref-17)
18. Barnett GC, Elliott RM, Alsner J, et al. Individual patient data meta-analysis shows no association between the SNP rs1800469 in TGFB and late radiotherapy toxicity. Radiother Oncol 2012;105:289-95. [↑](#footnote-ref-18)
19. Talbot CJ, Tanteles GA, Barnett GC, et al. A replicated association between polymorphisms near TNFα and risk for adverse reactions to radiotherapy. Br J Cancer 2012;107:748-53. [↑](#footnote-ref-19)
20. Kerns SL, Ostrer H, et al, Rosenstein BS. Genome-wide association study to identify single nucleotide polymorphisms (SNPs) associated with the development of erectile dysfunction in African-American men after radiotherapy for prostate cancer. Int J Radiat Oncol Biol Phys 2010;78:1292-300. [↑](#footnote-ref-20)
21. Kerns SL, et al, Ostrer H, Rosenstein BS. A 2-stage genome-wide association study to identify single nucleotide polymorphisms associated with development of erectile dysfunction following radiation therapy for prostate cancer. Int J Radiat Oncol Biol Phys 2013;85:e21-8. [↑](#footnote-ref-21)
22. De Ruyck K, Sabbe N, Oberije C, Vandecasteele K, Thas O, De Ruysscher D, Lambin P, Van Meerbeeck J, De Neve W, Thierens H. Development of a multicomponent prediction model for acute esophagitis in lung cancer patients receiving chemoradiotherapy. Int J Radiat Oncol Biol Phys 2011;81:537-44. [↑](#footnote-ref-22)
23. Tucker SL, Li M, Xu T, et al. Incorporating Single-nucleotide Polymorphisms Into the Lyman Model to Improve Prediction of Radiation Pneumonitis. Int J Radiat Oncol Biol Phys 2013;85:251-7. [↑](#footnote-ref-23)
24. Yarnold J, Ashton A, Bliss J, et al. Fractionation sensitivity and dose response of late adverse effects in the breast after radiotherapy for early breast cancer: long-term results of a randomised trial. Radiother Oncol 2005;75:9-17. [↑](#footnote-ref-24)
25. Fellin G, Fiorino C, et al, Valdagni R. Clinical and dosimetric predictors of late rectal toxicity after conformal radiation for localized prostate cancer: results of a large multicenter observational study. Radiother Oncol 2009;93:197-202. [↑](#footnote-ref-25)
26. Marks LB, Bentzen SM, Deasy JO, et al. Radiation dose-volume effects in the lung. Int J Radiat Oncol Biol Phys 2010;76:S70-6. [↑](#footnote-ref-26)
27. Graham MV, Purdy JA, Emami B, et al. Clinical dose-volume histogram analysis for pneumonitis after 3D treatment for non-small cell lung cancer (NSCLC). Int J Radiat Oncol Biol Phys 1999;45:323-9. [↑](#footnote-ref-27)
28. Trotti A, Bentzen SM. The need for adverse effects reporting standards in oncology clinical trials. J Clin Oncol 2004;22:19-22. [↑](#footnote-ref-28)
29. Farnell DJ, et al, West CM, Davidson SE. Development of a patient-reported questionnaire for collecting toxicity data following prostate brachytherapy. Radiother Oncol 2010;97:136-42. [↑](#footnote-ref-29)
30. Ho KF, Farnell DJ, Routledge JA, Burns MP, Sykes AJ, Slevin NJ, Davidson SE. Comparison of patient-reported late treatment toxicity (LENT-SOMA) with quality of life (EORTC QLQ-C30 and QLQ-H&N35) assessment after head and neck radiotherapy. Radiother Oncol 2010;97:270-5. [↑](#footnote-ref-30)
31. Aaronson NK, Ahmedzai S, Bergman B, Bullinger M, Cull A, Duez NJ, Filiberti A, Flechtner H, Fleishman SB, de Haes JC, et al. The European Organization for Research and Treatment of Cancer QLQ-C30: a quality-of-life instrument for use in international clinical trials in oncology. J Natl Cancer Inst. 1993 Mar 3;85(5):365-76. [↑](#footnote-ref-31)
32. Feng, M, Moran, JM, Koelling, T, et al. Development and Validation of a Heart Atlas To Study Cardiac Exposure to Radiation Following Treatment for Breast Cancer. Int. J. Radiat. Oncol. Biol. Phys. 2011;79. [↑](#footnote-ref-32)
33. Effect of radiotherapy after breast-conserving surgery on 10-year recurrence and 15-year breast cancer death: meta-analysis of individual patient data for 10,801 women in 17 randomised trials. Early Breast Cancer Trialists' Collaborative Group (EBCTCG), Darby S, McGale P, Correa C, Taylor C, Arriagada R, Clarke M, Cutter D, Davies C, Ewertz M, Godwin J, Gray R, Pierce L, Whelan T, Wang Y, Peto R. 2011, Lancet, Vol. 378, pp. 1707-1716 [↑](#footnote-ref-33)
34. National Institute for Health and Clinical Excellence. Early and locally advanced breast cancer: diagnosis and treatment. London : National Institute for Health and Clinical Excellence, 2009. CG80. [↑](#footnote-ref-34)
35. Kogel, van der. Radiobiology of normal tissues. [book auth.] Steel GG. Basic Clinical Radiology. London : Arnold, 1993, pp. 99-107 [↑](#footnote-ref-35)
36. A longitudinal study of symptoms and self-care activities in women treated with primary radiotherapy for breast cancer. Knobf MT, Sun Y. 3, 2005, Cancer Nurs, Vol. 28, pp. 210-218 [↑](#footnote-ref-36)
37. Comparison of patient-reported breast, arm, and shoulder symptoms and body image after radiotherapy for early breast cancer: 5-year follow-up in the randomised Standardisation of Breast Radiotherapy (START) trials. Hopwood P, Haviland JS, Sumo G, Mills J, Bliss JM, Yarnold JR and Group, START Trial Management. 3, 2010, Lancet Oncology, Vol. 11, pp. 231-240 [↑](#footnote-ref-37)
38. Therapeutic mammaplasty--a systematic review of the evidence. McIntosh J, O'Donohgue JM. 3, 2012, Eur J Surg Oncol, Vol. 38, pp. 196-202 [↑](#footnote-ref-38)
39. UK National Mastectomy and Breast Reconstruction Audit, Fourth Report. Leeds : NHS Information Centre for Health and Social Care, 2011 [↑](#footnote-ref-39)
40. Radiotherapy and breast reconstruction: a meta-analysis. Barry M, Kell MR. 1, 2011, Breast Cancer Res Treat, Vol. 127, pp. 15-22 [↑](#footnote-ref-40)
41. A prospective longitudinal study of cosmetic outcome in immediate latissimus dorsi breast reconstruction and the influence of radiotherapy. Thomson HJ, Potter S, Greenwood RJ, Bahl A, Barker J, Cawthorn SJ, Winters ZE. 4, 2008, Ann Surg Oncol, Vol. 15, pp. 1081-1091 [↑](#footnote-ref-41)
42. Postoperative radiotherapy in high-risk premenopausal women with breast cancer who receive adjuvant chemotherapy. Danish Breast Cancer Cooperative Group 82b Trial. Overgaard M, Hansen PS, Overgaard J, Rose C, Andersson M, Bach F, Kjaer M, Gadeberg CC, Mouridsen HT, Jensen MB, Zedeler K. 14, 1997, N Engl J Med, Vol. 337, pp. 949-955 [↑](#footnote-ref-42)
43. Current status of autologous tissue-based breast reconstruction in patients receivigng postmastectomy radiation therapy. Kronowitz. 2012, Plast Reconstr Surg, Vol. 130, pp. 282-292 [↑](#footnote-ref-43)
44. Does variability in normal tissue reactions after radiotherapy have a genetic basis – where and how to look for it? Andreassen CN, Alsner J, Overgaard J. 2002, Radiother Oncol, Vol. 64, pp. 131-140 [↑](#footnote-ref-44)
45. Genetic variants and normal tissue toxicity after radiotherapy: a systematic review. Andreassen CN, Alsner J. 2009, Radiother Oncol, Vol. 92, pp. 299-309 [↑](#footnote-ref-45)
46. Independent validation of genes and polymorphisms reported to be associated with radiation toxicity: a prospective analysis study. Barnett GC, Coles CE, Elliott RM, Baynes C, Luccarini C, Conroy D, Wilkinson JS, Tyrer J, Misra V, Platte R, Gulliford SL, Sydes MR, Hall E, Bentzen SM, Dearnaley DP, Burnet NG, Pharoah PD, Dunning AM, West CM. 2012, Lancet Oncol, Vol. 13, pp. 65-77 [↑](#footnote-ref-46)
47. Establishment of a radiogenomics consortium. West CM, Rosenstein BS. 1, 2010, Radiother Oncol, Vol. 94, pp. 117-118 [↑](#footnote-ref-47)
48. A replicated association between polymorphisms near TNF-alpha and risk for adverse reactions to radiotherapy. Talbot CJ, Tanteles GA, Barnett GC, Burnet NG, Chang-Claude J, Coles CE, Davidson S, Dunning AM, Mills J, Murray RJ, Popanda O, Seibold P, West CM, Yarnold JR, Symonds RP. 2012, Br J Cancer, Vol. 107, pp. 748-753 [↑](#footnote-ref-48)
49. Association between single nucleotide polymorphisms in the DNA repair gene LIG3 and acute adverse skin reactions following radiotherapy. Murray RJ, Tanteles GA, Mills J, Perry A, Peat I, Osman A, Chan S, Cheung KL, Chakraborti PR, Woodings PL, Barwell JG, Symonds RP, Talbot CJ. 2, 2011, Radiother Oncol, Vol. 99, pp. 231-234 [↑](#footnote-ref-49)
50. A 2-stage genome-wide association study to identify single nucleotide polymorphisms associated with development of erectile dysfunction following radiation therapy for prostate cancer. Kerns SL, Stock R, Stone N, Buckstein M, Shao Y, Campbell C, Rath L, De Ruysscher D, Lammering G, Hixson R, Cesaretti J, Terk M, Ostrer H, Rosenstein BS. 1, 2013, Int J Radiat Oncol Biol Phys, Vol. 85, pp. e21-28 [↑](#footnote-ref-50)
51. Influence of irradiation on therapy-associated psychological distress in breast carcinoma patients. Mose S, Budischewski KM, Rahn AN, Zander-Heinz AC, Bormeth S, Böttcher HD. 5, 2001, Int J Radiat Oncol Biol Phys, Vol. 51, pp. 1328-1335 [↑](#footnote-ref-51)
52. Breast cancer patients' experience of external-beam radiotherapy. Schnur JB, Ouellette SC, Bovbjerg DH, Montgomery GH. 5, 2009, Qual Health Res, Vol. 19, pp. 668-676 [↑](#footnote-ref-52)
53. qualitative analysis of acute skin toxicity among breast cancer radiotherapy patients. Schnur JB, Ouellette SC, Dilorenzo TA, Green S, Montgomery GH. 3, 2011, Psychooncology, Vol. 20, pp. 260-268 [↑](#footnote-ref-53)
54. Qualitative research: grounded theory, mixed methods, and action research. Lingard L, Albert M, Levinson W. 2008, BMJ, Vol. 337, pp. 459-461 [↑](#footnote-ref-54)
55. How we design feasibility studies. Bowen DJ, Kreuter M, Spring B, Cofta-Woerpel L, Linnan L, Weiner D, Bakken S, Kaplan CP, Squiers L, Fabrizio C, Fernandez M. 5, 2009, Am J Prev Med, Vol. 36, pp. 452-457 [↑](#footnote-ref-55)
56. JP, Clark. How to peer review a qualitative manuscript. [book auth.] Jefferson T Godlee F. Peer review in health sciences. 2nd. s.l. : BMJ Books, 2003, pp. 219-235 [↑](#footnote-ref-56)
57. Using thematic analysis in psychology. Braun V, Clarke V. 2006, Qual Res Psychol, Vol. 3, pp. 77-101 [↑](#footnote-ref-57)
58. A. Sancar and others, "Circadian Clock, Cancer, and Chemotherapy," *Biochemistry* 54, no. 2 (2015). [↑](#footnote-ref-58)
59. G. C. Barnett and others, "A Genome Wide Association Study (Gwas) Providing Evidence of an Association between Common Genetic Variants and Late Radiotherapy Toxicity," *Radiother Oncol* 111, no. 2 (2014). [↑](#footnote-ref-59)
60. N. Douris and others, "Nocturnin Regulates Circadian Trafficking of Dietary Lipid in Intestinal Enterocytes," *Curr Biol* 21, no. 16 (2011). [↑](#footnote-ref-60)
61. J. J. Stubblefield, J. Terrien, and C. B. Green, "Nocturnin: At the Crossroads of Clocks and Metabolism," *Trends Endocrinol Metab* 23, no. 7 (2012). [↑](#footnote-ref-61)
62. M. von Schantz, "Phenotypic Effects of Genetic Variability in Human Clock Genes on Circadian and Sleep Parameters," *J Genet* 87, no. 5 (2008). [↑](#footnote-ref-62)
63. A. M. Gomes, R. C. Barber, and Y. E. Dubrova, "Paternal Irradiation Perturbs the Expression of Circadian Genes in Offspring," *Mutat Res* 775, (2015). [↑](#footnote-ref-63)
64. Ruscheweyh R, Marziniak M, Stumpenhorst F, Reinholz J, Knecht S. Pain sensitivity can be assessed by self-rating: Development and validation of the Pain Sensitivity Questionnaire. Pain, 2009;146(1–2): 65–74. [↑](#footnote-ref-64)
65. 65 Melzack R. The McGill Pain Questionnaire: Major properties and scoring methods. Pain, 1975; 1: 277-299.

    ^66^ Kerns, R.D., Turk, D.C., & Rudy, T.E. (1985). The West Haven-Yale Multidimensional Pain Inventory (WHYMPI). Pain, 23, 345-356

    ^67^ Zigmond AS, Snaith RP. The hospital anxiety and depression scale. Acta Psychiatr Scand,1983; 67(6): 361–70. [↑](#footnote-ref-65)
