# Supplementary material for: Treatment time and circadian genotype interact to influence radiotherapy side-effects. A prospective European validation study using the REQUITE cohort
Source: eBioMedicine. 2022 Sep 18;84:104269. doi: 10.1016/j.ebiom.2022.104269 (PMC9486558; doi:10.1016/j.ebiom.2022.104269)
Supplement: Supplementary file 6 [file mmc6.docx]

| Alison M | Dunning |
| --- | --- |
| Laura | Fachal |
| Dawn | Ennis |
| Petra | Seibold |
| Jenny | Chang-Claude |
| Thomas | Heger |
| Irmgard | Helmbold |
| Miguel E. | Aguado Barrera |
| Ana M. | Carballo |
| Antonio | Gómez-Caamaño |
| Begoña | Taboada-Lorenzo |
| Paloma | Sosa Fajardo |
| Patricia | Calvo-Crespo |
| Ramón | Lobato Busto |
| Ana | Vega |
| Paula | Peleteiro |
| Petra | Stegmaier |
| Johannes | Claßen |
| Thomas | Schnabel |
| Jörg | Schäfer |
| Tiziana | Rancati |
| Claudia | Sangalli |
| Marzia | Franceschini |
| Tommaso | Giandini |
| Riccardo | Valdagni |
| Alessandro | Cicchetti |
| Maria | Carmen De Santis |
| Eliana | La Rocca |
| Barbara | Avuzzi |
| Laura | Lozza |
| Gilles | Defraene |
| Maarten | Lambrecht |
| Patrick | Berkovic |
| Gert | De Meerleer |
| Karin | Haustermans |
| Dirk | De Ruysscher |
| Andreas | Rimner |
| Daniel S. | Higginson |
| Richard G. | Stock |
| Barry S | Rosenstein |
| Marie-Pierre | Farcy-Jacquet |
| Erik | Briers |
| Hilary | Stobart |
| Tim | Ward |
| Christel | Monten |
| Pieter | Deseyne |
| Liv | Veldeman |
| Renée | Bultijnck |
| Valérie | Fonteyne |
| Yolande | Lievens |
| Giselle | Post |
| Carsten | Herskind |
| Marlon R. | Veldwijk |
| Elena | Sperk |
| Kerstie | Johnson |
| R. Paul | Symonds |
| Adam | Webb |
| Rebecca M | Elliott |
| Christopher J | Talbot |
| Tim | Rattay |
| Ahmed | Salem |
| Ananya | Choudhury |
| Corinne | Faivre-Finn |
| Catharine ML | West |
| Holly | Summersgill |
| Zoe | Lingard |
| Alan | McWilliam |
| Pierre | Boisselier |
| Sylvain | Demontois |
| David | Azria |
| Celine | Bourgier |
| Muriel | Brengues |
| Roxana | Draghici |
| Sarah L | Kerns |
| Sara | Gutiérrez-Enríquez |
| Meritxell | Mollà |
| Mónica | Ramos |
| Alexandra | Giraldo |
| Victoria | Reyes |
| Alejandro | Seoane-Ramallo |
| Manuel | Altabas |
